# Mendelian randomization analysis does not reveal a causal influence between keratoconus and three major mental disorders

**DOI:** 10.3389/fpsyt.2024.1370670

**Published:** 2024-08-21

**Authors:** Xueyan Zhang, Qiaoling Wang, Fenghua Cui, Xuelian Wu, Chenming Zhang

**Affiliations:** Department of Ophthalmology, The Jinan Second People’s Hospital, Jinan, Shandong, China

**Keywords:** keratoconus, anxiety, depression, schizophrenia, Mendelian randomization, genome-wide association study

## Abstract

**Background:**

Observational studies have suggested at a possible link between keratoconus (KC) and various mental disorders, but the exact direction of causation in these associations remains unclear. This study aims to investigate the potential causal link between KC and three prominent mental conditions: Anxiety, Depression, and Schizophrenia.

**Methods:**

Using instrumental variables identified from Genome-wide association study (GWAS) data of European individuals, we conducted bidirectional two-sample Mendelian Randomization (MR) analyses to explore potential causal relationships between KC and the three major mental disorders. We primarily employed the Inverse-Variance Weighted (IVW) method to evaluate causality. In addition, we performed four supplementary MR methods (MR-Egger, Weighted Median, Simple Mode, and Weighted Mode). Furthermore, we conducted various sensitivity analyses to assess heterogeneity, horizontal pleiotropy, and result stability.

**Results:**

Our findings did not reveal any concrete evidence of a causal link between KC and the three major mental disorders, namely anxiety, depression, and schizophrenia [anxiety: odds ratio (OR)=0.997, 95% confidence interval (CI)=0.988–1.008, p = 0.621; depression: OR=1.008, 95% CI=0.999–1.017, p = 0.084; schizophrenia: OR=1.002, 95% CI= 0.984–1.020, p = 0.840]. Similarly, the three major mental disorders were not caustically associated with KC [anxiety: OR=1.014, 95% CI=0.635–1.620, p = 0.953; depression: OR=1.109, 95% CI= 0.749–1.643, p = 0.604; schizophrenia: OR= 0.969, 95% CI= 0.884–1.062, p = 0.497]. The sensitivity analyses indicated that the results remained robust, with no signs of pleiotropy or heterogeneity.

**Conclusions:**

Our study does not support a genetically determined significant causal connection between KC and the three major mental disorders. The increased occurrence of mental disorders observed in KC patients in observational reports likely arises from factors that can be modified. Further research is warranted to unveil the underlying mechanisms behind the associations observed in observational studies.

## Introduction

Keratoconus (KC) is a chronic, progressive condition affecting the cornea, typically arising during adolescence. It is usually manifested asymmetrically between the two eyes (1). Approximately 50% of clinically normal fellow eyes will progress to KC within 16 years (2). The typical slit-lamp signs are Fleischer’s ring, Vogt’s pattern, Munson’s sign or Rizzuti’s sign (3). Disease progression leads to progressive steepening of the anterior and posterior corneal surfaces and progressive thinning of corneal thickness, resulting in irregular astigmatism and progressive myopia with decreased vision but difficult to correct. In advanced stages, keratoconus may present with acute corneal edema, and corneal scarring occurs after the edema resolves, leading to severe visual impairment (4). This disease is a major indication for corneal transplantation in many countries (5).

Studies have reported an incidence of 54 cases per 100,000 in the general population, with a higher prevalence among Asian populations (6). The prevalence of keratoconus varies between ethnic groups, with figures as high as 1.2% reported in some predominantly European populations, to 2.3–3.3% in Maori or Iranian populations (7–9). The interplay of genetics and environmental factors significantly contributes to the pathogenesis of KC (10).

Onset typically manifests during adolescence, with 94% of patients diagnosed between the ages of 12 and 39 (11). This age range corresponds to a critical period encompassing physical, cognitive, psychosocial development, and future planning (12). The presence of a chronic condition during this phase may adversely affect an individual’s life and psychological well-being (13).

A number of studies have reported the problem of comorbidity of keratoconus with psychiatric disorders. A questionnaire survey was conducted in 84 keratoconus patients and 63 normal subjects. They found increased levels of schizophrenia in keratoconus patients and a higher incidence of depression in female subjects (14). In another study, 35 (37.2%) of 94 keratoconus patients had a psychiatric diagnosis. 13 (13.8%) patients had moderate to severe depression, and 20 (21.2%) patients had moderate to severe anxiety (15). Increased binocular asymmetry, decreased visual acuity and corneal steepness in the better eye are associated with decreased vision-related quality of life in patients with keratoconus (16). Compared with patients with stable disease, vision-related quality of life (vr-QoL) in patients with advanced KC was statistically significant in terms of “mental health”, “role difficulty” and “dependence” (17). A steep keratometric reading was associated with lower scores on the Mental Health, Role Difficulty, Driving, Dependency, and Ocular Pain scales (18). Anxiety, exacerbated by frequent eye rubbing in some patients, may influence the progression or onset of keratoconus. In turn, the advancement of keratoconus can affect the behavior and thought processes of patients (19, 20). Nevertheless, most of these reports are observational, subject to confounding variables, making it challenging to establish a definitive causal link between KC and anxiety, depression, or schizophrenia.

Mendelian randomization (MR) analysis employs a simulated randomized controlled trial design, utilizing genetic variants as instrumental variables for exposure to ascertain causality between exposure and outcomes (21). This approach is immune to the limitations that often undermine traditional study designs and can reduce potential confounding. In our study, we conducted a two-sample MR analysis based on extensive genome-wide association studies (GWAS) data to explore potential causal relationships between KC and anxiety, depression, and schizophrenia.

## Materials and methods

### Data source

The genome-wide association studies (GWAS) summary statistics for KC were obtained from a recent genome-wide association meta-analysis study that included 2,116 European KC patients and 24,626 healthy controls (22). These data were downloaded from the GWAS catalog website (https://www.ebi.ac.uk/gwas/downloads/summary-statistics). The summary statistics for anxiety (N =5,495 cases; 11,775 controls) were provided by the Anxiety NeuroGenetics Study Consortium (23), depression (N =170,756 cases; 329,443 controls) were sourced from 33 studies within the Psychiatric Genomics Consortium (PGC) 139 k and the United Kingdom Biobank (24), and schizophrenia (N = 53,386 cases; 77,258 controls) were retrieved from the Psychiatric Genomics Consortium (25). As both healthy controls with keratoconus and part of the population with depression were from the UK Biobank. We calculated the sample overlap rate (4.9%). We considered the risk of bias due to sample overlap to be very small (26).

To validate our analysis, summary statistics of the anxiety (N = 24,662 cases; 337,577 controls), depression (N =43,280 cases; 329,192 controls), and schizophrenia (N = 13,061 cases; 277,526 controls) data sets were accessed from the FinnGen consortium R9 release (27). All study participants were of European descent. Detailed information regarding the GWAS summary-level data in this MR study can be found in the **Supplementary Material**. All data analyzed in this study were obtained from publicly available databases in which ethics approval and patient consent can be identified in the original studies.

### Selection of instrumental variables

**Figure 1:**
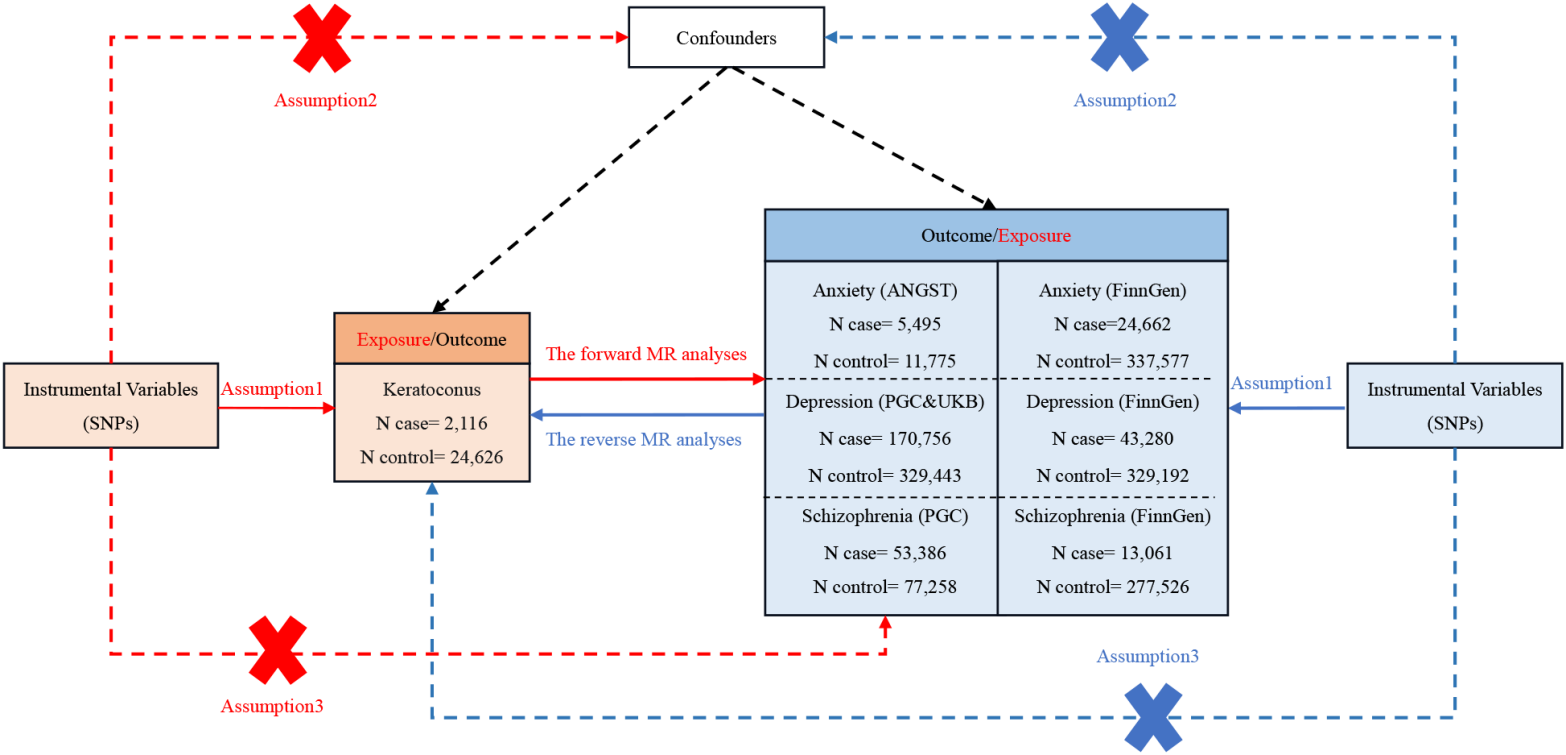
The comprehensive flowchart detailing the bidirectional MR study. SNP, single-nucleotide polymorphism; ANGST, the Anxiety NeuroGenetics Study Consortium; PGC, the Psychiatric Genomics Consortium; UKB, the UK Biobank Psychiatric Genetics Group.

The instrumental variables (IVs) were chosen based on three fundamental assumptions: (1) The relevance assumption, where IVs needed to be strongly associated with the exposure; (2) The independence assumption, ensuring that IVs were independent of confounders; and (3) The exclusion restriction assumption, whereby IVs could only affect the outcome through the exposure (28). The overall flow chart of the bidirectional MR study is displayed in **Figure 1**. The IVs for MR analysis were initially screened using a genome-wide significance threshold (P < 5 x 10^-8^). As the SNPs related to anxiety were fewer than 3, we used the genetic instruments on a lower significance threshold (P < 5 x 10^-6^) to get more SNPs. From the FinnGen consortium, SNPs for anxiety, depression, and schizophrenia were similarly chosen with the threshold of 5 x 10^-6^ to get more SNPs. Subsequently, the candidate IV set underwent the removal of SNPs with high linkage disequilibrium (LD), based on the parameter (r^2^ > 0.001, window size = 10,000 kb). Any ambiguous or palindromic SNPs and SNPs not present in the outcome GWAS summary data were then excluded from the IVs. Additionally, the PhenoScanner tool (http://www.phenoscanner.medschl.cam.ac.uk/) (29) was employed to identify and exclude any SNPs associated with the confounding factor of the outcome (the common confounding factors of KC as an outcome: body mass index, rubbing eyes, pollen, asthma, allergies, eczema, dry eye and **s**moking) (30–32). The F-statistic for each SNP was calculated as follows: F = R^2^ (N−2)/(1−R^2^). R^2^ was calculated as follows: R^2 =^ 2* (1−MAF) *MAF*(β)^2^. R^2^ is the cumulative explained variance of the selected IVs on exposure. MAF is the effect of allele frequency. β is the estimated effect of SNP. N is the sample size of the GWAS. SNPs with the F-statistic>10 were utilized to exclude the bias produced by weak instruments and ensure statistical efficiency (28).

### Statistical analysis

The MR analysis was initiated with the IVs selected under the genome-wide significance threshold. Data harmonization was then employed to ensure the consistent direction of SNP alleles between the exposure and outcomes. Five methods were utilized for MR analysis: inverse-variance weighted (IVW), MR-Egger, weighted median, simple mode, and weighted mode. The primary analytical method for estimating causal effects was the inverse-variance weighted (IVW) approach. A significance threshold of P < 0.05 was set, and the results of causal associations were presented as odds ratios (OR) along with their corresponding 95% confidence intervals (95% CI).

Subsequently, a series of sensitivity analyses were conducted. Heterogeneity was assessed using Cochran’s Q test. Horizontal pleiotropy was detected through the MR-Egger intercept and MR-PRESSO global tests. The influence of each SNP on the results was evaluated via a leave-one-out analysis, where one SNP was eliminated at a time. The MR Steiger filtering method was also applied to confirm causality.

All statistical analyses were executed using R software version 4.3.0, leveraging the ‘TwoSampleMR’ package and the ‘MR PRESSO’ package.

## Results

### Mendelian randomization analysis of KC on anxiety, depression and schizophrenia

**Figure 2:**
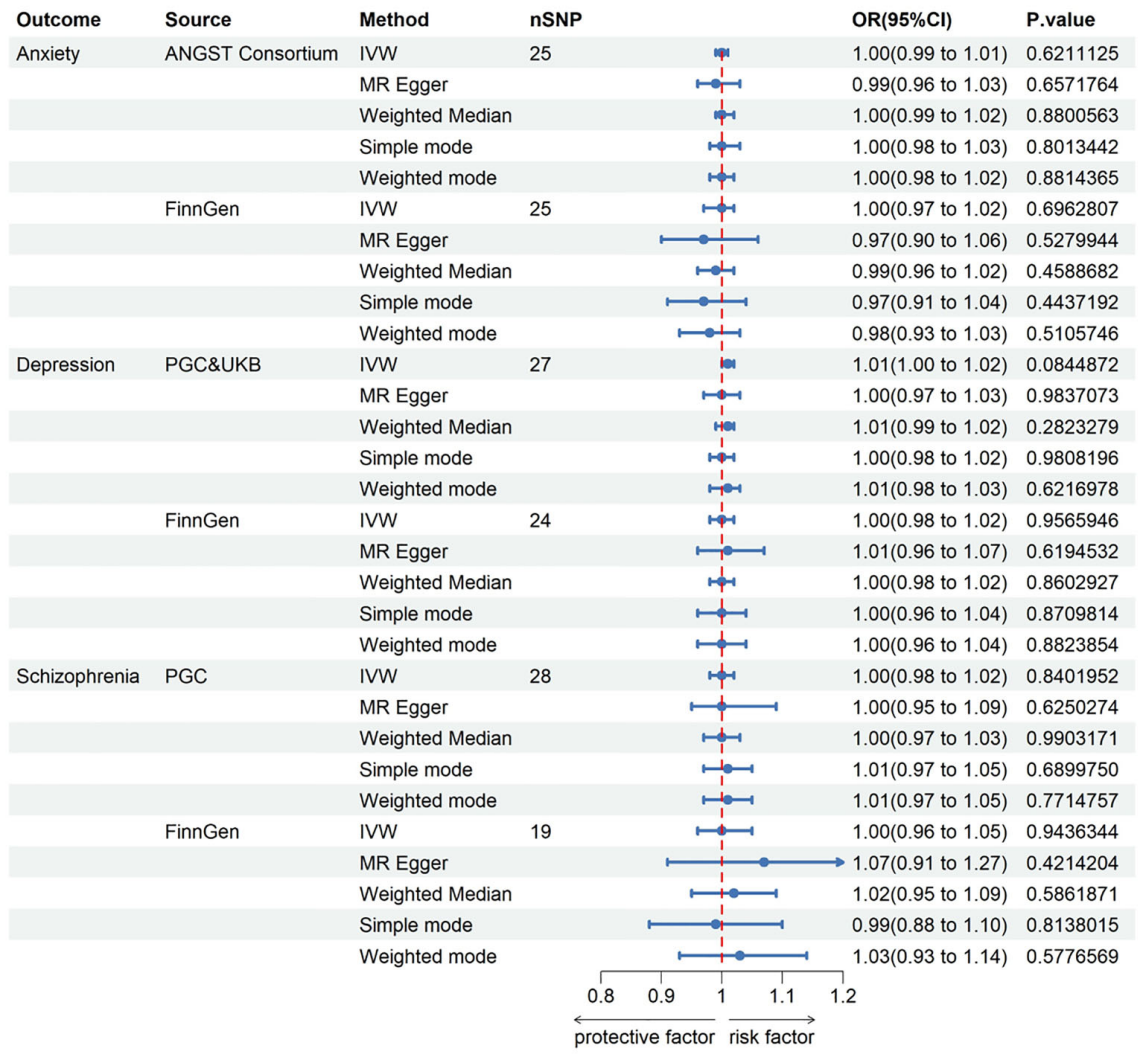
The Forest plot in this figure provides a visual representation of the estimates regarding the association of keratoconus with anxiety, depression, and schizophrenia. CI, confidence interval; OR, odds ratio. IVW, inverse-variance weighted; ANGST, the Anxiety NeuroGenetics Study Consortium; PGC, the Psychiatric Genomics Consortium; UKB, the UK Biobank Psychiatric Genetics Group.

The instrumental genetic variables employed for KC can be found in the **Supplementary Material**. The MR results presented here were predicated on IVs selected under the genome-wide significance threshold (P < 5 × 10^−8^). In this context, the assessment of the causal effects of KC on anxiety, depression, and schizophrenia was based on 25, 27, and 28 IVs, respectively. Nevertheless, none of the MR analyses revealed a propensity for KC to elevate the risk of these mental disorders. Genetically instrumented KC exhibited no significant effects on anxiety [odds ratio (OR): 0.997, 95% confidence interval (CI): 0.988–1.008; p = 0.621], depression (OR: 1.008, 95% CI: 0.999–1.017; p = 0.084), or schizophrenia (OR: 1.002, 95% CI: 0.984–1.020; p = 0.840). To validate these findings, we conducted additional assessments using data from the FinnGen consortium, which independently confirmed the absence of causal associations between KC and anxiety, depression, and schizophrenia. Detailed results for all methods are displayed in **Figure 2** and the **Supplementary Material**.

### Results of reverse Mendelian randomization analysis

**Figure 3:**
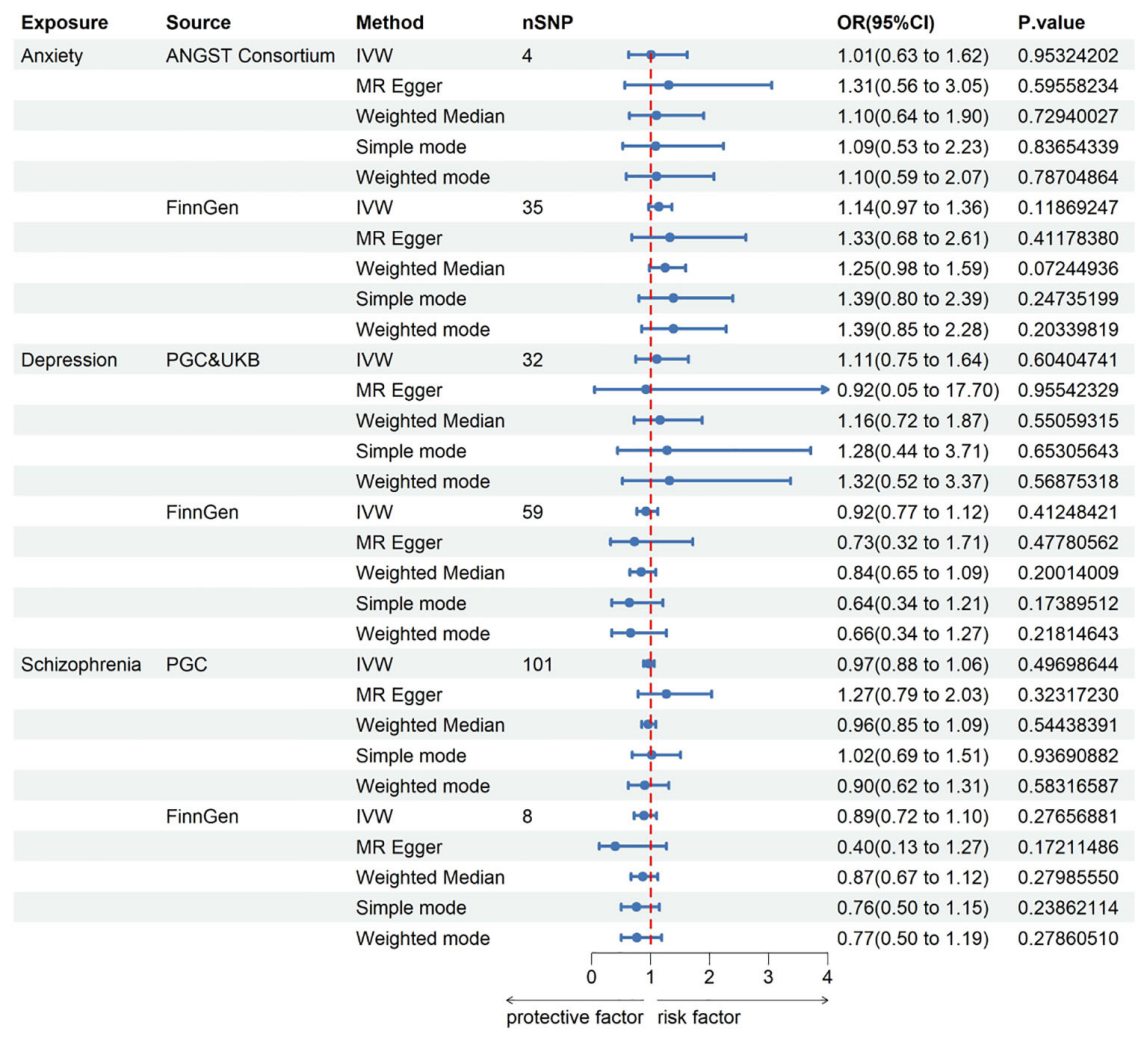
This Forest plot offers insights into the estimates concerning the association of anxiety, depression, and schizophrenia with keratoconus. CI, confidence interval; OR, odds ratio. IVW, inverse-variance weighted; ANGST, the Anxiety NeuroGenetics Study Consortium; PGC, the Psychiatric Genomics Consortium; UKB, the UK Biobank Psychiatric Genetics Group.

In pursuit of a reverse MR analysis, we aimed to determine whether anxiety, depression, or schizophrenia had a causal influence on KC. The IVs for depression and schizophrenia were screened using a genome-wide significance threshold (P < 5 x 10^-8^), with the exception of anxiety, where SNPs were selected under a threshold of 5 x 10^-6^. The assessment of the causal effects of anxiety, depression, and schizophrenia on KC was based on 4, 32, and 101 IVs, respectively. The instrumental genetic variables utilized can be found in the **Supplementary Material**. Regrettably, all MR analyses failed to establish a link between anxiety, depression, or schizophrenia and an increased risk of KC. Genetically instrumented anxiety [odds ratio (OR): 1.014, 95% confidence interval (CI): 0.635–1.620; p = 0.953], depression (OR: 1.109, 95% CI: 0.749–1.643; p = 0.604), and schizophrenia (OR: 0.969, 95% CI: 0.884–1.062; p = 0.497) exhibited no discernible effects on KC. Likewise, no causal associations were identified between anxiety, depression, and schizophrenia and KC in the independent FinnGen sample. Detailed results for all methods can be found in **Figure 3** and the **Supplementary Material**.

### Sensitivity analysis

In order to gauge the reliability of the MR results, we conducted sensitivity analyses. The MR Steiger test consistently affirmed that the inferred causal direction between exposure and outcome was indeed TRUE Cochran’s Q test yielded results indicating the absence of heterogeneity among the IVs employed in our MR analysis (P > 0.05). Furthermore, both the MR-Egger intercept test and the MR-PRESSO global test indicated no susceptibility to the potential influence of horizontal pleiotropy (P > 0.05). Visual assessment of funnel plots unveiled no discernible bias. Finally, the leave-one-out sensitivity analysis served to reinforce the robustness of the MR results, as the exclusion of any particular leading SNP did not significantly alter the outcome. A comprehensive compilation of results from the sensitivity analyses is accessible in the **Supplementary Material**.

## Discussion

To the best of our knowledge, this is the initial study to employ two-sample MR analysis in assessing the potential causal link between KC and three major mental disorders. This study comprehensively utilized the most extensive GWAS summary statistics available to probe the causal association between KC and anxiety, depression, and schizophrenia. Our findings cast doubt on the existence of a definitive causal relationship between KC and these significant mental disorders.

KC is a complex disease with multifactorial etiology. Genetic and environmental factors work together (10). Although in most cases, KC is reported as a sporadic disease. But in some cases there will be a autosomal recessive and autosomal dominant inheritance pattern, suggesting that genetic factors play a role in KC (33). However, the penetrance was reduced (34). Reduced penetrance means that some individuals with susceptible genotypes may not develop disease. This may be due to environmental or random interactions (35). Atopic physique, rubbing eyes and exposure to UV are some recognized environmental factors (36). As KC corneas lack antioxidant enzymes, they cannot remove or neutralize reactive oxygen species (ROS) (37). An excess of any of these environmental factors can cause oxidative damage to the KC cornea, which leads to a degradation process that ultimately leads to corneal thinning and vision loss (33). As many favor the “two-hit hypothesis,” it is assumed that keratoconus is the result of an underlying genetic predisposition, which is then triggered by sudden factors in the environment (38).

Previous observational studies have pointed to an association between keratoconus and these three major mental disorders. A study of patients with previously diagnosed keratoconus in Saudi Arabia and the Middle East showed that most patients (51%) were diagnosed with a psychiatric illness before keratoconus was diagnosed. Among them, 63.2% had anxiety disorders, 56.1% had depression, and 10.5% had schizophrenia. It is suggested that keratoconus patients suffer from an underlying susceptibility to psychiatric disorders, rather than psychiatric disorders as a result of the chronicity of the disease (39). In a recent cross-sectional study of the incidence of psychiatric disorders in keratoconus patients, the severity of keratoconus was measured using three different measures. The more severe the keratoconus is, the psychiatric diagnoses were significantly higher (15). However, other studies have denied the existence of a link between keratoconus and depression (40). Similarly, we did not find a causal genetic association between keratoconus and the three major psychiatric disorders. This implies that the phenotypic associations between keratoconus and the three major mental disorders are due to other mechanisms. We hypothesized that the cause of the correlation between Keratoconus and mental disorders may be linked to oxidative stress. Accumulating evidence suggests that oxidative stress plays a role in the development of KC. Studies have shown that the transcriptional levels and/or activities of different antioxidant enzymes are disturbed in KC corneas (33). The reduction of antioxidant enzymes leads to increased levels of reactive oxygen species (ROS)and reactive nitrogen species (RNS), possibly leading to degradation of the extracellular matrix in the stroma, which results in thinning of the stroma in KC corneas (41). Similarly, Oxidative stress mechanism related to the pathogenesis of mental disorders. Levels of the major antioxidant enzymes, superoxide dismutase (SOD), catalase (CAT), and glutathione peroxidase (GSH-Px), were decreased in patients with schizophrenia compared with controls (42, 43). Data from animal models and human studies have reported many oxidation disorder associated with depression and anxiety (44–47). Studies have proposed hypotheses to explain the co-occurrence of keratoconus and schizophrenia, including having similar underlying pathophysiology, such as immune inflammation and oxidative stress. And possible common genetic components, including variants on 13q32 and loci mapped to chromosome 21 (48, 49). The genetic mechanism of keratoconus with depression and anxiety has not been reported.

In addition, established risk factors and treatments for keratoconus may be confounding factors in observational studies. Anxiety and/or depression often co-occur with atopic diseases (such as allergies, asthma) occur at the same time (50, 51). Because eye rubbing is a recognized and possibly an independent nonpsychological variable in keratoconus, it is difficult to define a consistent psychological association between eye rubbing and keratoconus (52). The treatment of keratoconus mainly includes hard contact lenses, corneal cross-linking (CXL), and corneal transplantation (53). Studies have shown decreased anxiety in KC patients 1 year after successful CXL treatment. However, this intervention did not appear to influence the depressive status of KC patients (54). In the candidates for keratoplasty in KC patients, quality of life was reduced. The symptoms of depression and anxiety were present. The severity of depressive symptoms depends on the best corrected visual acuity in the better eye. But the postoperative improvement of visual acuity in the transplanted eye alleviated symptoms of depression and anxiety (55).

Nonetheless, the existing body of literature predominantly consists of observational studies. In contrast, our study employed “two-sample MR analysis” to explore the bidirectional causal relationship between KC and the three major mental disorders from a genetic perspective. The inclusion of heterogeneity and sensitivity analyses, coupled with diverse Mendelian tools, bolsters the stability of our results and ensures an accurate understanding of the causal effects (if any) between KC and anxiety, depression, and schizophrenia.

Nevertheless, there are certain limitations to this study. The study’s participants were of European ancestry, warranting further data collection and analysis to ascertain whether the results can be extrapolated to other populations. Secondly, the limited size of the GWAS database for keratoconus could potentially introduce bias into the results. Lastly, despite our efforts to control for relevant confounders, the potential influence of horizontal pleiotropy on our findings cannot be entirely ruled out.

In conclusion, our study does not support a genetically determined significant causal connection between KC and the three major mental disorders. The increased occurrence of mental disorders observed in KC patients in observational reports likely arises from factors (such as shared pathophysiological mechanisms and environmental factors) that can be modified. Other well-designed studies that minimize observational study bias and duplicate efforts of MR Analyses with larger GWAS data are warranted to reveal the underlying mechanisms behind the association between the two diseases. As noted by the CLEK study group, keratoconus is a relatively low-prevalence disease that rarely leads to blindness. But because it affects young people, the magnitude of its public health impact is out of proportion to its prevalence and clinical severity. (18). It has been observed that patients with eye disease generally do not report mental or emotional problems related to eye disease to their doctors. Progressive vision loss, the need for extended clinical follow-up, and the limited availability of costly interventions can trigger depression in KC patients. Depression can lead to poor treatment adherence and the progress of the irreversible loss of vision, which results in a more pronounced depressive state (56). These findings suggest that attention to mental disorders in KC patients is important. Ophthalmologists should consider the patient’s mental state when treating these patients and, if necessary, consider psychiatric consultation and psychotherapy in order to obtain better treatment results.

## Data Availability

The original contributions presented in the study are included in the article/**Supplementary Material**. Further inquiries can be directed to the corresponding authors.
