# Supplementary material for: Mendelian randomization analysis does not reveal a causal influence between keratoconus and three major mental disorders
Source: Front Psychiatry. 2024 Aug 21;15:1370670. doi: 10.3389/fpsyt.2024.1370670 (PMC11371735; doi:10.3389/fpsyt.2024.1370670)
Supplement: Supplementary file 1 [file DataSheet1.pdf]

Figure S1. MR effect size, funnel plot, Leave-one-out analysis, and scatter plot for Keratoconus on Anxiety(ANGST).

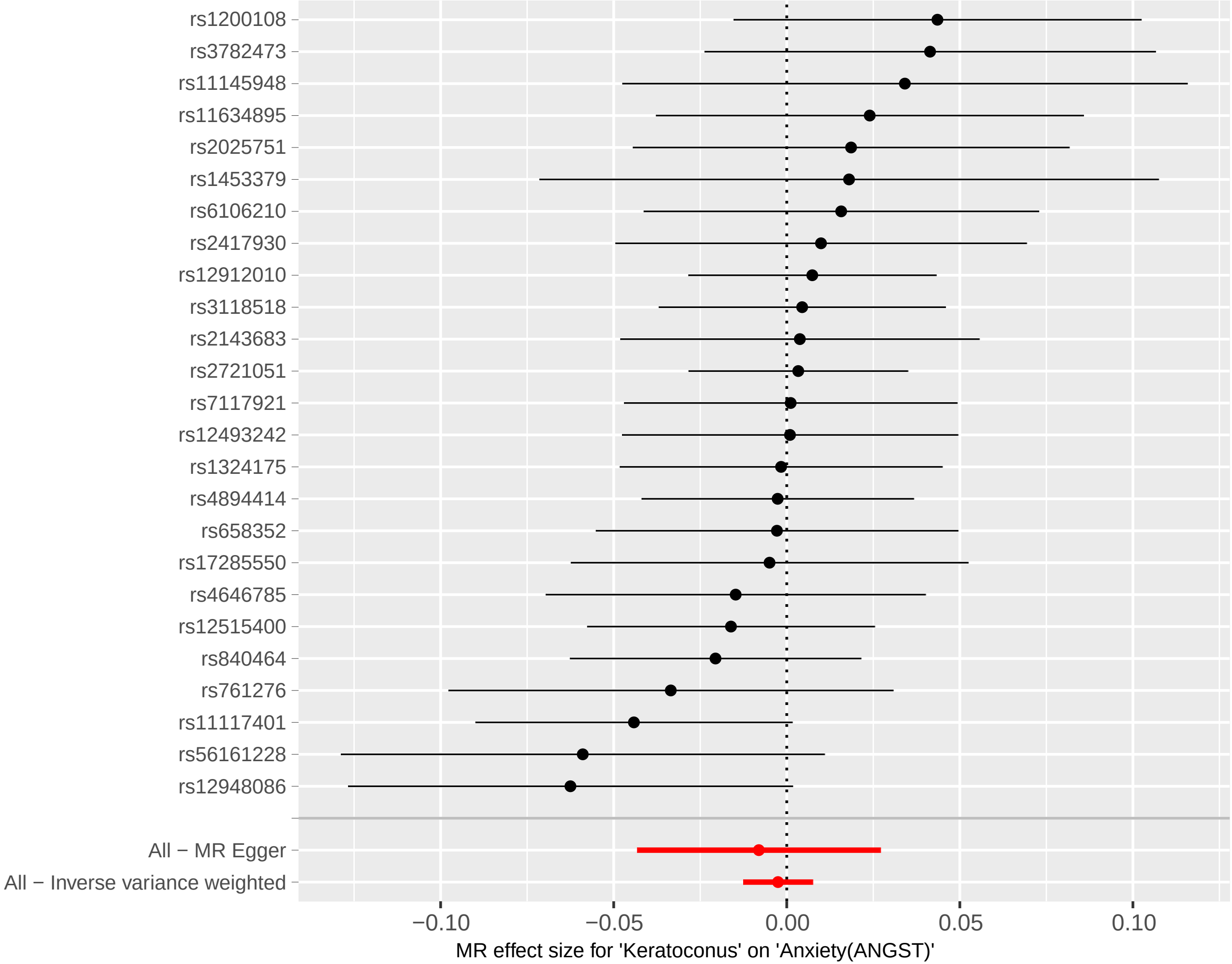

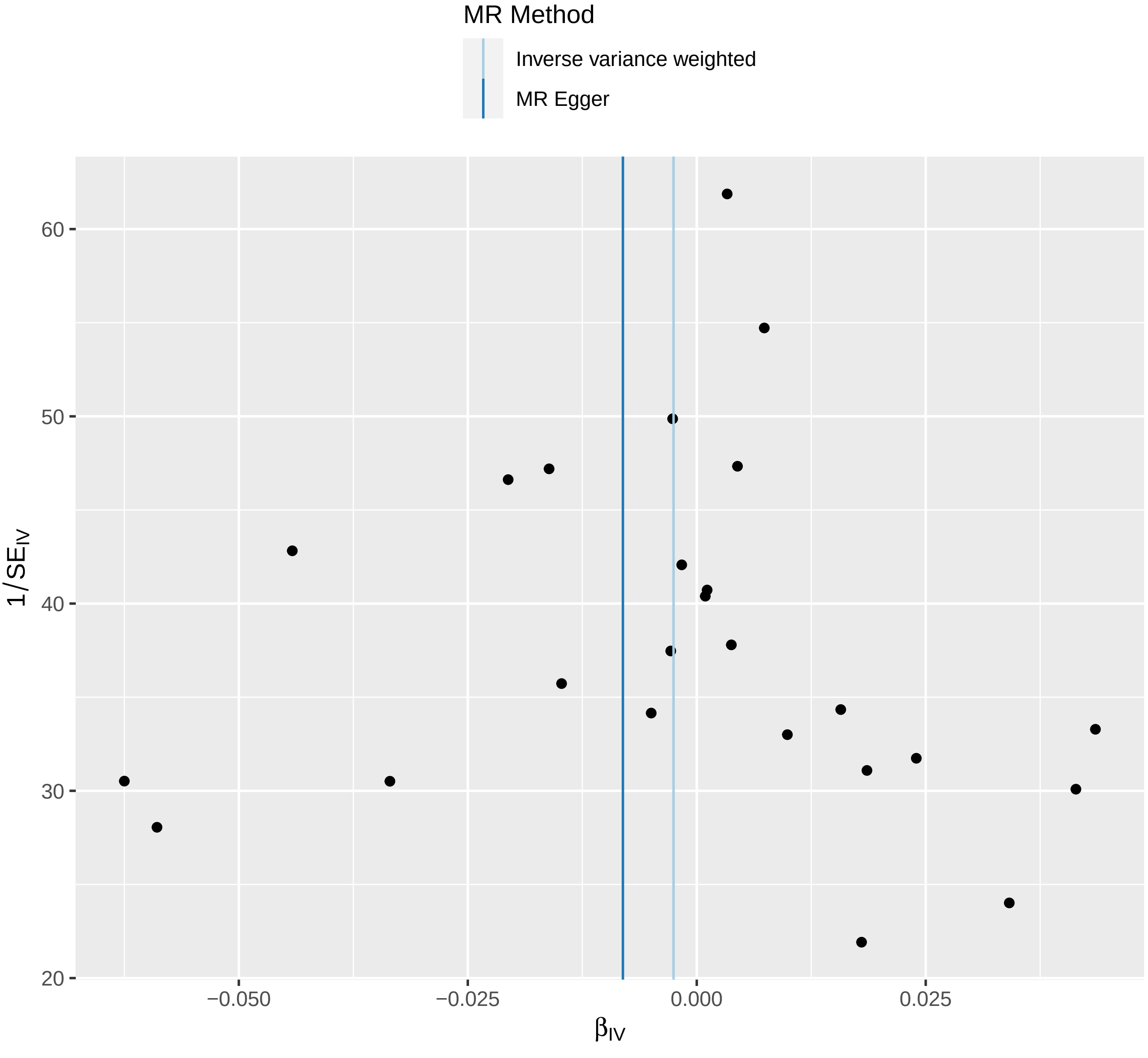

MR Method
Inverse variance weighted
MR Egger
60
50
40
30
20
$1/SE_{IV}$
−0.050
−0.025
0.000
0.025
β_{IV}

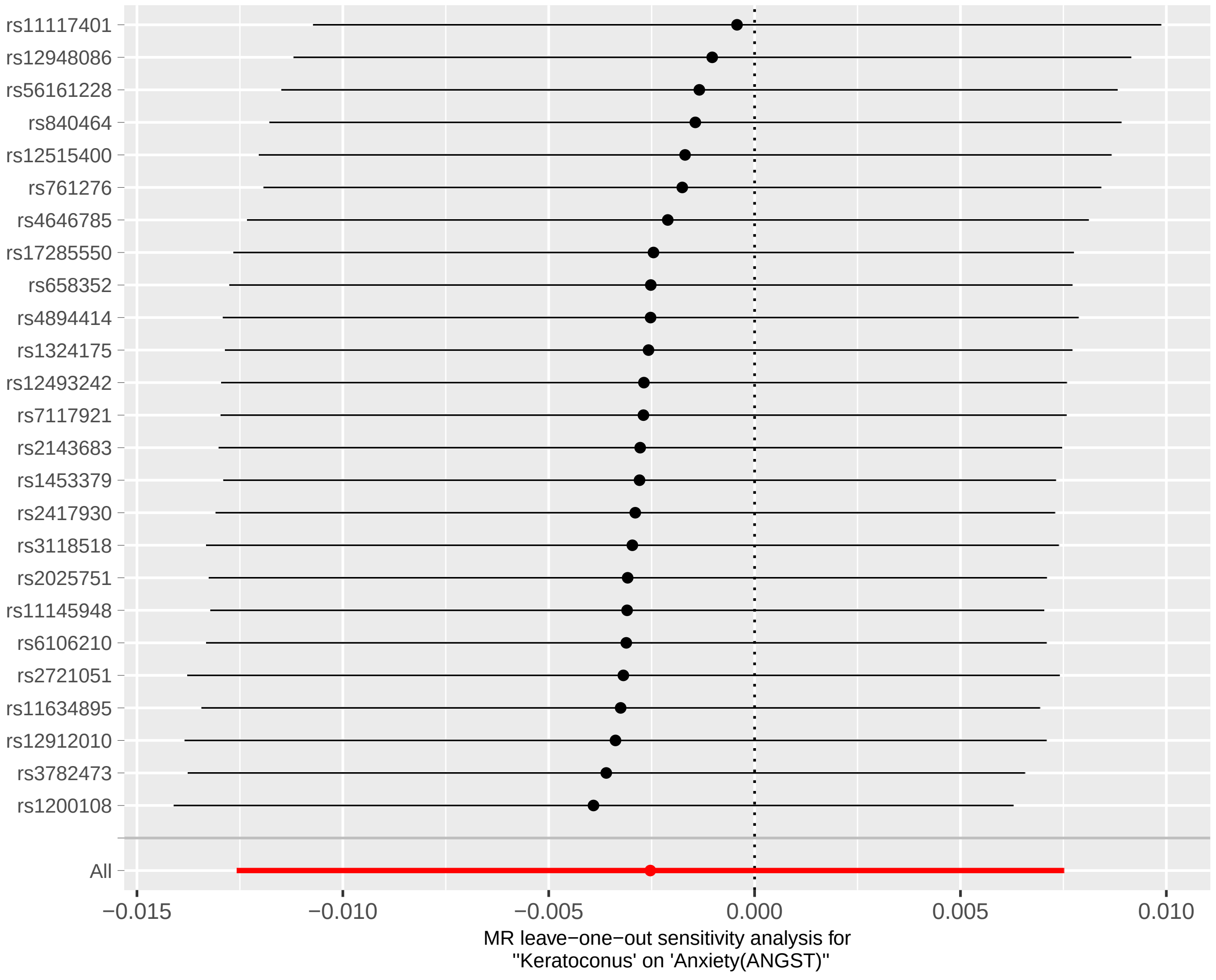

rs11117401
rs12948086
rs56161228
rs840464
rs12515400
rs761276
rs4646785
rs17285550
rs658352
rs4894414
rs1324175
rs12493242
rs7117921
rs2143683
rs1453379
rs2417930
rs3118518
rs2025751
rs11145948
rs6106210
rs2721051
rs11634895
rs12912010
rs3782473
rs1200108
All
−0.015
−0.010
−0.005
0.000
0.005
0.010
MR leave−one−out sensitivity analysis for
''Keratoconus' on 'Anxiety(ANGST)''

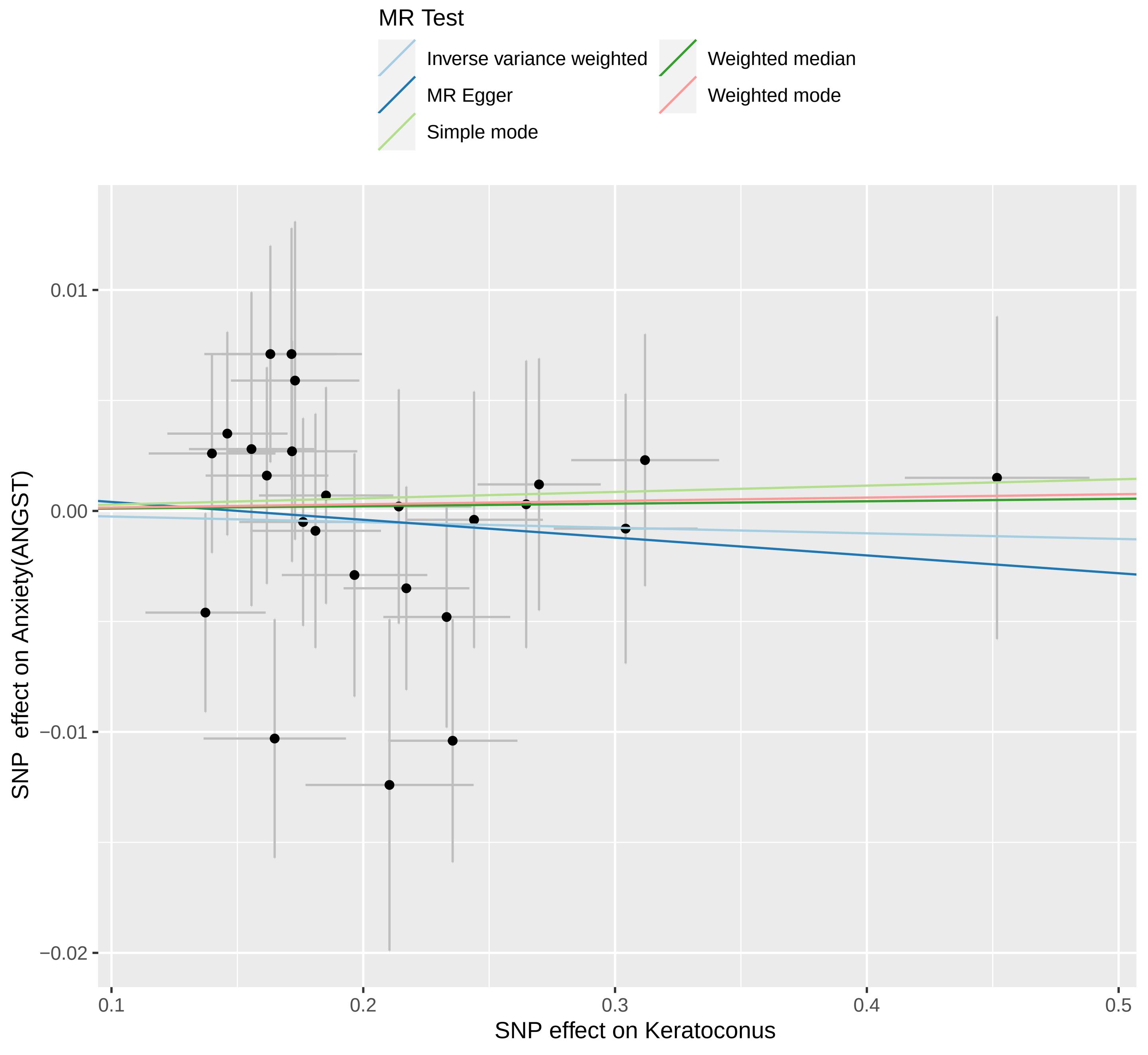

MR Test
Inverse variance weighted
MR Egger
Simple mode
Weighted median
Weighted mode
0.01
0.00
−0.01
−0.02
0.1
0.2
0.3
0.4
0.5
SNP effect on Keratoconus
SNP effect on Anxiety(ANGST)

Figure S2. MR effect size, funnel plot, Leave-one-out analysis, and scatter plot for Keratoconus on Anxiety(FinnGen).

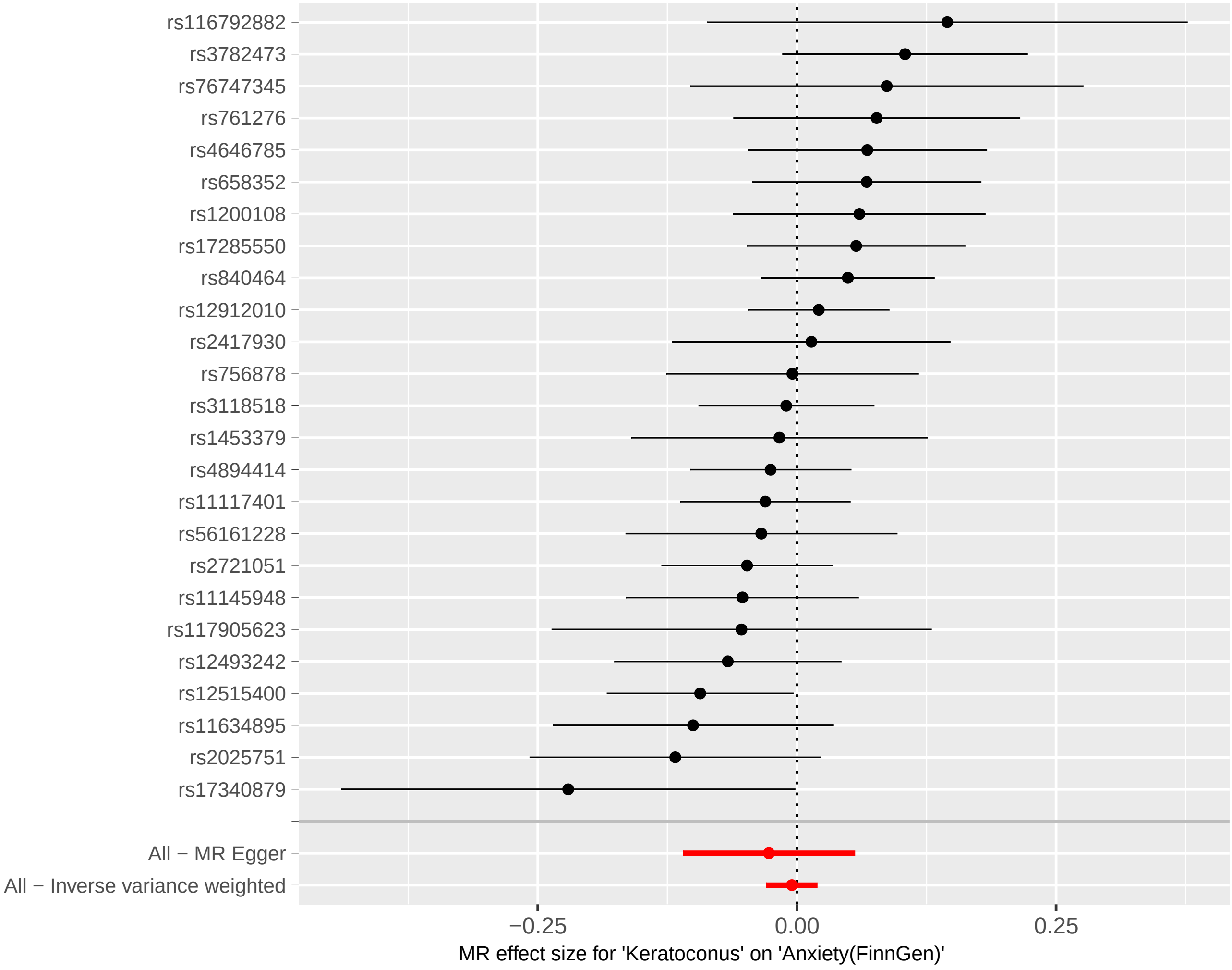

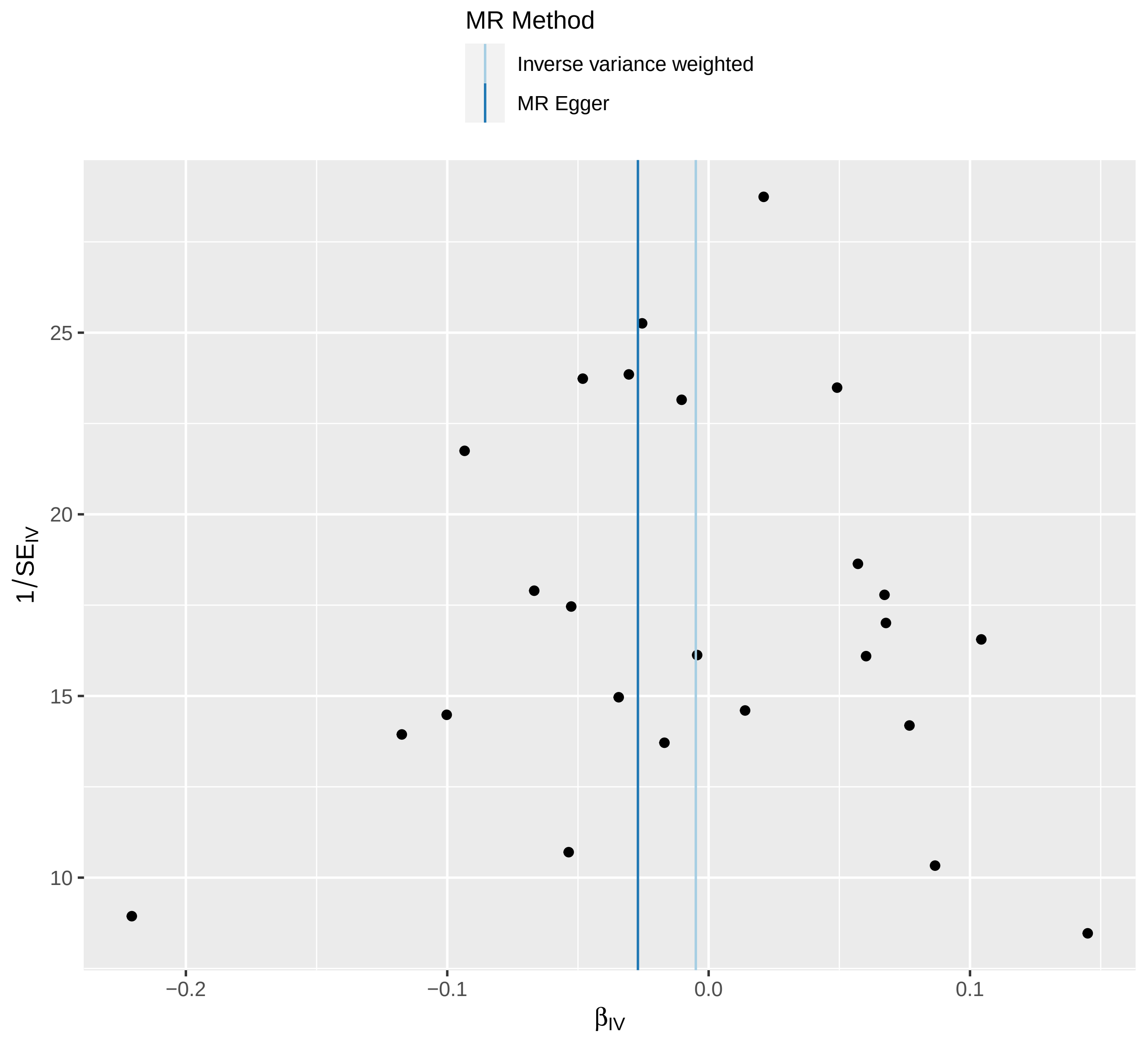
MR Method
Inverse variance weighted
MR Egger
25
20
15
10
1/SE_IV
−0.2
−0.1
0.0
0.1
β_IV

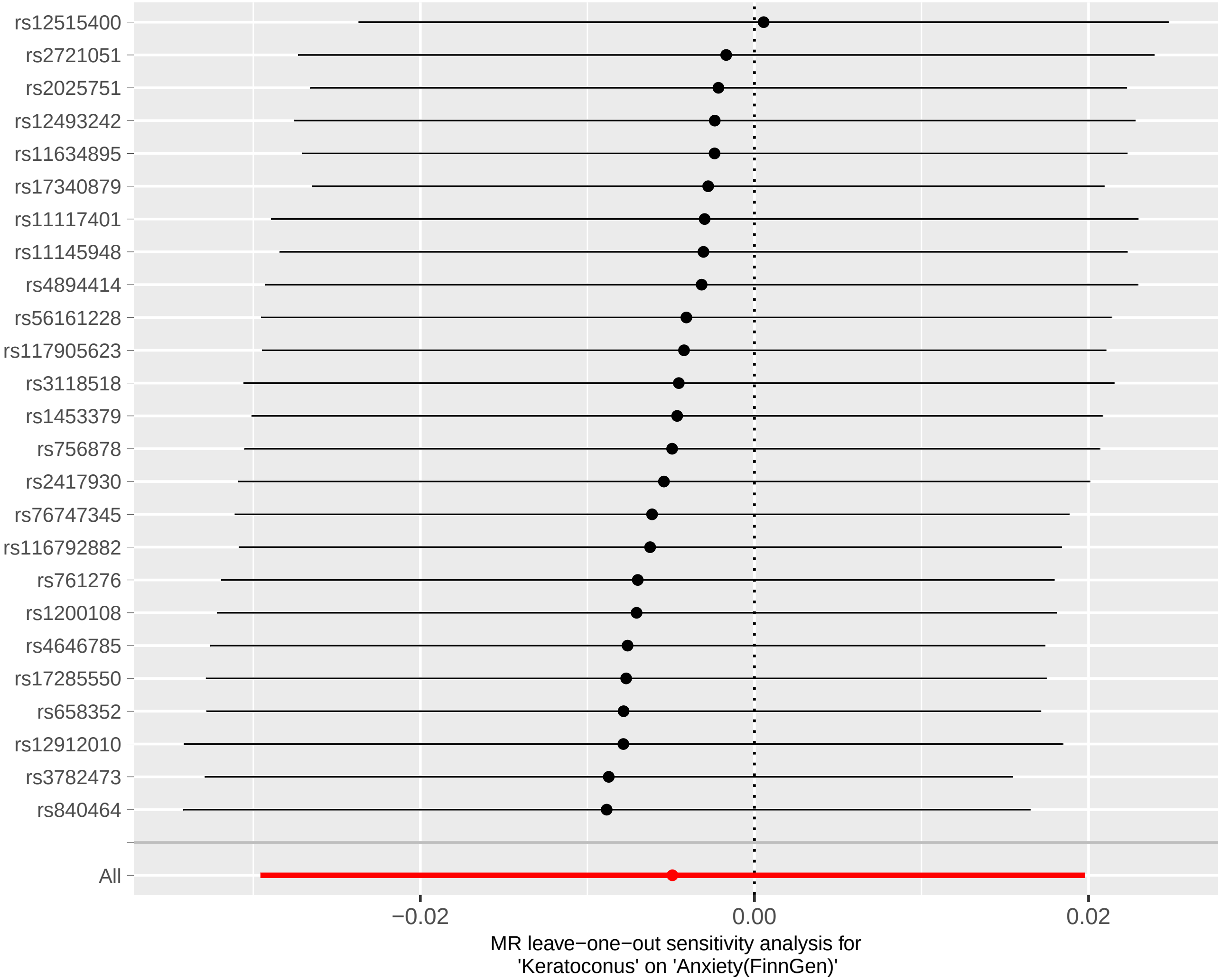
rs12515400
rs2721051
rs2025751
rs12493242
rs11634895
rs17340879
rs11117401
rs11145948
rs4894414
rs56161228
rs117905623
rs3118518
rs1453379
rs756878
rs2417930
rs76747345
rs116792882
rs761276
rs1200108
rs4646785
rs17285550
rs658352
rs12912010
rs3782473
rs840464
All
−0.02
0.00
0.02
MR leave−one−out sensitivity analysis for
'Keratoconus' on 'Anxiety(FinnGen)'

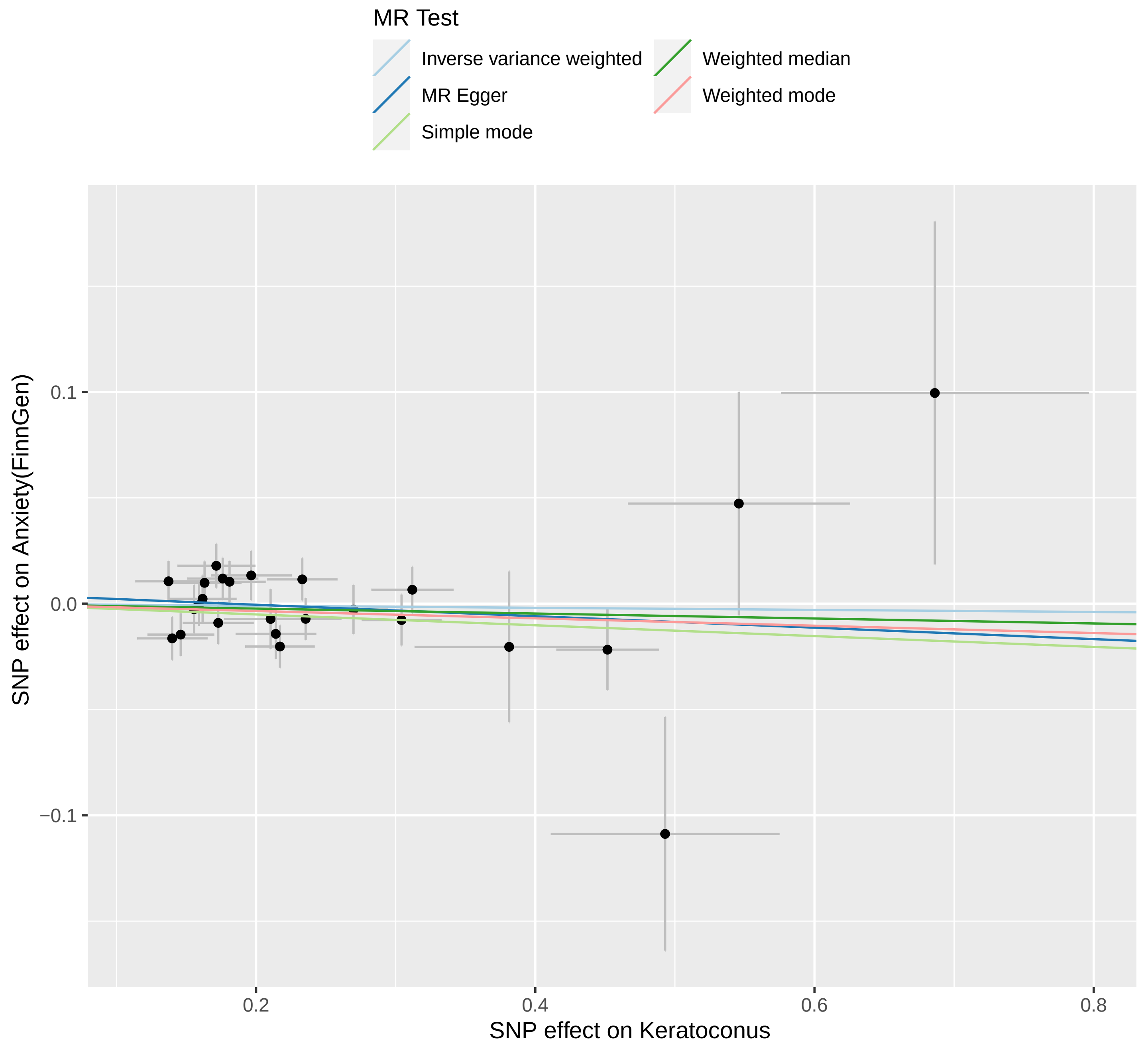
MR Test
Inverse variance weighted
MR Egger
Simple mode
Weighted median
Weighted mode
SNP effect on Anxiety(FinnGen)
0.1
0.0
−0.1
0.2
0.4
0.6
0.8
SNP effect on Keratoconus

Figure S3. MR effect size, funnel plot, Leave-one-out analysis, and scatter plot for Keratoconus on Depression(PGC&UKB).

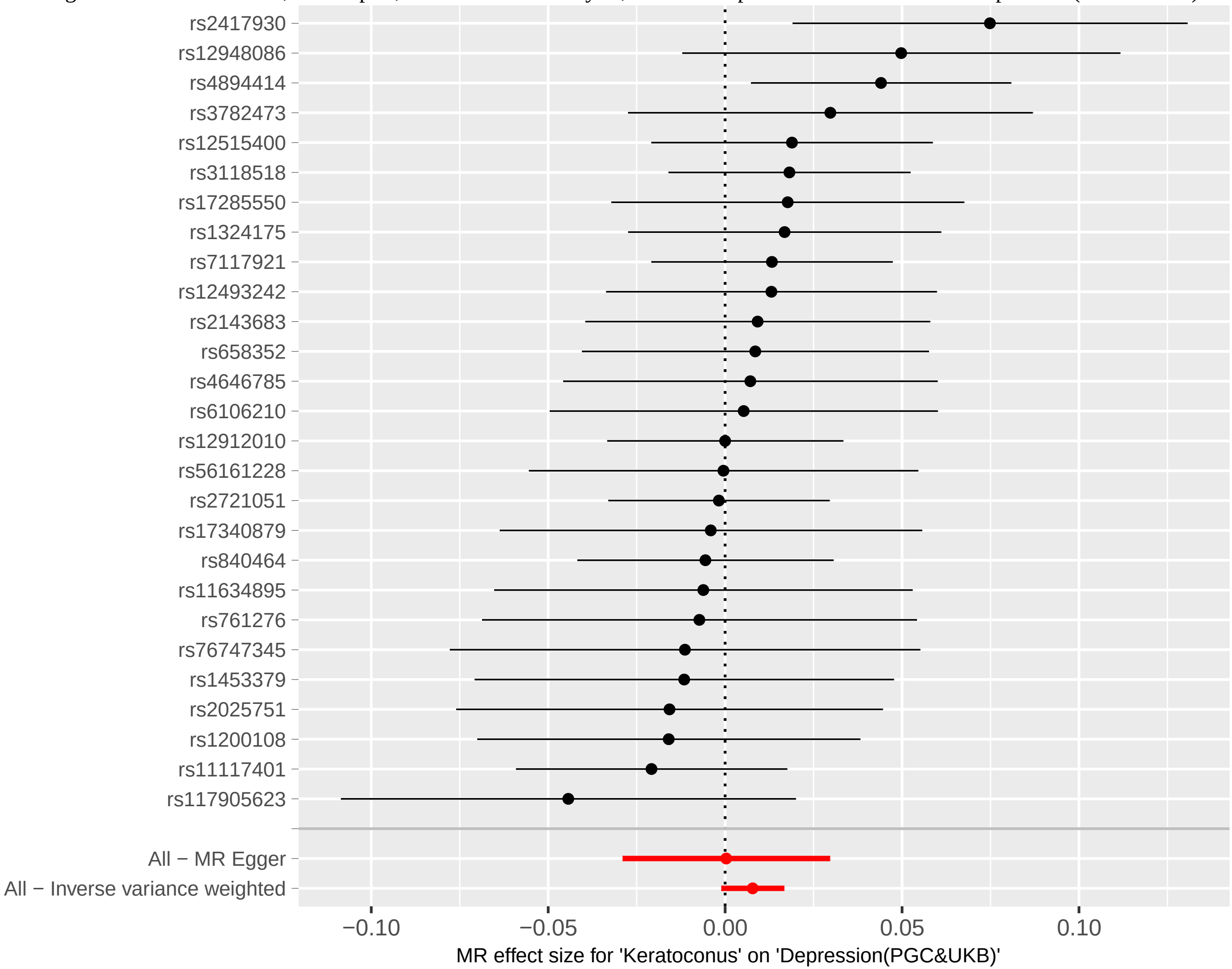

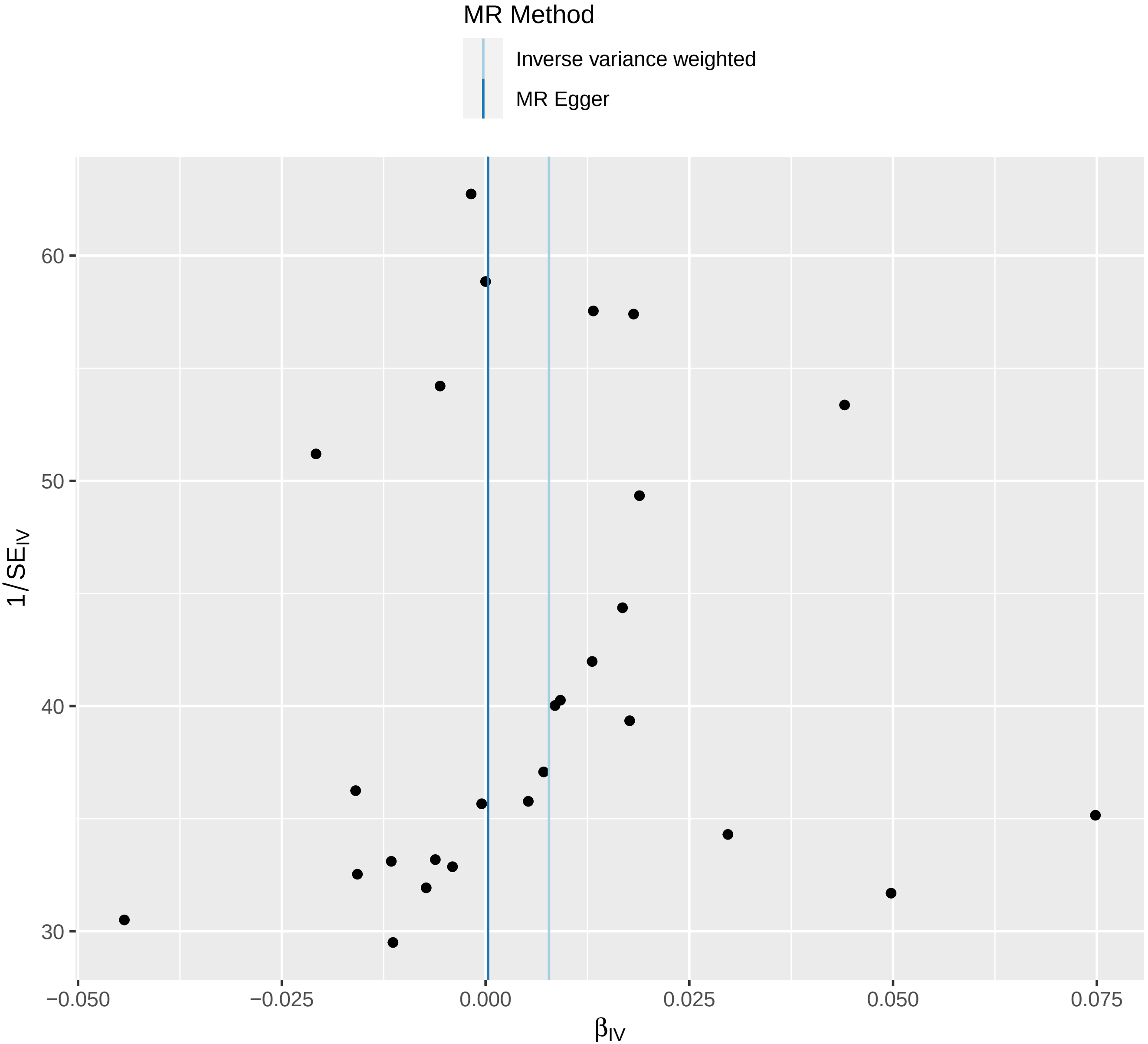
MR Method
Inverse variance weighted
MR Egger
60
50
40
30
1/SE_IV
−0.050
−0.025
0.000
0.025
0.050
0.075
β_IV

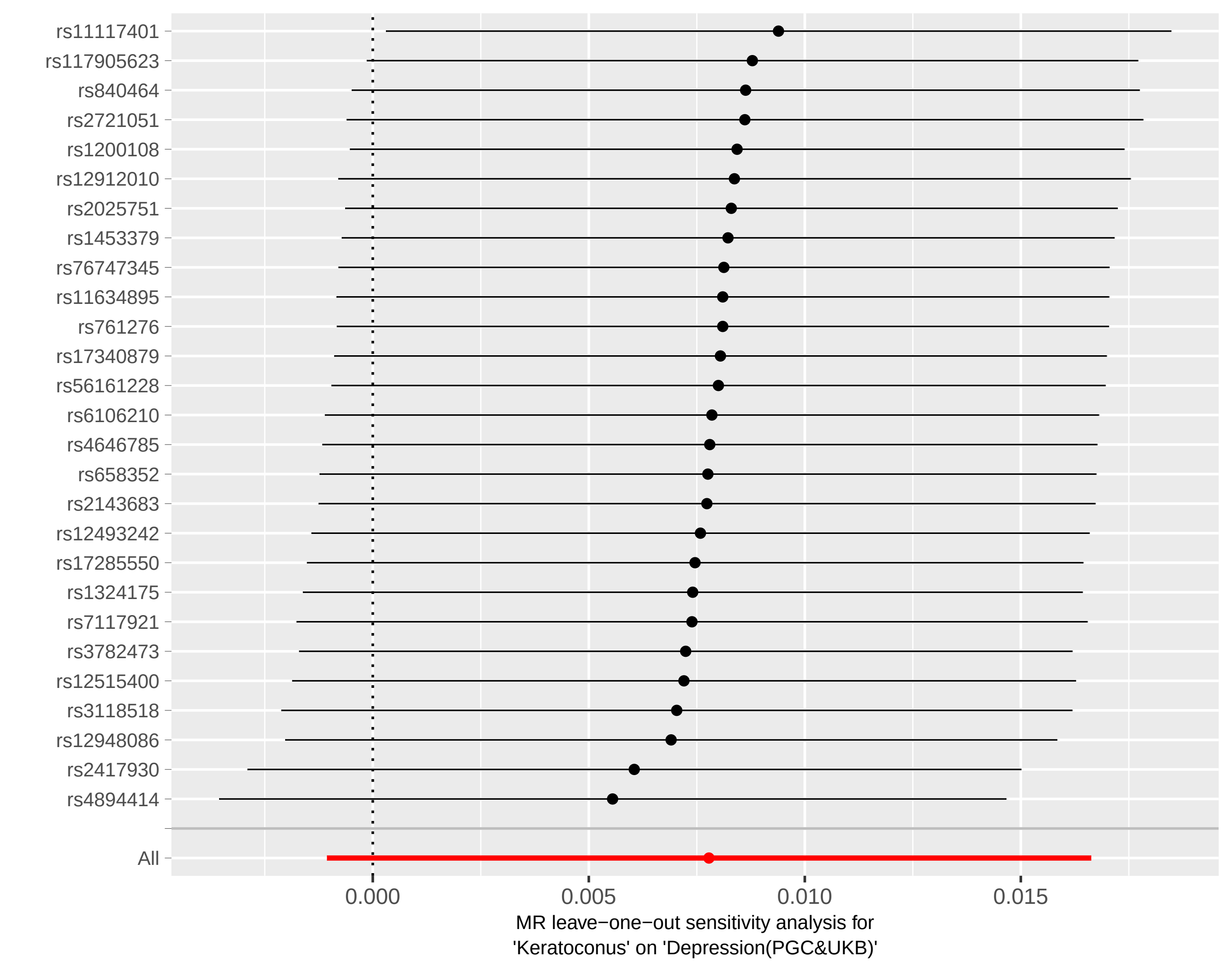

rs11117401
rs117905623
rs840464
rs2721051
rs1200108
rs12912010
rs2025751
rs1453379
rs76747345
rs11634895
rs761276
rs17340879
rs56161228
rs6106210
rs4646785
rs658352
rs2143683
rs12493242
rs17285550
rs1324175
rs7117921
rs3782473
rs12515400
rs3118518
rs12948086
rs2417930
rs4894414
All
0.000
0.005
0.010
0.015
MR leave−one−out sensitivity analysis for
'Keratoconus' on 'Depression(PGC&UKB)'

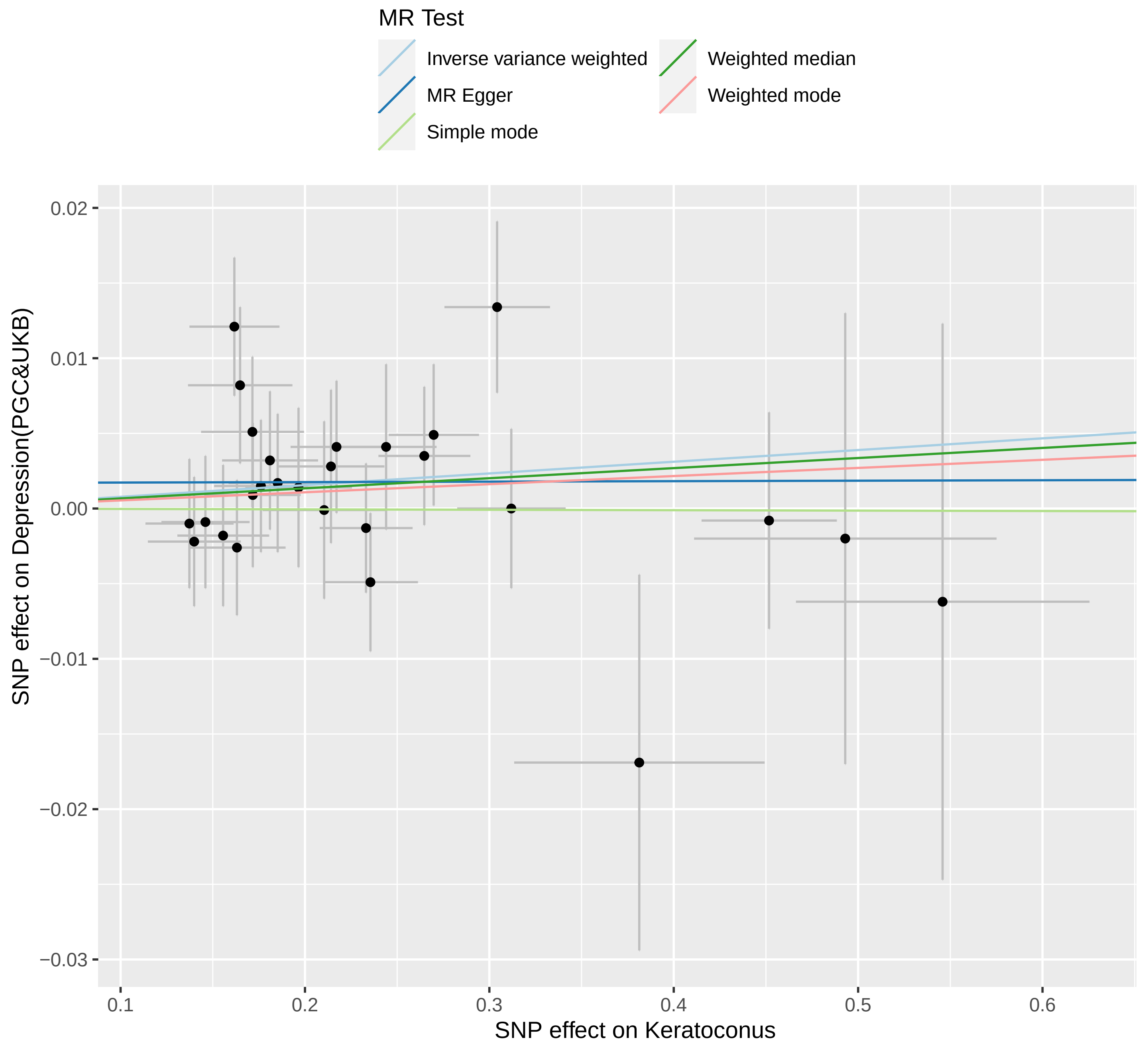
MR Test
Inverse variance weighted
MR Egger
Simple mode
Weighted median
Weighted mode
SNP effect on Depression(PGC&UKB)
0.02
0.01
0.00
−0.01
−0.02
−0.03
0.1
0.2
0.3
0.4
0.5
0.6
SNP effect on Keratoconus

Figure S4. MR effect size, funnel plot, Leave-one-out analysis, and scatter plot for Keratoconus on Depression(FinnGen).

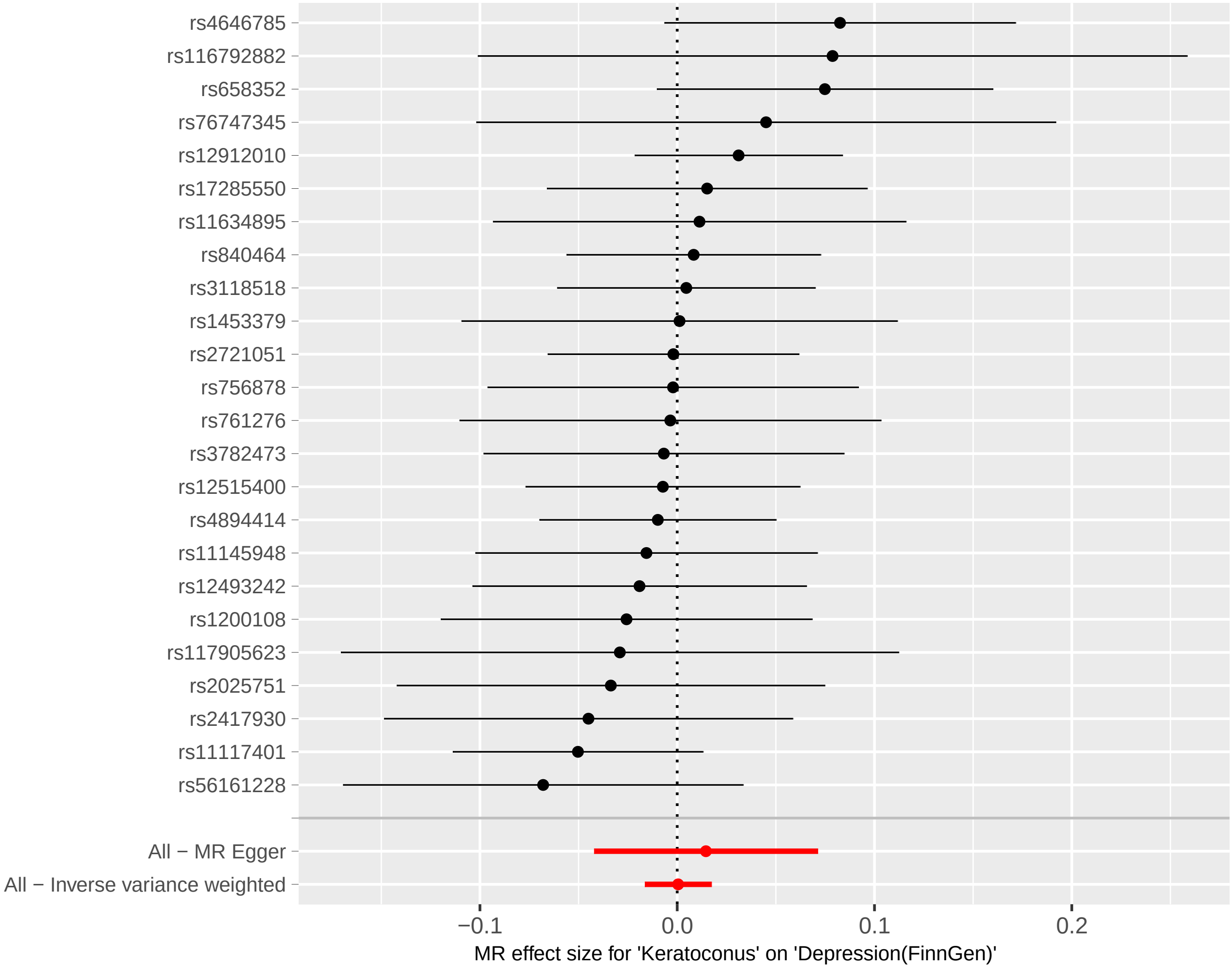

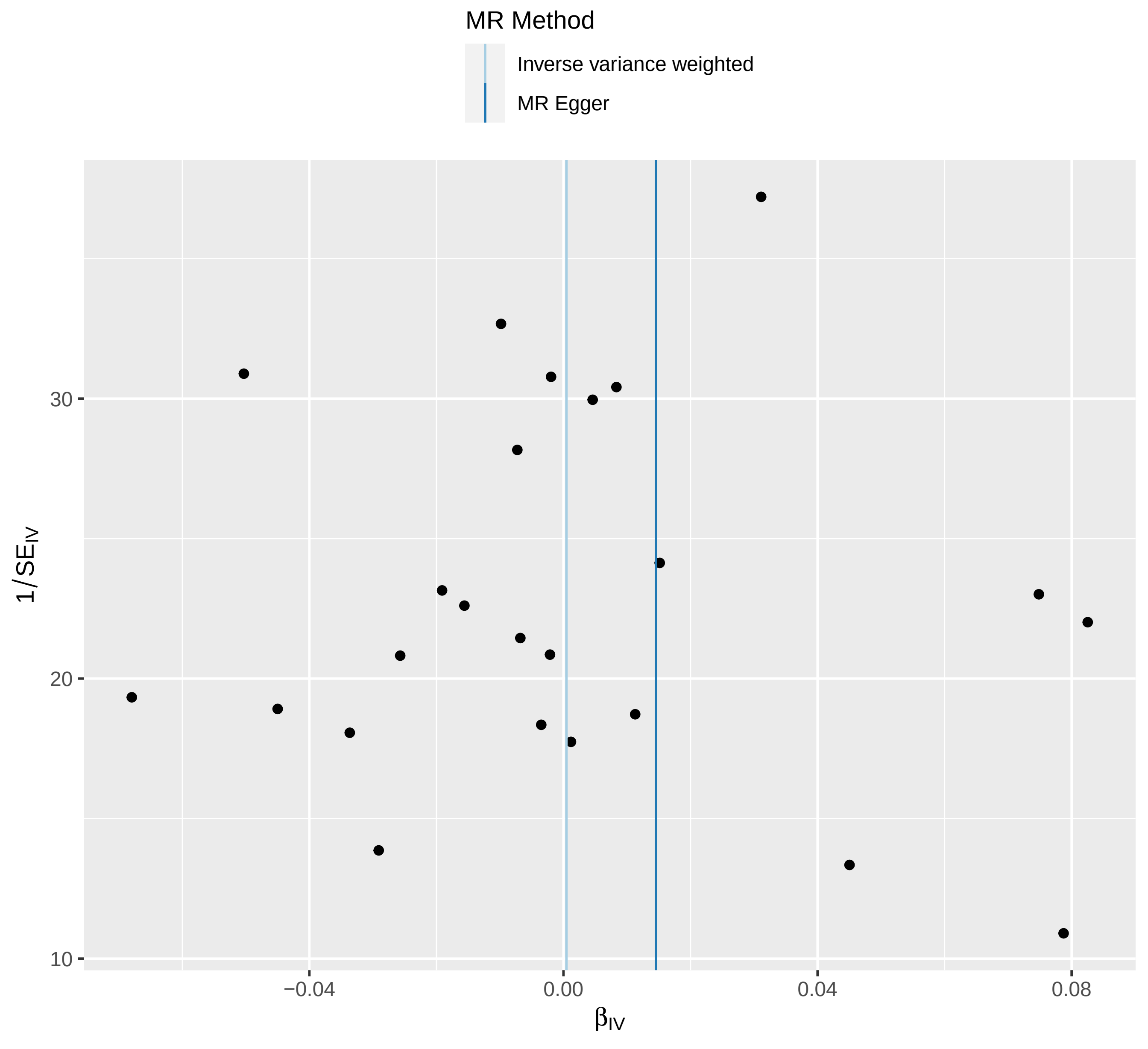
MR Method
Inverse variance weighted
MR Egger
30
20
10
−0.04
0.00
0.04
0.08
$1/SE_{IV}$
β_{IV}

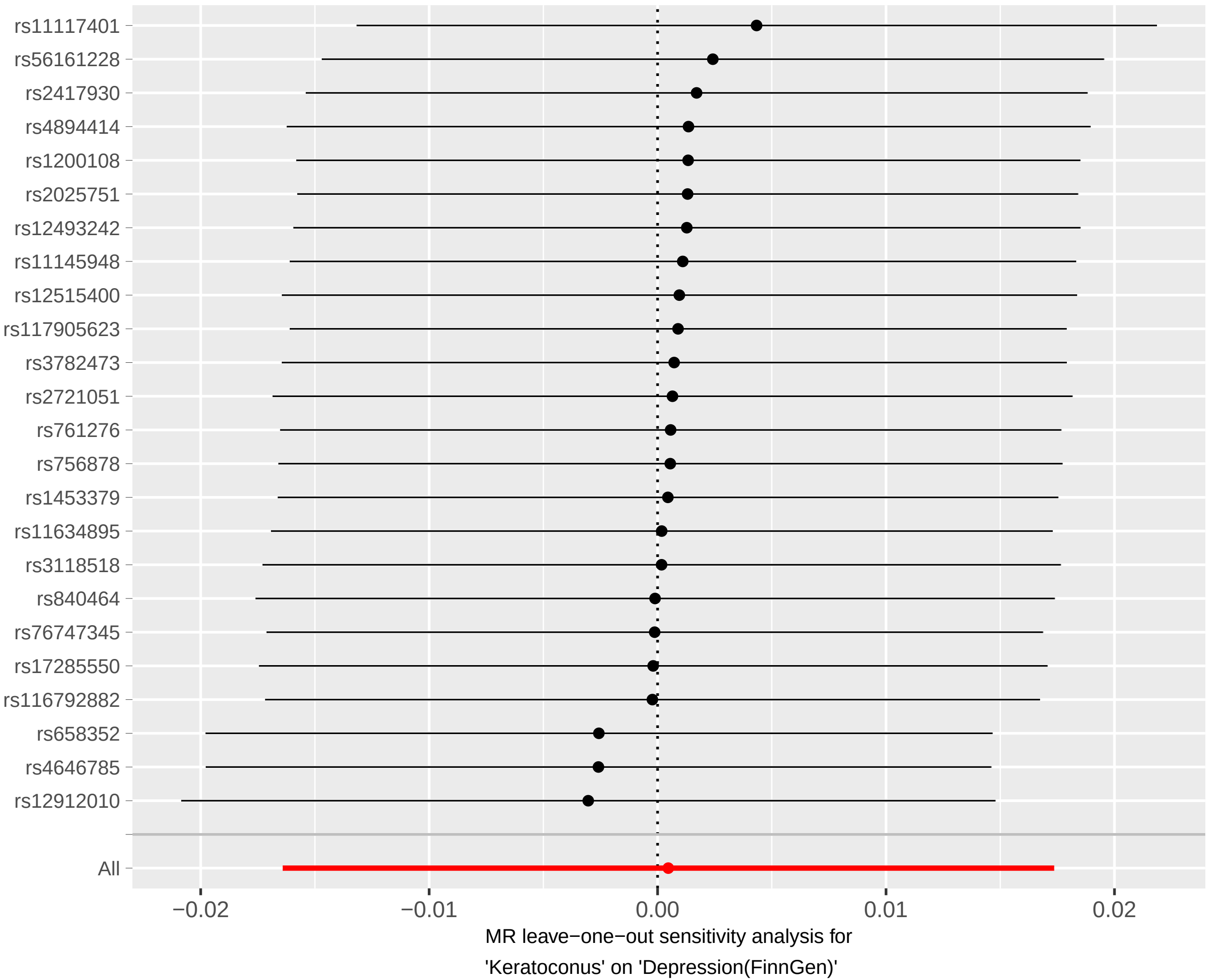
rs11117401
rs56161228
rs2417930
rs4894414
rs1200108
rs2025751
rs12493242
rs11145948
rs12515400
rs117905623
rs3782473
rs2721051
rs761276
rs756878
rs1453379
rs11634895
rs3118518
rs840464
rs76747345
rs17285550
rs116792882
rs658352
rs4646785
rs12912010
All
−0.02
−0.01
0.00
0.01
0.02
MR leave−one−out sensitivity analysis for
'Keratoconus' on 'Depression(FinnGen)'

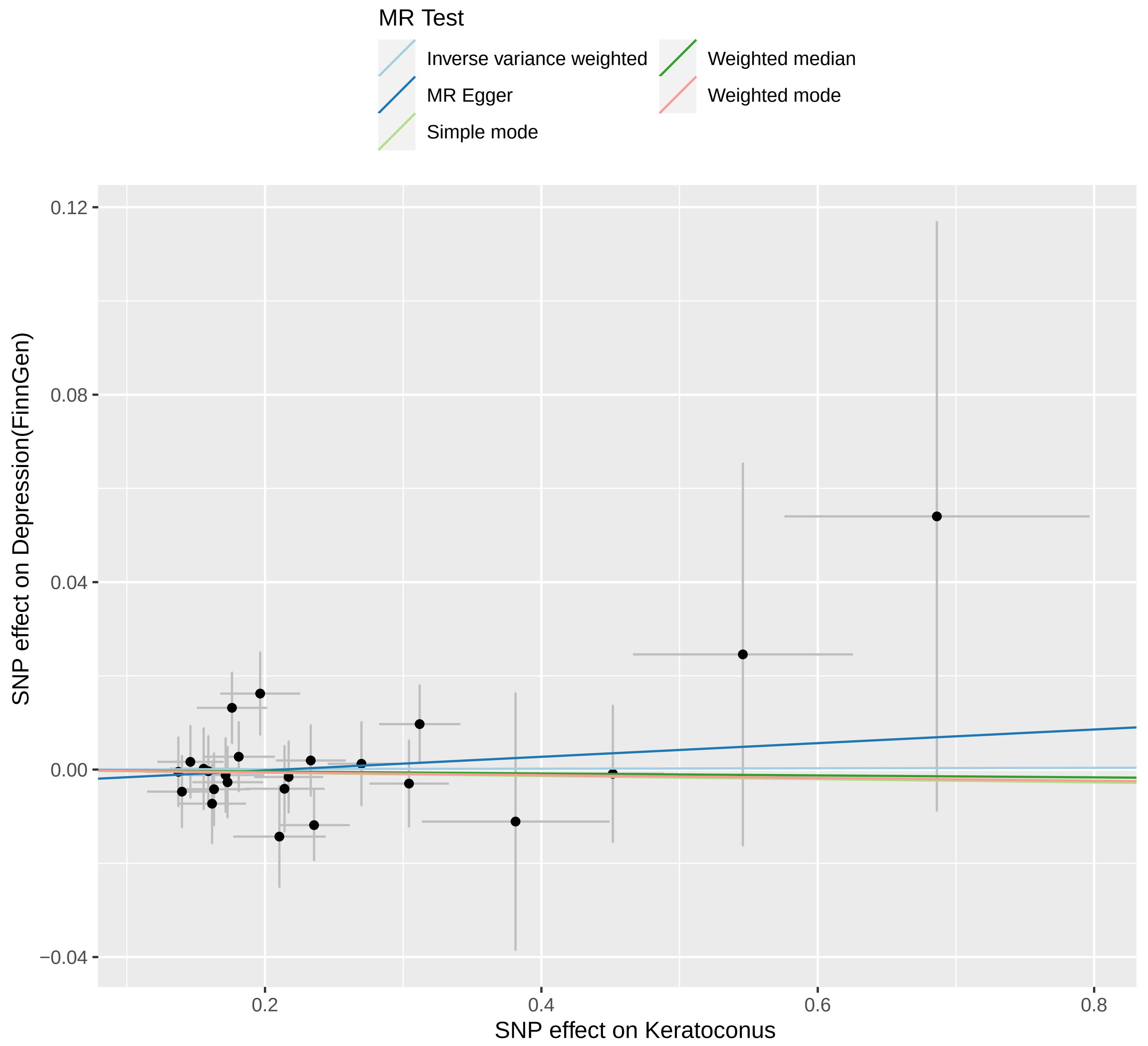
MR Test
Inverse variance weighted
MR Egger
Simple mode
Weighted median
Weighted mode
0.12
0.08
0.04
0.00
−0.04
0.2
0.4
0.6
0.8
SNP effect on Depression(FinnGen)
SNP effect on Keratoconus

Figure S5. MR effect size, funnel plot, Leave-one-out analysis, and scatter plot for Keratoconus on Schizophrenia(PGC).

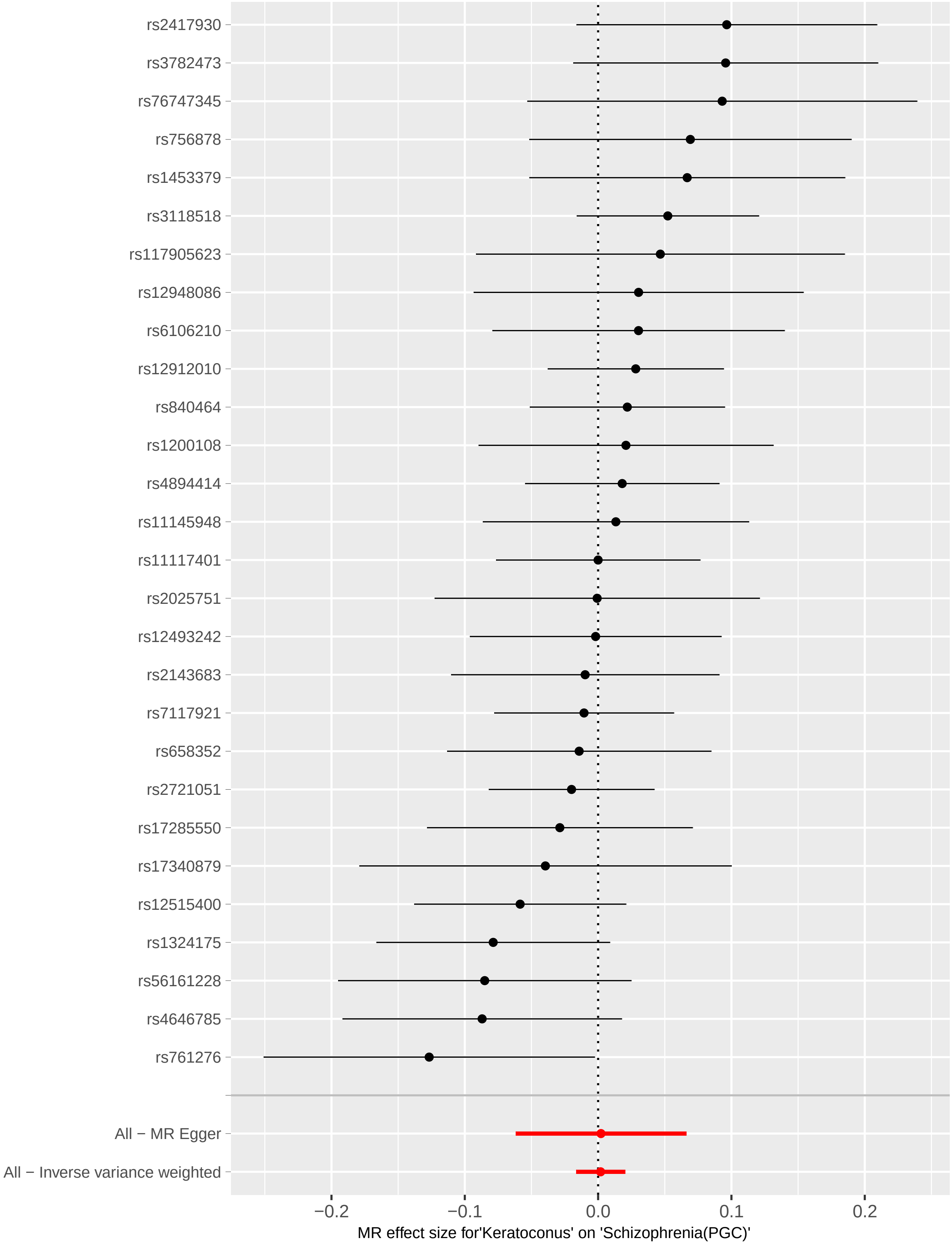

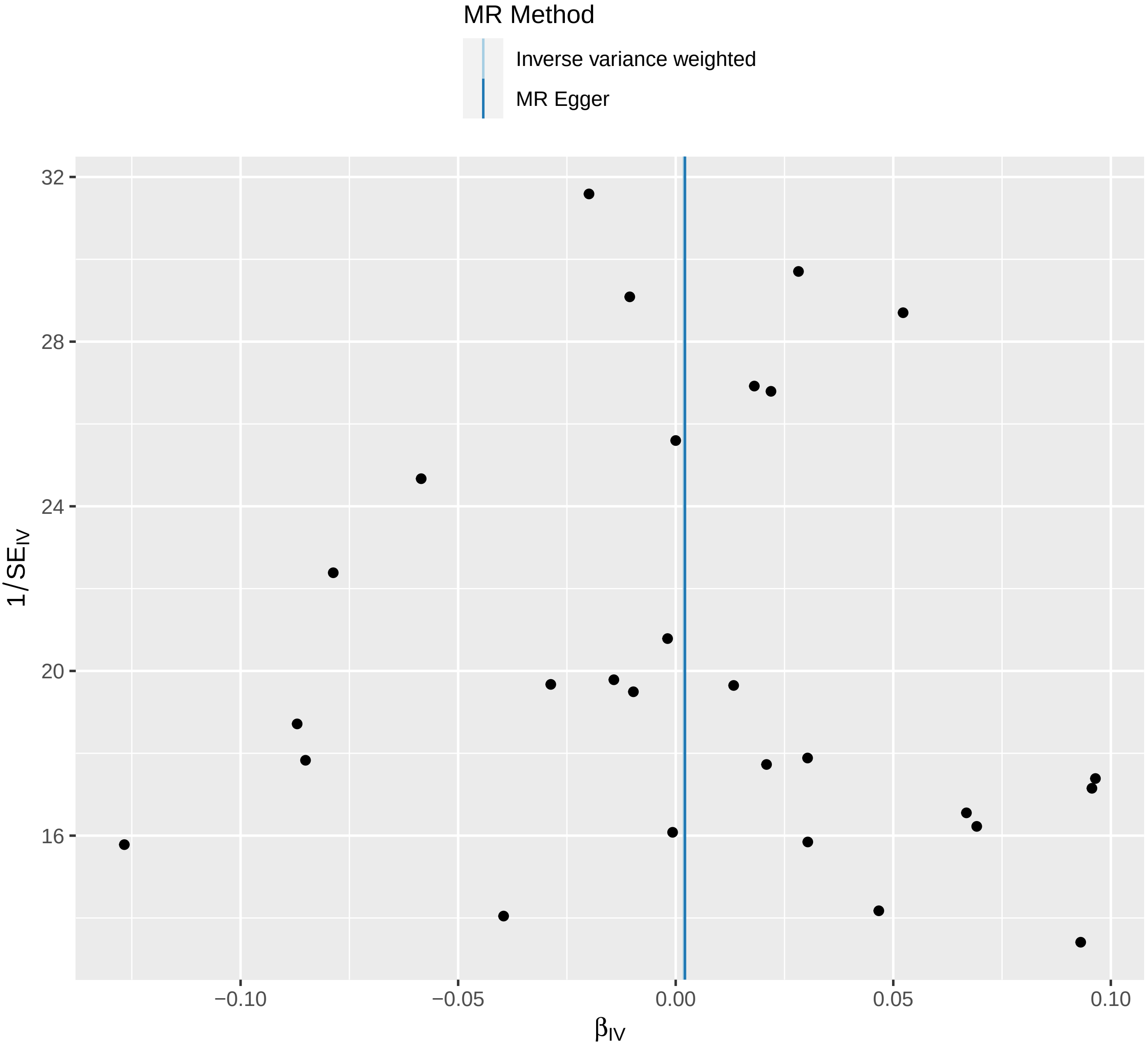
MR Method
Inverse variance weighted
MR Egger
32
28
24
20
16
$1/SE_{IV}$
−0.10
−0.05
0.00
0.05
0.10
β_{IV}

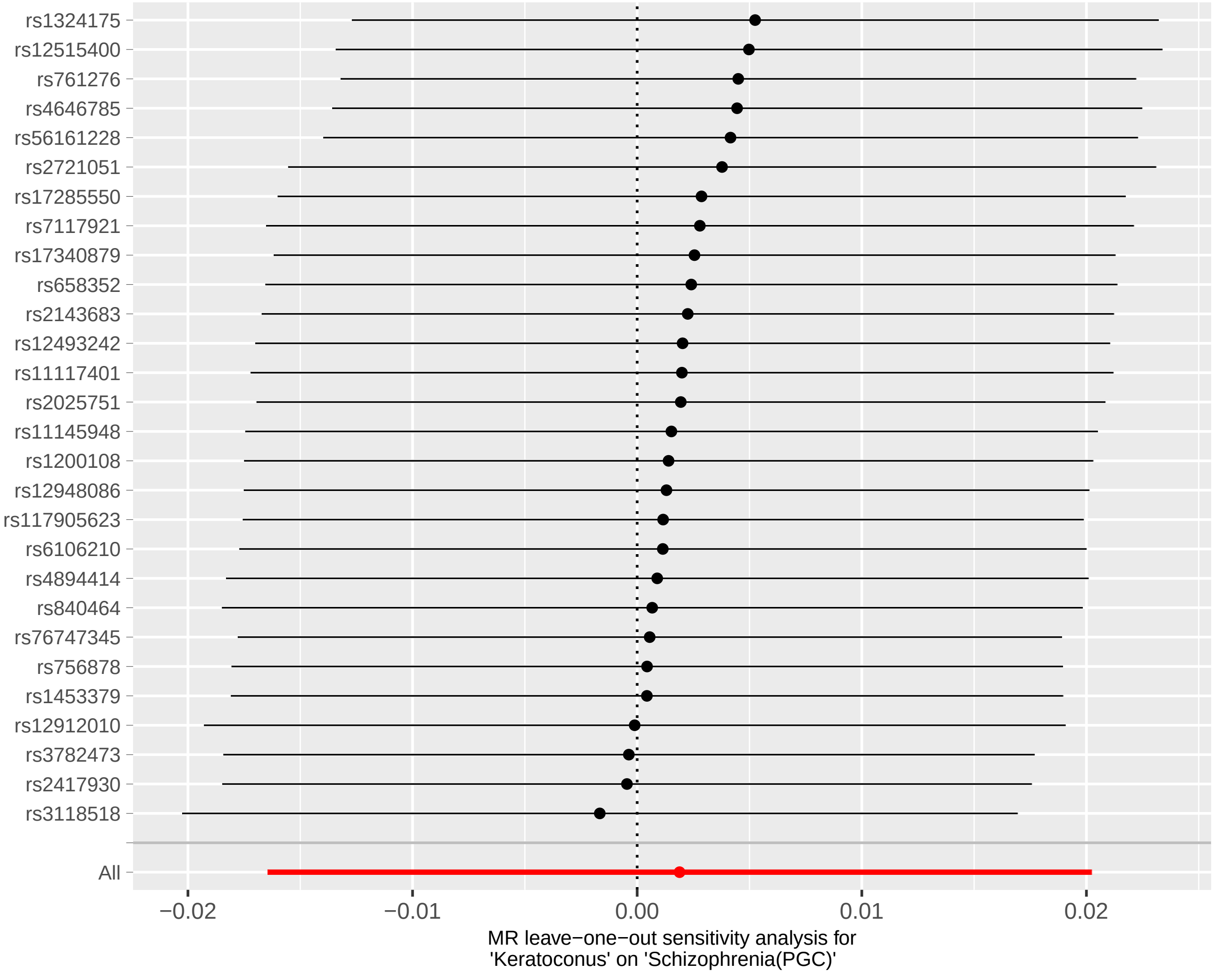

rs1324175
rs12515400
rs761276
rs4646785
rs56161228
rs2721051
rs17285550
rs7117921
rs17340879
rs658352
rs2143683
rs12493242
rs11117401
rs2025751
rs11145948
rs1200108
rs12948086
rs117905623
rs6106210
rs4894414
rs840464
rs76747345
rs756878
rs1453379
rs12912010
rs3782473
rs2417930
rs3118518
All
−0.02
−0.01
0.00
0.01
0.02
MR leave−one−out sensitivity analysis for
'Keratoconus' on 'Schizophrenia(PGC)'

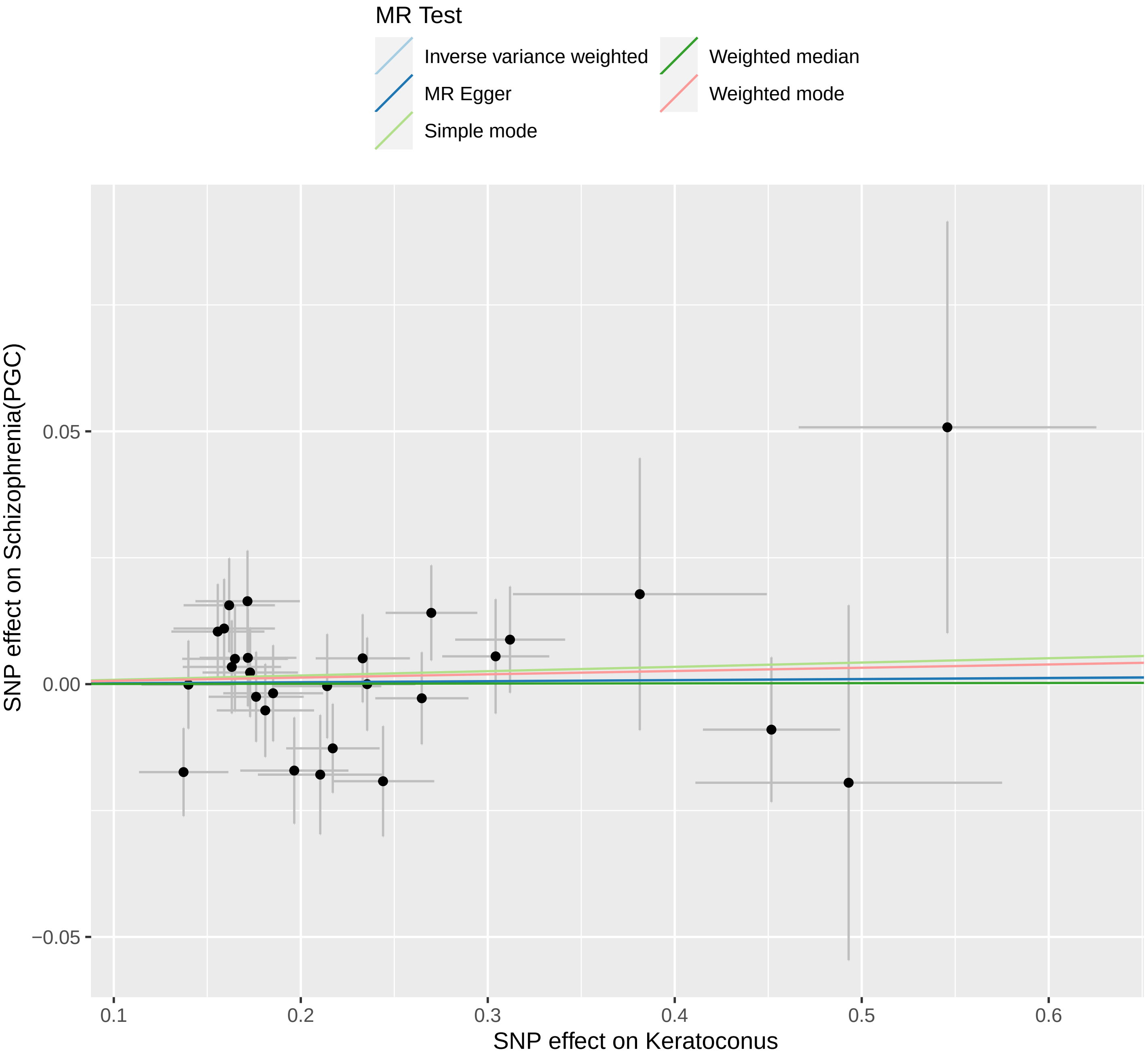

MR Test
Inverse variance weighted
MR Egger
Simple mode
Weighted median
Weighted mode
SNP effect on Schizophrenia(PGC)
0.05
0.00
−0.05
0.1
0.2
0.3
0.4
0.5
0.6
SNP effect on Keratoconus

Figure S6. MR effect size, funnel plot, Leave-one-out analysis, and scatter plot for Keratoconus on Schizophrenia(FinnGen).

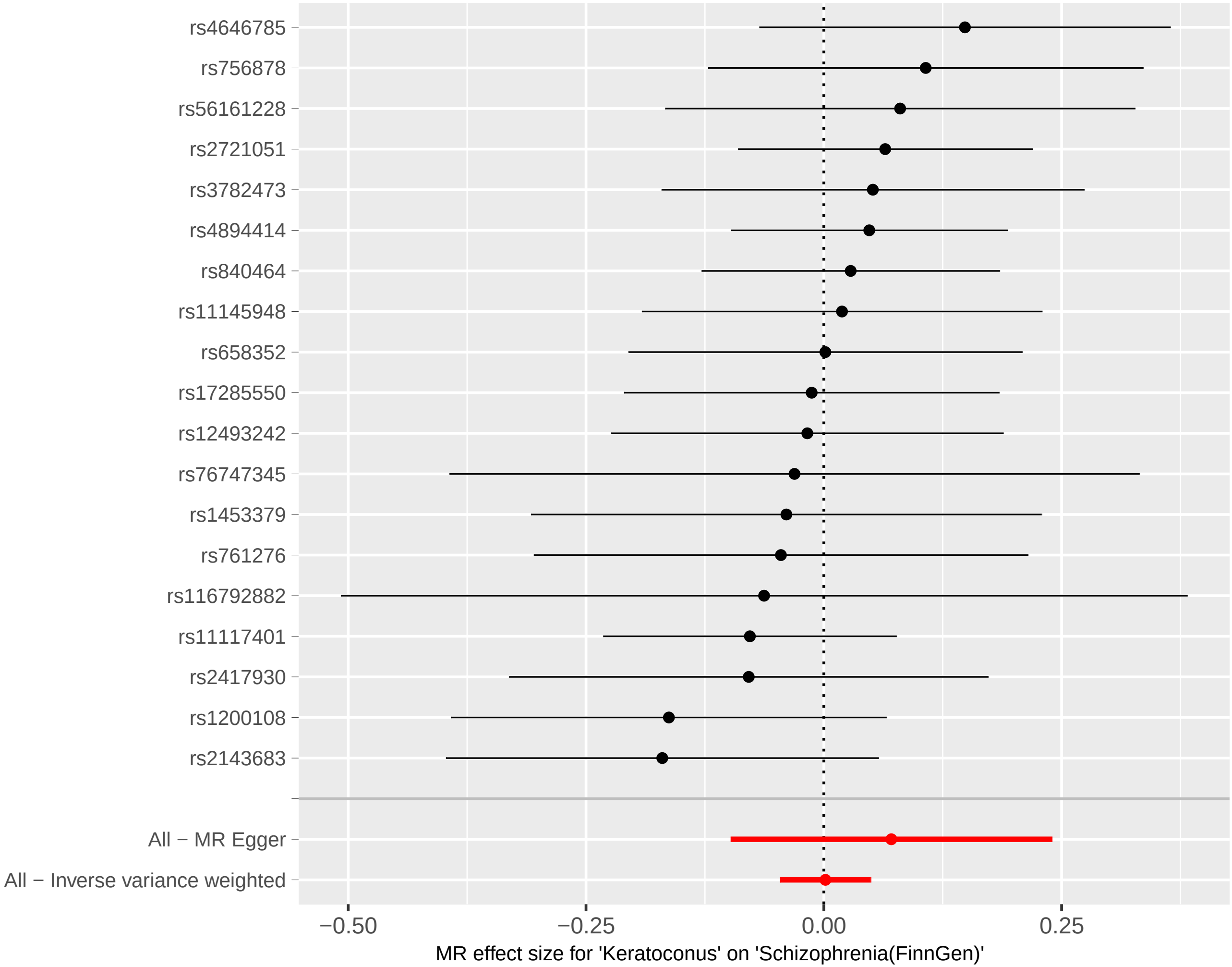

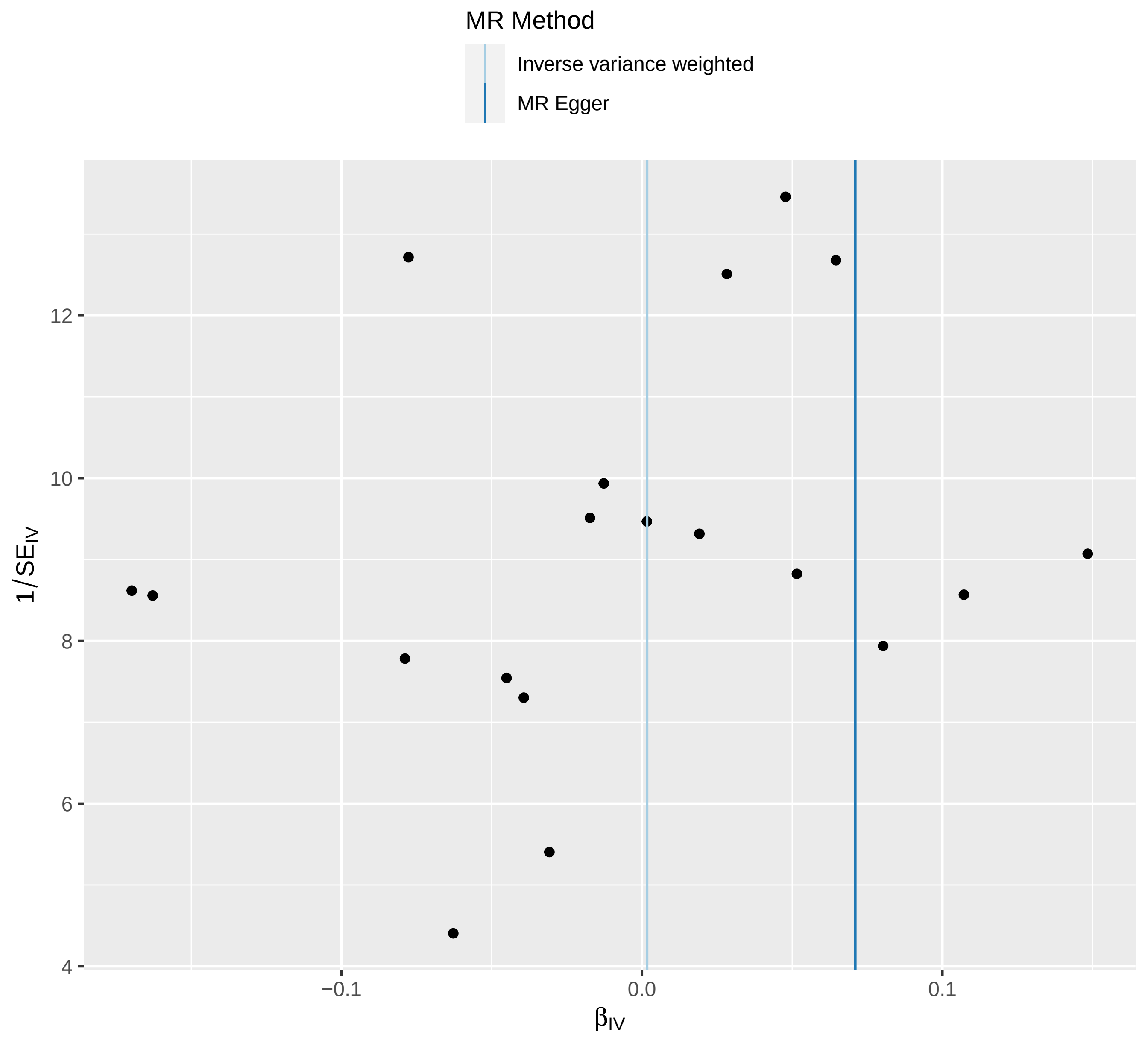
MR Method
Inverse variance weighted
MR Egger
1/SE_IV
12
10
8
6
4
−0.1
0.0
0.1
β_IV

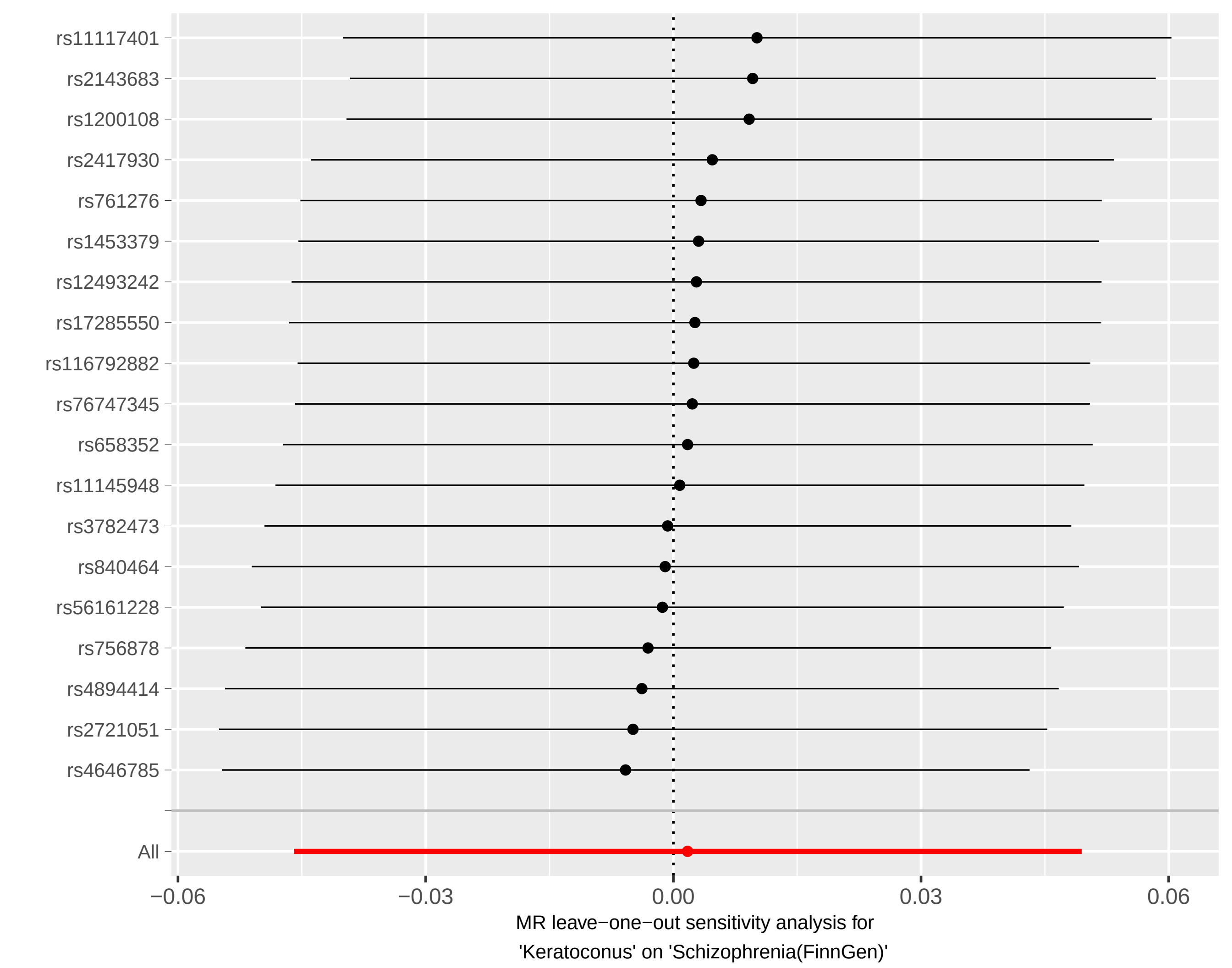

rs11117401
rs2143683
rs1200108
rs2417930
rs761276
rs1453379
rs12493242
rs17285550
rs116792882
rs76747345
rs658352
rs11145948
rs3782473
rs840464
rs56161228
rs756878
rs4894414
rs2721051
rs4646785
All
−0.06
−0.03
0.00
0.03
0.06
MR leave−one−out sensitivity analysis for
'Keratoconus' on 'Schizophrenia(FinnGen)'

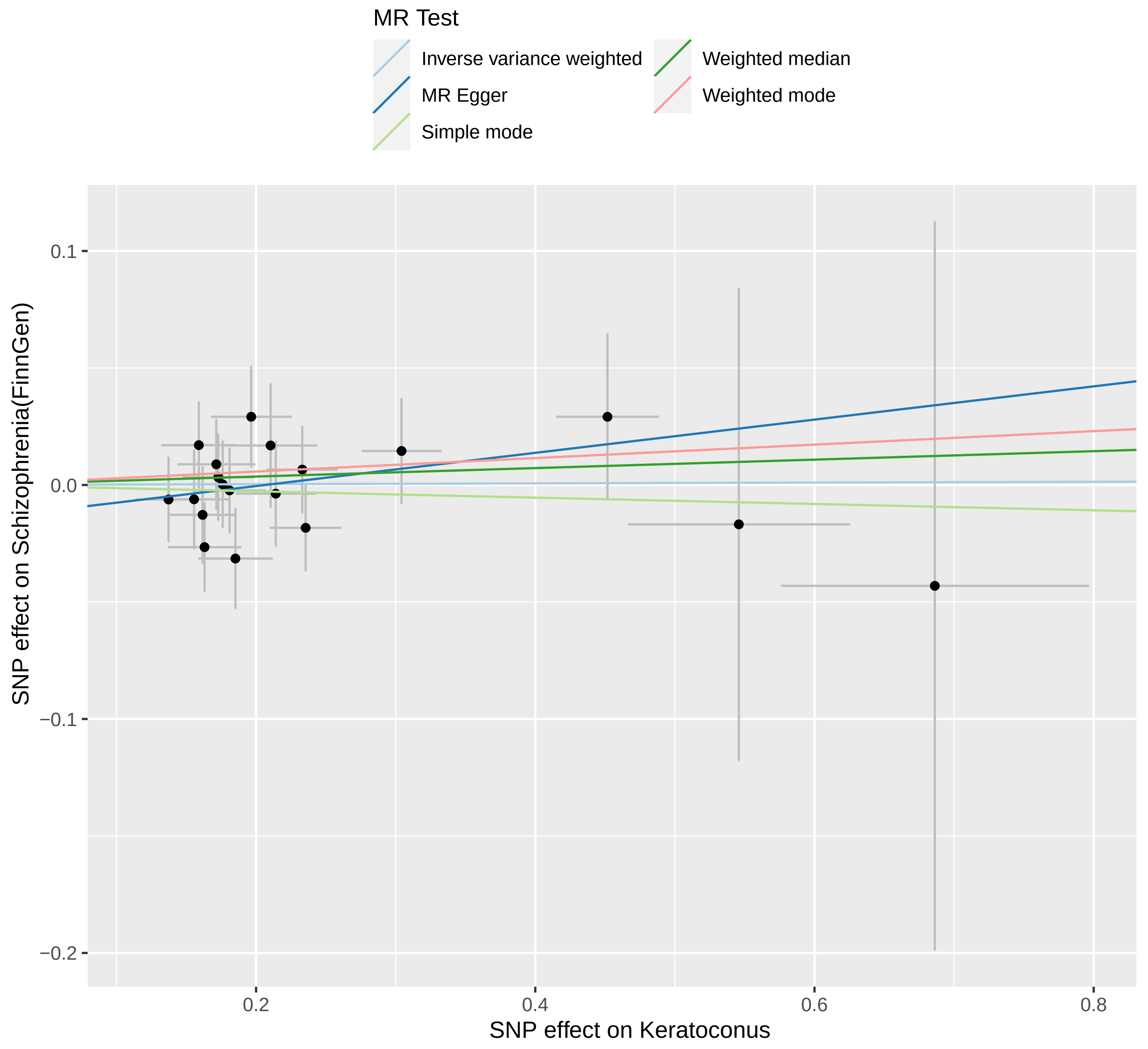
MR Test
Inverse variance weighted
MR Egger
Simple mode
Weighted median
Weighted mode
0.1
0.0
−0.1
−0.2
SNP effect on Schizophrenia(FinnGen)
0.2
0.4
0.6
0.8
SNP effect on Keratoconus
