# Supplementary material for: Mendelian randomization analysis does not reveal a causal influence between keratoconus and three major mental disorders
Source: Front Psychiatry. 2024 Aug 21;15:1370670. doi: 10.3389/fpsyt.2024.1370670 (PMC11371735; doi:10.3389/fpsyt.2024.1370670)
Supplement: Supplementary file 2 [file DataSheet2.pdf]

Figure S1. MR effect size, funnel plot, Leave-one-out analysis, and scatter plot for Anxiety(ANGST) on Keratoconus.

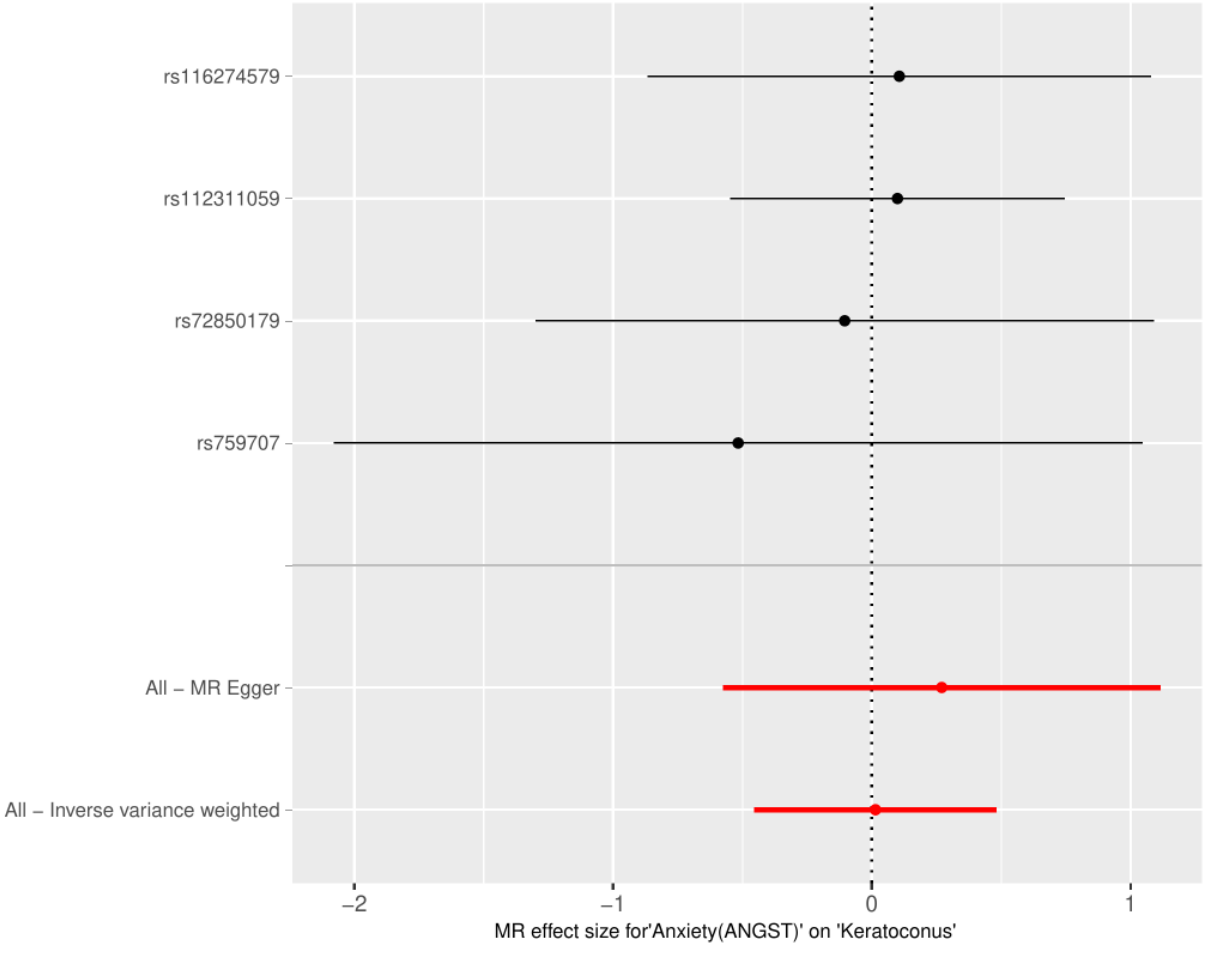

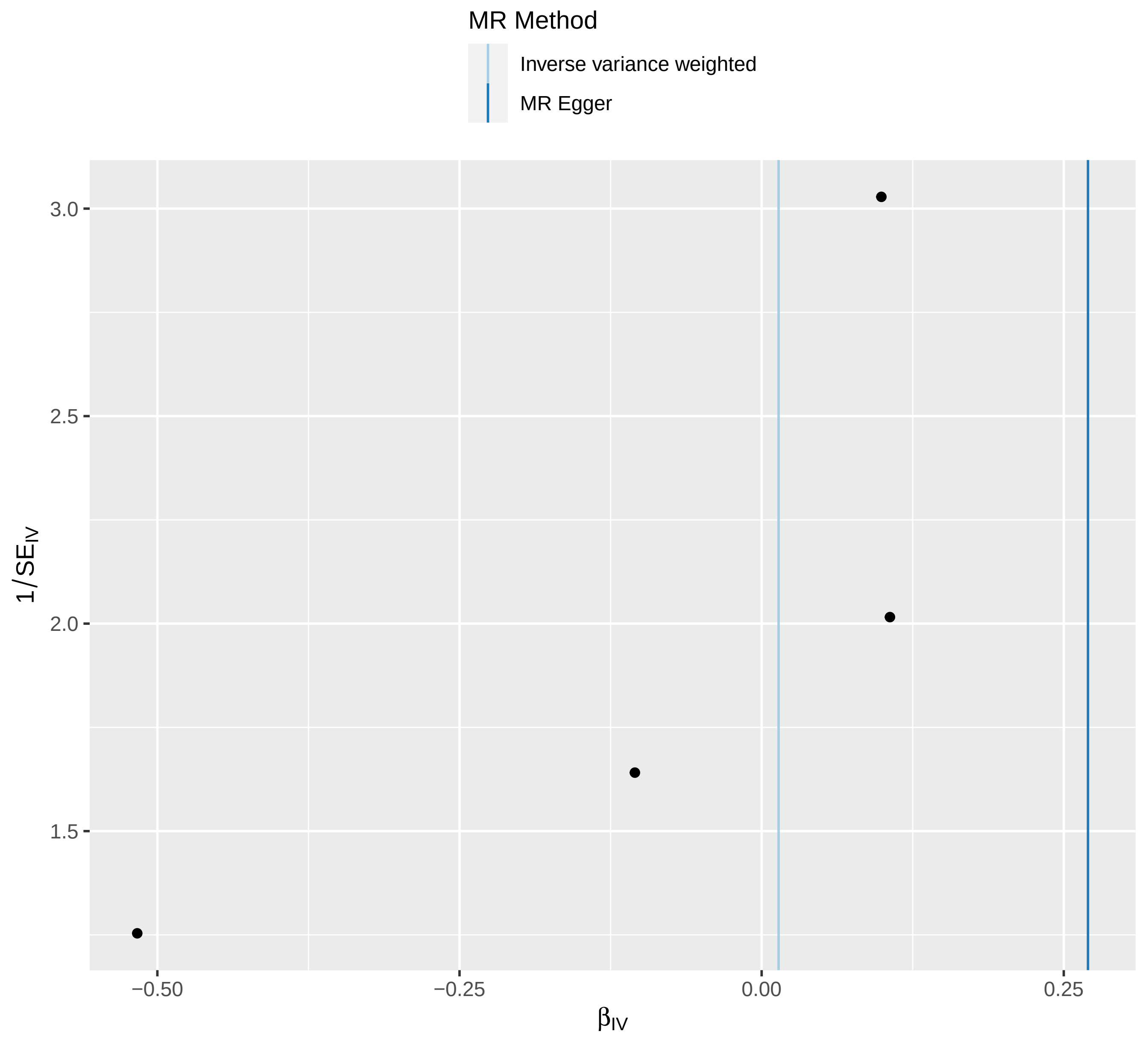
MR Method
Inverse variance weighted
MR Egger
3.0
2.5
2.0
1.5
1/SE_IV
−0.50
−0.25
0.00
0.25
β_IV

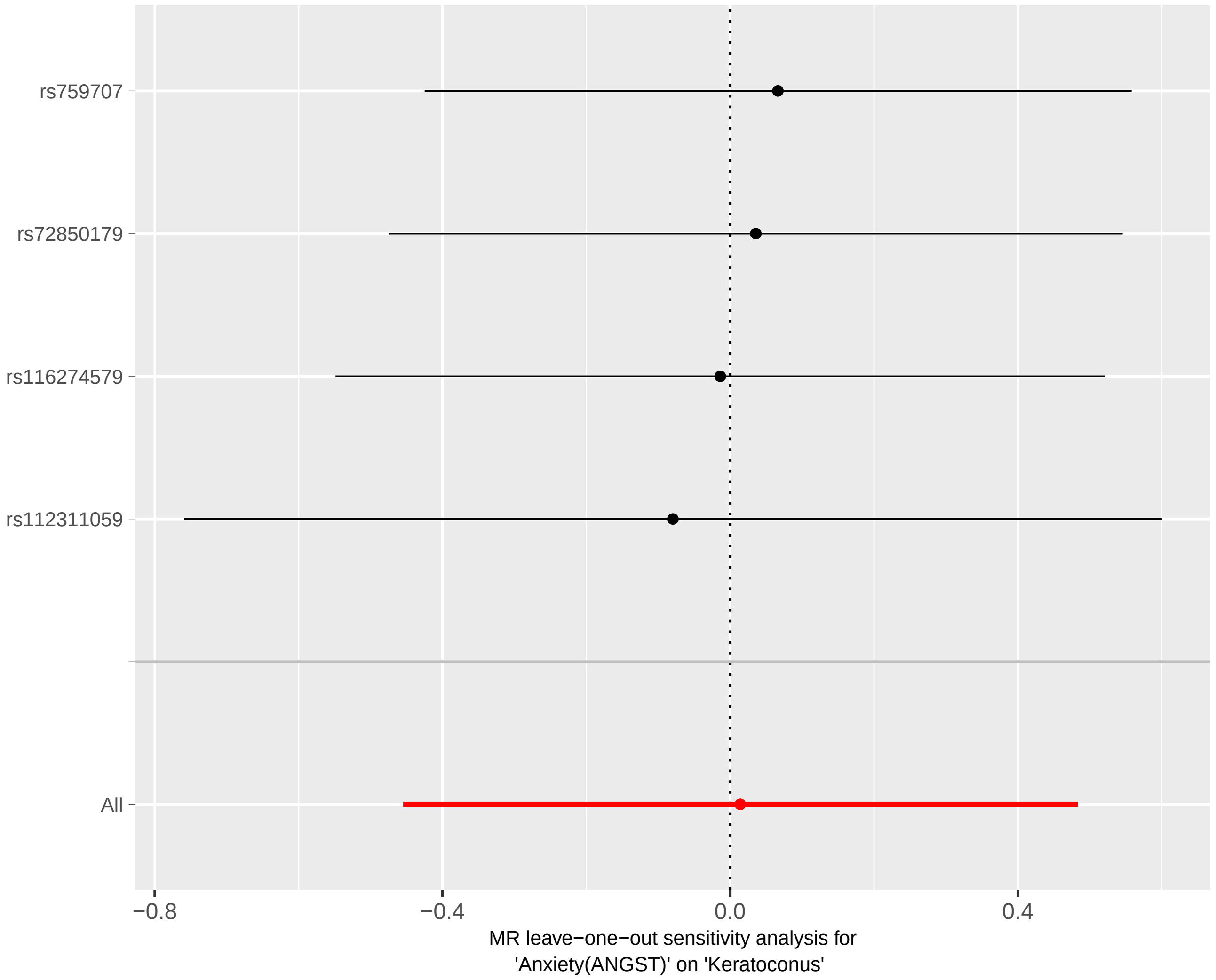

rs759707
rs72850179
rs116274579
rs112311059
All
−0.8
−0.4
0.0
0.4
MR leave−one−out sensitivity analysis for
'Anxiety(ANGST)' on 'Keratoconus'

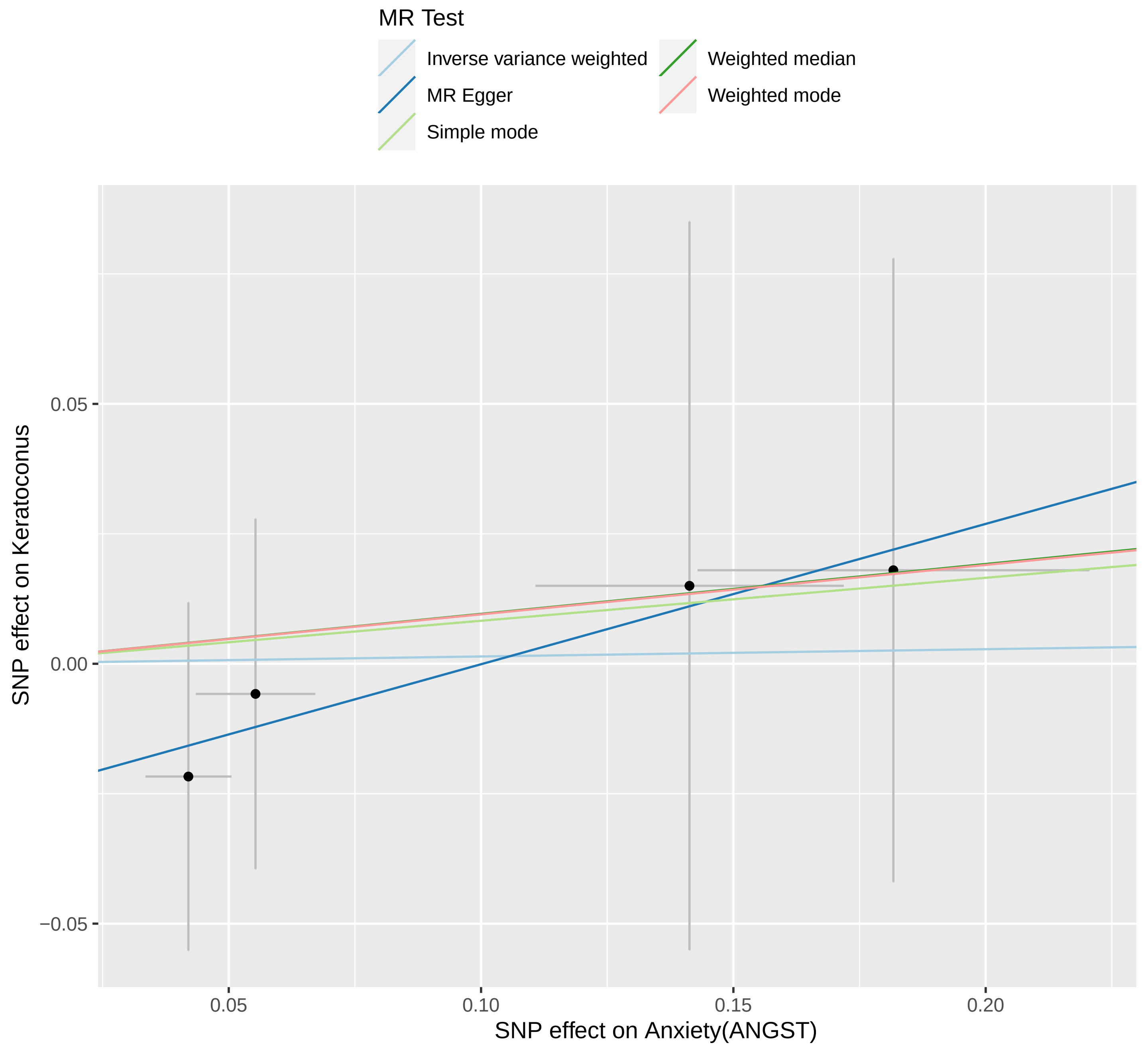
MR Test
Inverse variance weighted
MR Egger
Simple mode
Weighted median
Weighted mode
SNP effect on Keratoconus
0.05
0.00
−0.05
0.05
0.10
0.15
0.20
SNP effect on Anxiety(ANGST)

Figure S2. MR effect size, funnel plot, Leave-one-out analysis, and scatter plot for Anxiety(FinnGen) on Keratoconus.

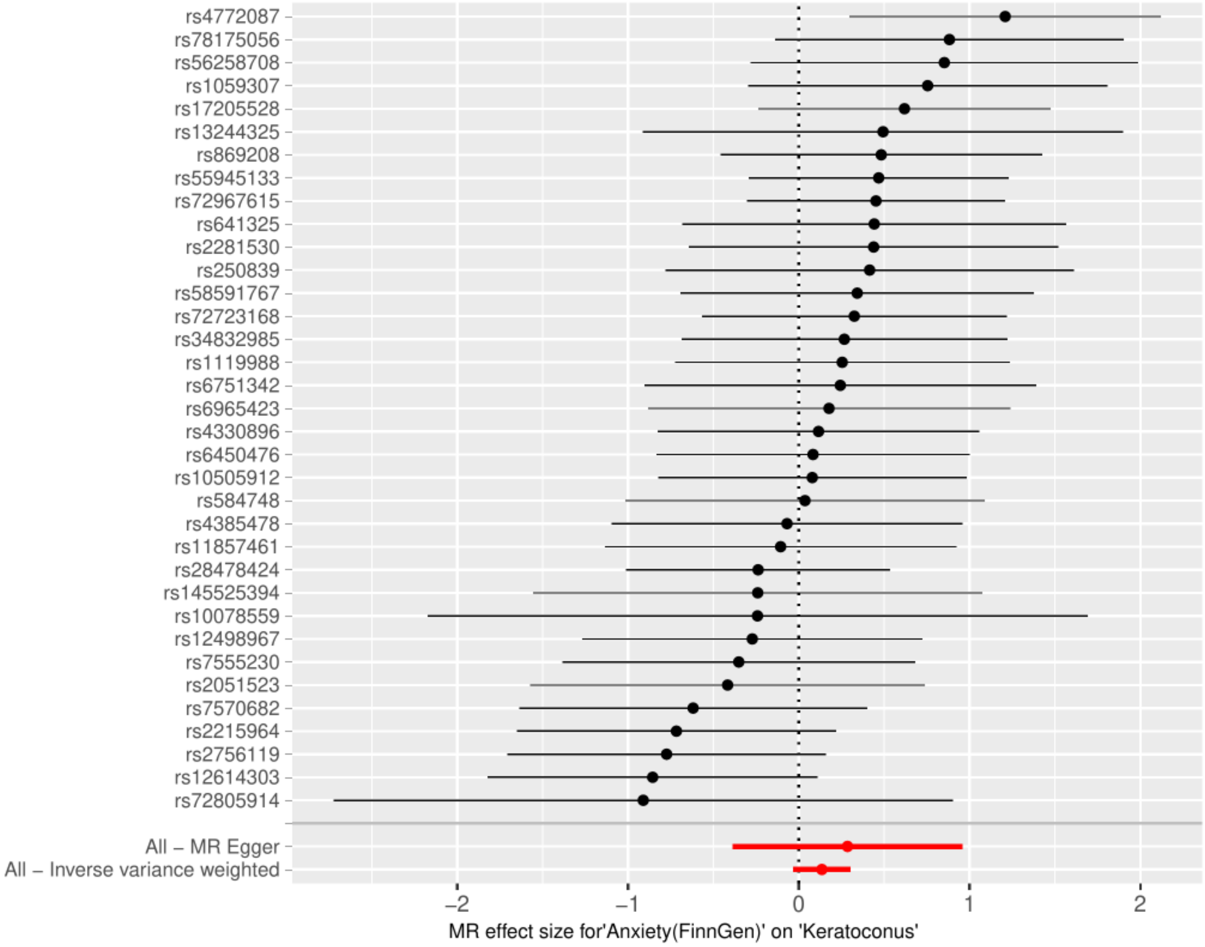

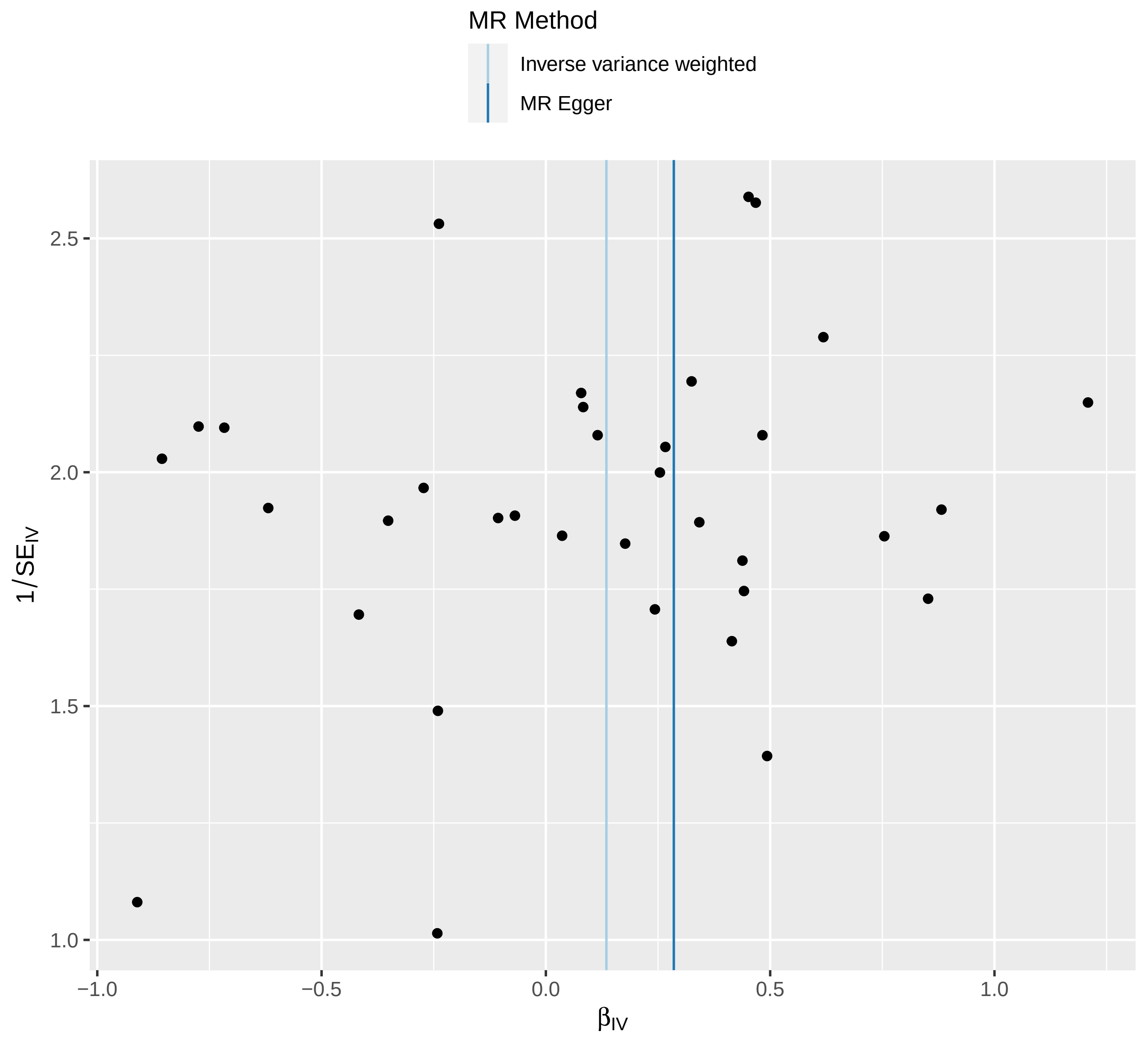

MR Method
Inverse variance weighted
MR Egger
2.5
2.0
1.5
1.0
$1/SE_{IV}$
−1.0
−0.5
0.0
0.5
1.0
β_{IV}

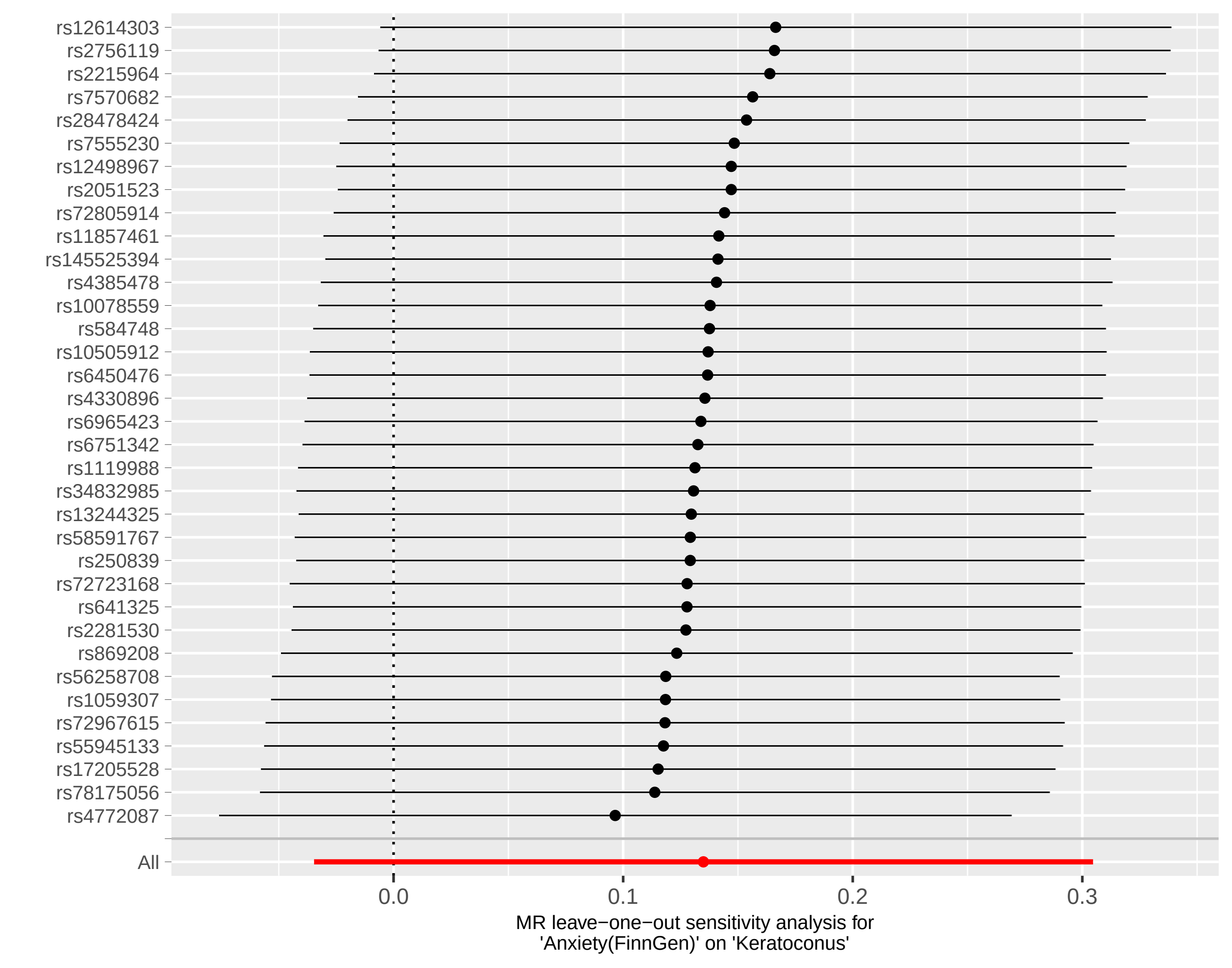
rs12614303
rs2756119
rs2215964
rs7570682
rs28478424
rs7555230
rs12498967
rs2051523
rs72805914
rs11857461
rs145525394
rs4385478
rs10078559
rs584748
rs10505912
rs6450476
rs4330896
rs6965423
rs6751342
rs1119988
rs34832985
rs13244325
rs58591767
rs250839
rs72723168
rs641325
rs2281530
rs869208
rs56258708
rs1059307
rs72967615
rs55945133
rs17205528
rs78175056
rs4772087
All
0.0
0.1
0.2
0.3
MR leave−one−out sensitivity analysis for
'Anxiety(FinnGen)' on 'Keratoconus'

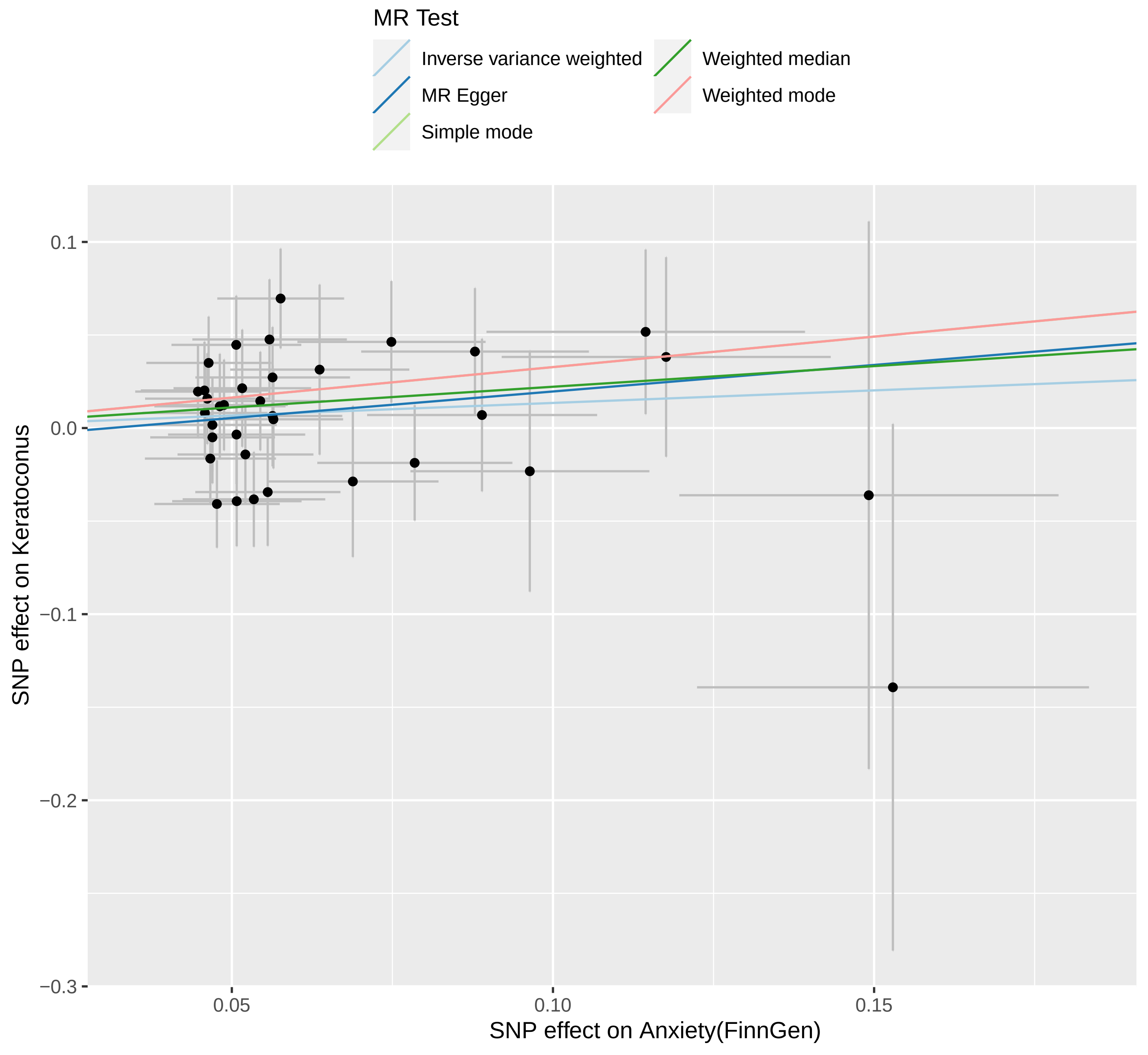
MR Test
Inverse variance weighted
MR Egger
Simple mode
Weighted median
Weighted mode
0.1
0.0
−0.1
−0.2
−0.3
0.05
0.10
0.15
SNP effect on Keratoconus
SNP effect on Anxiety(FinnGen)

Figure S3. MR effect size, funnel plot, Leave-one-out analysis, and scatter plot for Depression(PGC&UKB) on Keratoconus.

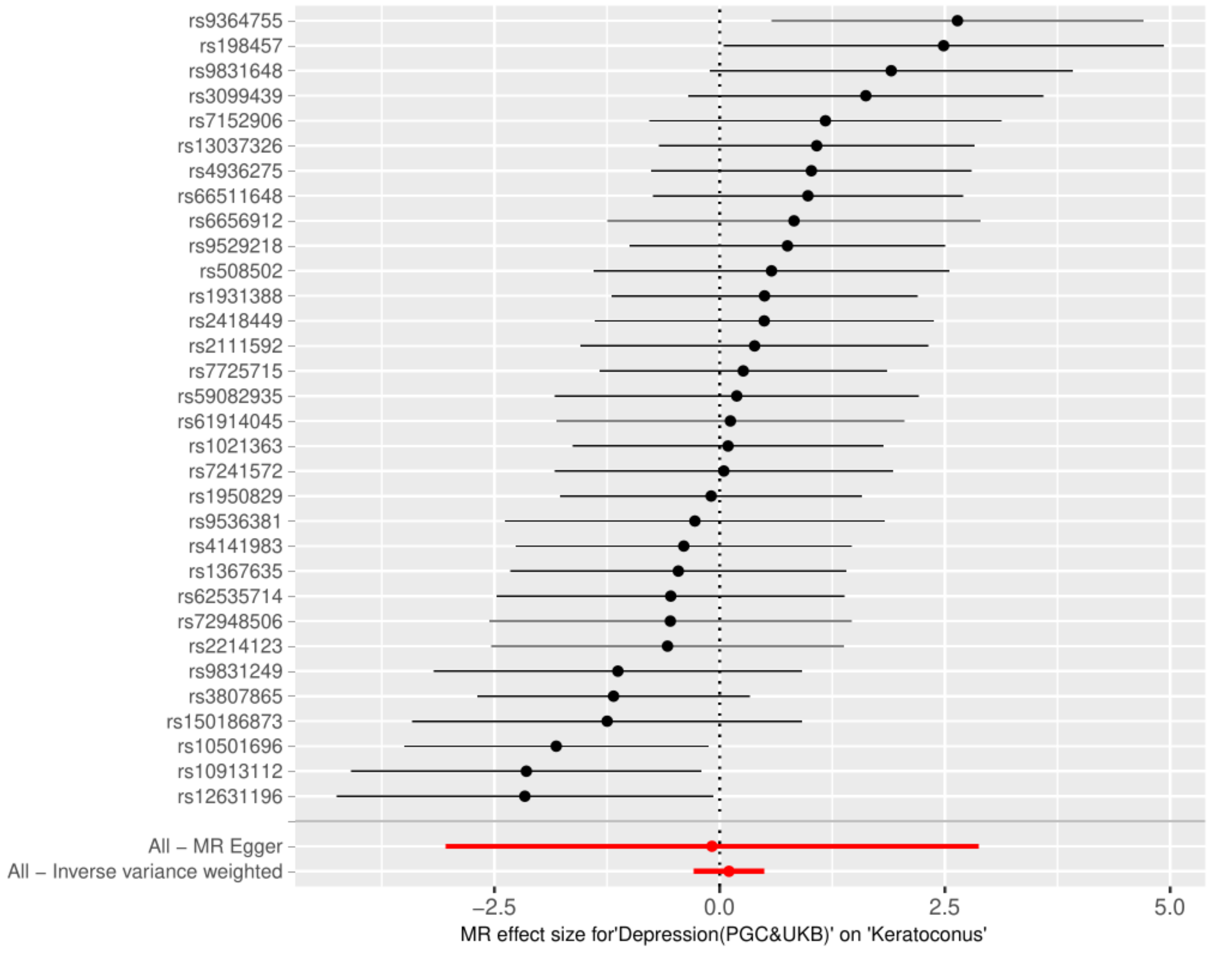

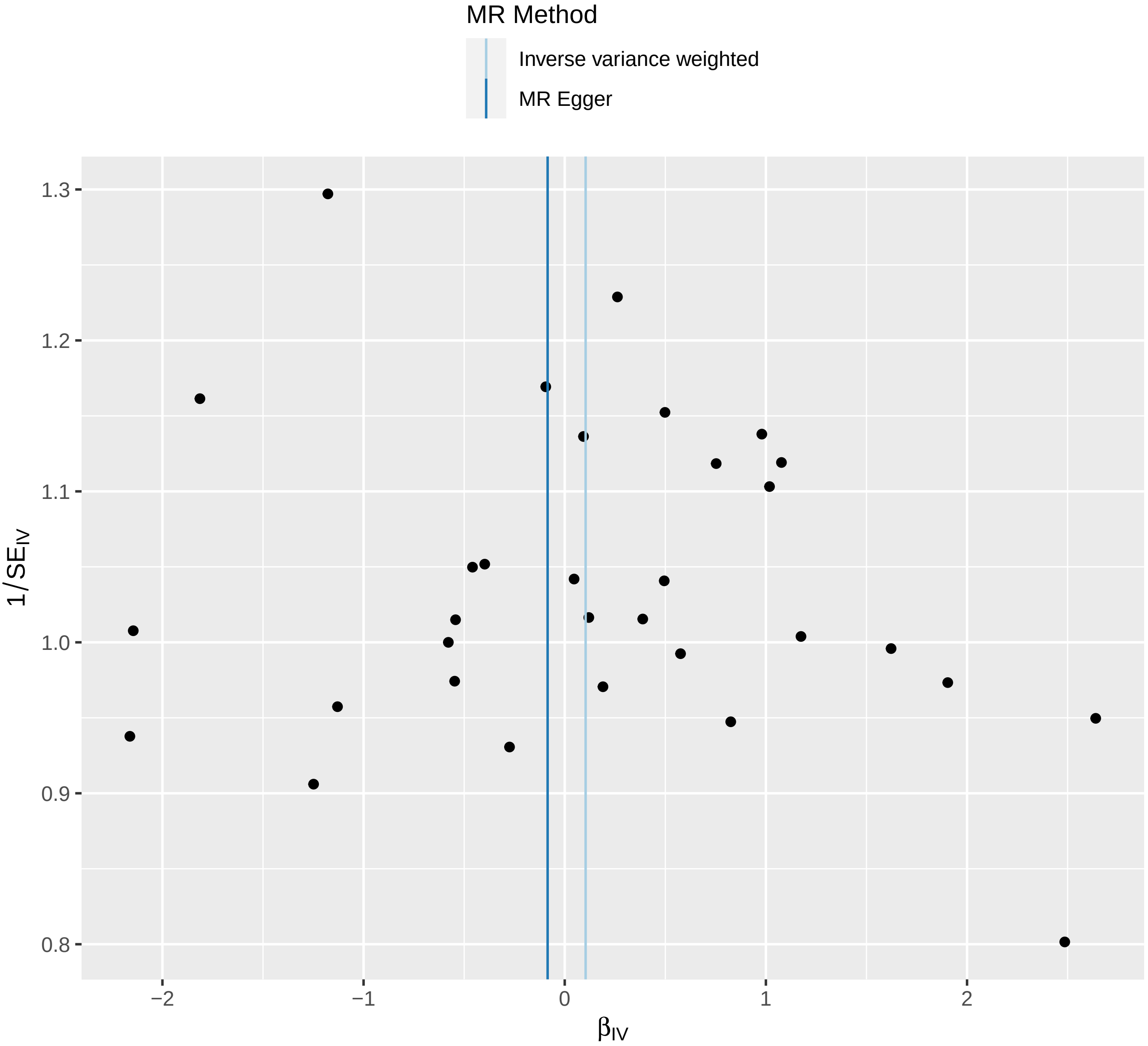

MR Method
Inverse variance weighted
MR Egger
1.3
1.2
1.1
1.0
0.9
0.8
1/SE_IV
−2
−1
0
1
2
β_IV

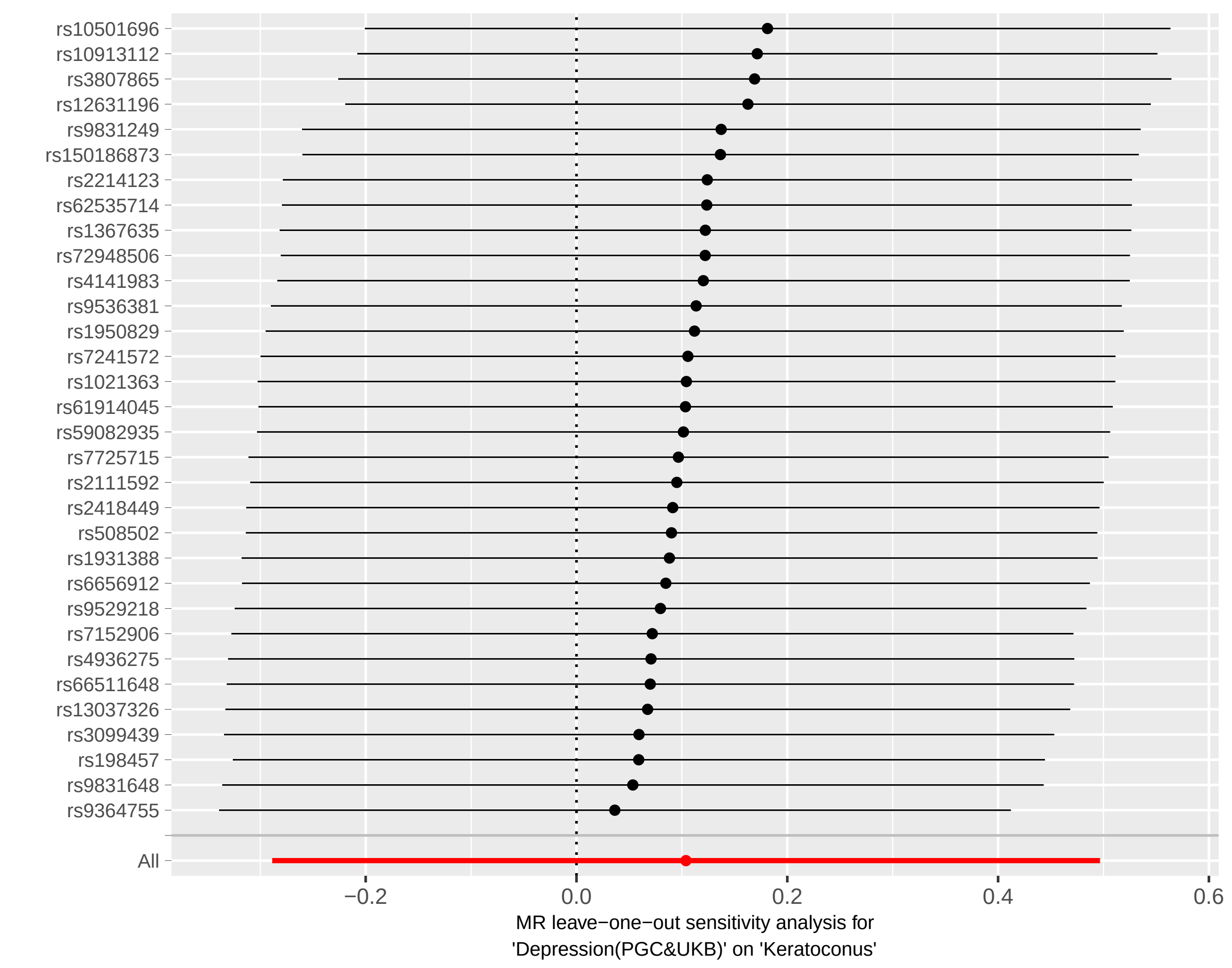
rs10501696
rs10913112
rs3807865
rs12631196
rs9831249
rs150186873
rs2214123
rs62535714
rs1367635
rs72948506
rs4141983
rs9536381
rs1950829
rs7241572
rs1021363
rs61914045
rs59082935
rs7725715
rs2111592
rs2418449
rs508502
rs1931388
rs6656912
rs9529218
rs7152906
rs4936275
rs66511648
rs13037326
rs3099439
rs198457
rs9831648
rs9364755
All
−0.2
0.0
0.2
0.4
0.6
MR leave−one−out sensitivity analysis for
'Depression(PGC&UKB)' on 'Keratoconus'

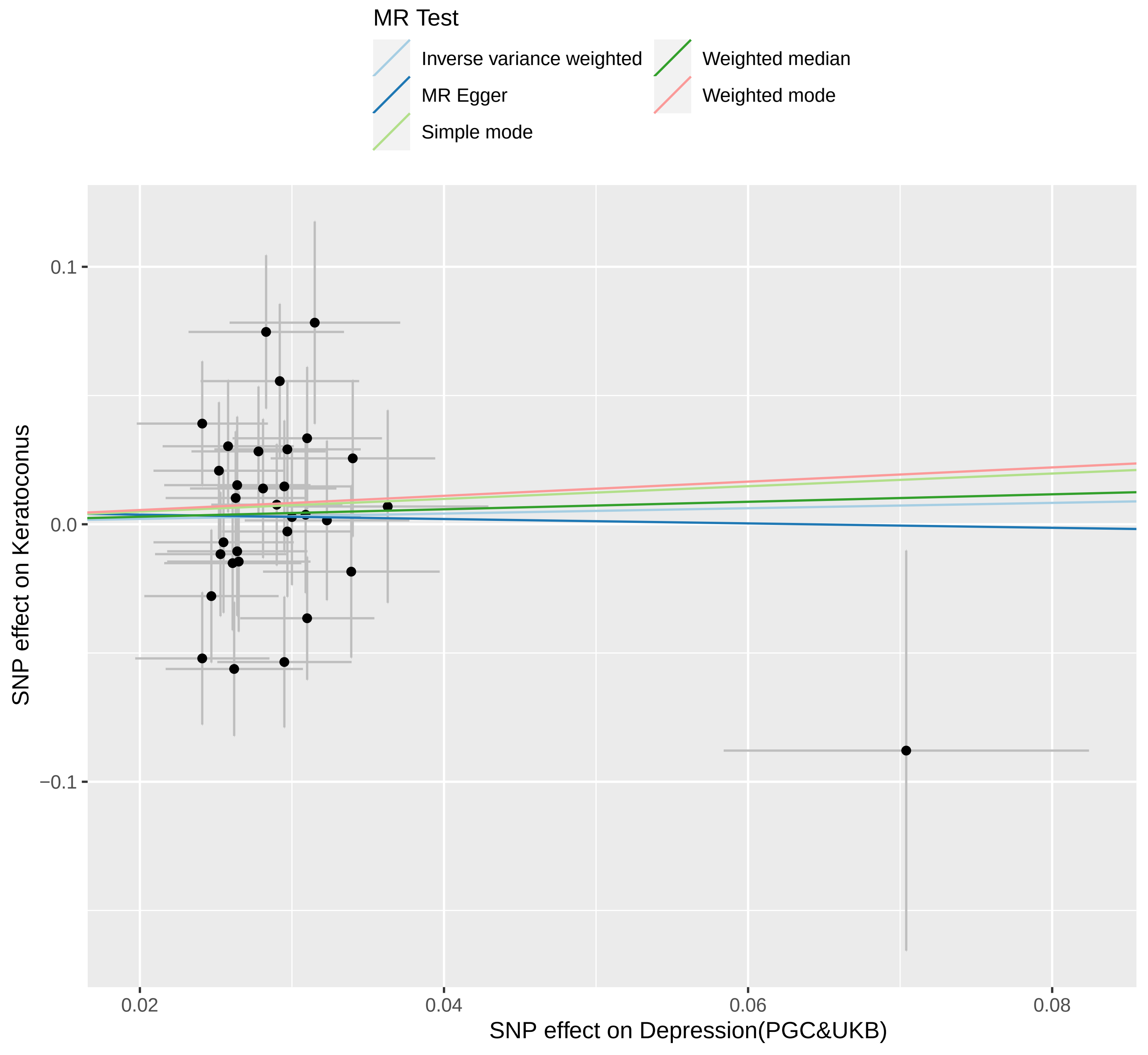
MR Test
Inverse variance weighted
MR Egger
Simple mode
Weighted median
Weighted mode
0.1
0.0
−0.1
0.02
0.04
0.06
0.08
SNP effect on Keratoconus
SNP effect on Depression(PGC&UKB)

Figure S4. MR effect size, funnel plot, Leave-one-out analysis, and scatter plot for Depression(FinnGen) on Keratoconus.

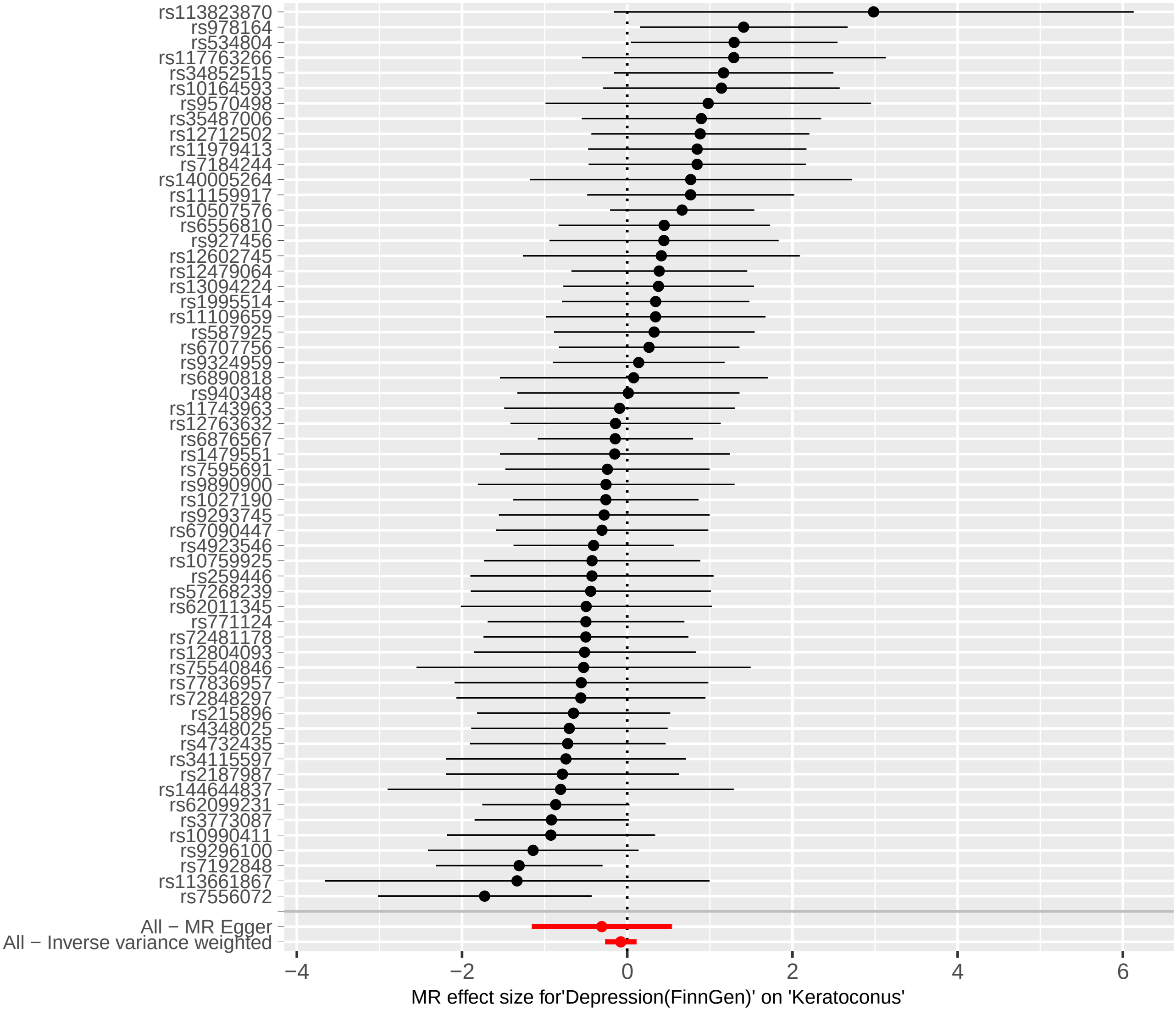

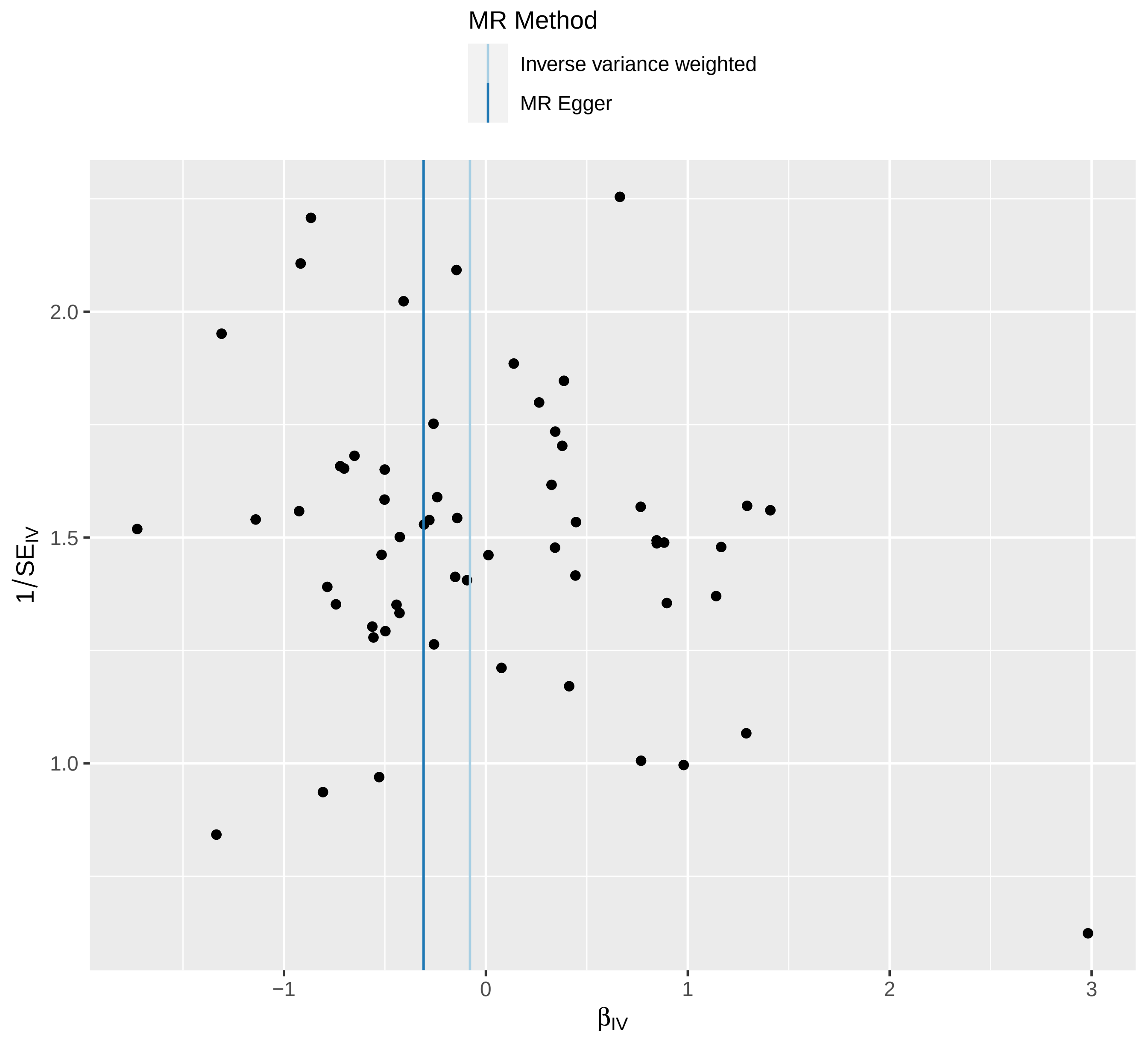
MR Method
Inverse variance weighted
MR Egger
1/SE_IV
2.0
1.5
1.0
β_IV
−1
0
1
2
3

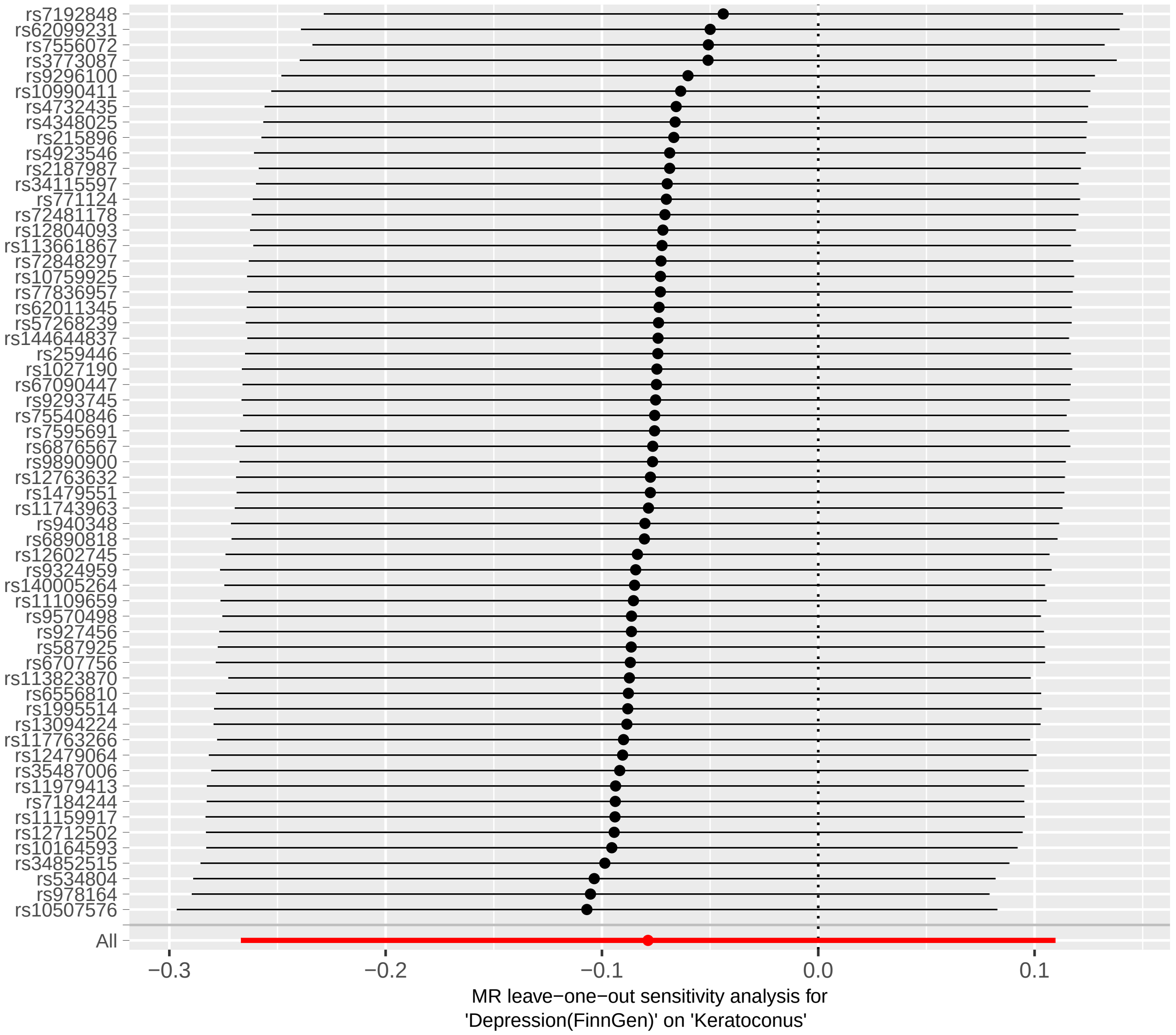
rs7192848
rs62099231
rs7556072
rs3773087
rs9296100
rs10990411
rs4732435
rs4348025
rs215896
rs4923546
rs2187987
rs34115597
rs771124
rs72481178
rs12804093
rs113661867
rs72848297
rs10759925
rs77836957
rs62011345
rs57268239
rs144644837
rs259446
rs1027190
rs67090447
rs9293745
rs75540846
rs7595691
rs6876567
rs9890900
rs12763632
rs1479551
rs11743963
rs940348
rs6890818
rs12602745
rs9324959
rs140005264
rs11109659
rs9570498
rs927456
rs587925
rs6707756
rs113823870
rs6556810
rs1995514
rs13094224
rs117763266
rs12479064
rs35487006
rs11979413
rs7184244
rs11159917
rs12712502
rs10164593
rs34852515
rs534804
rs978164
rs10507576
All
−0.3
−0.2
−0.1
0.0
0.1
MR leave−one−out sensitivity analysis for
'Depression(FinnGen)' on 'Keratoconus'

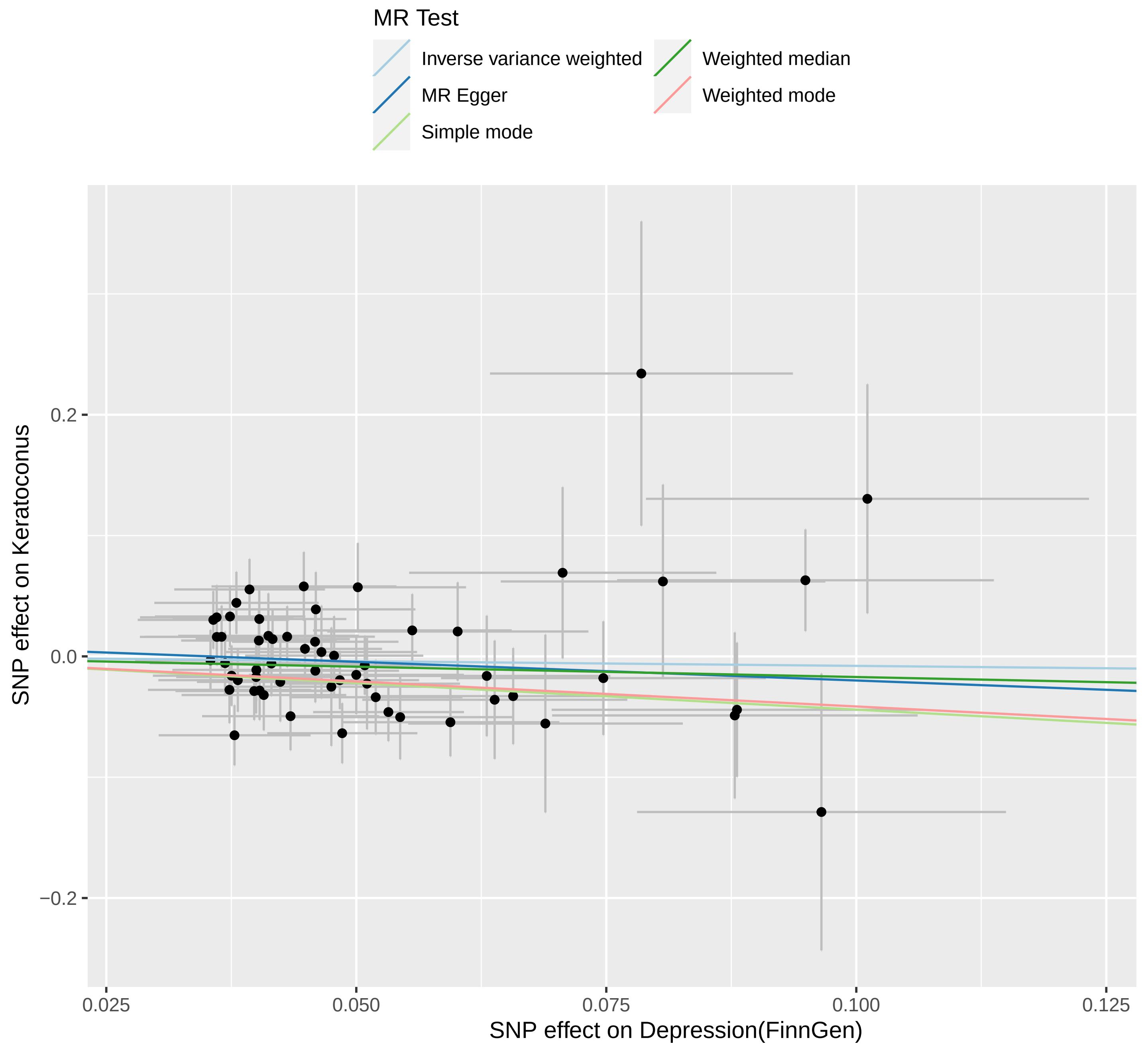

MR Test
Inverse variance weighted
MR Egger
Simple mode
Weighted median
Weighted mode
0.2
0.0
−0.2
0.025
0.050
0.075
0.100
0.125
SNP effect on Keratoconus
SNP effect on Depression(FinnGen)

Figure S5. MR effect size, funnel plot, Leave-one-out analysis, and scatter plot for Schizophrenia (PGC) on Keratoconus.

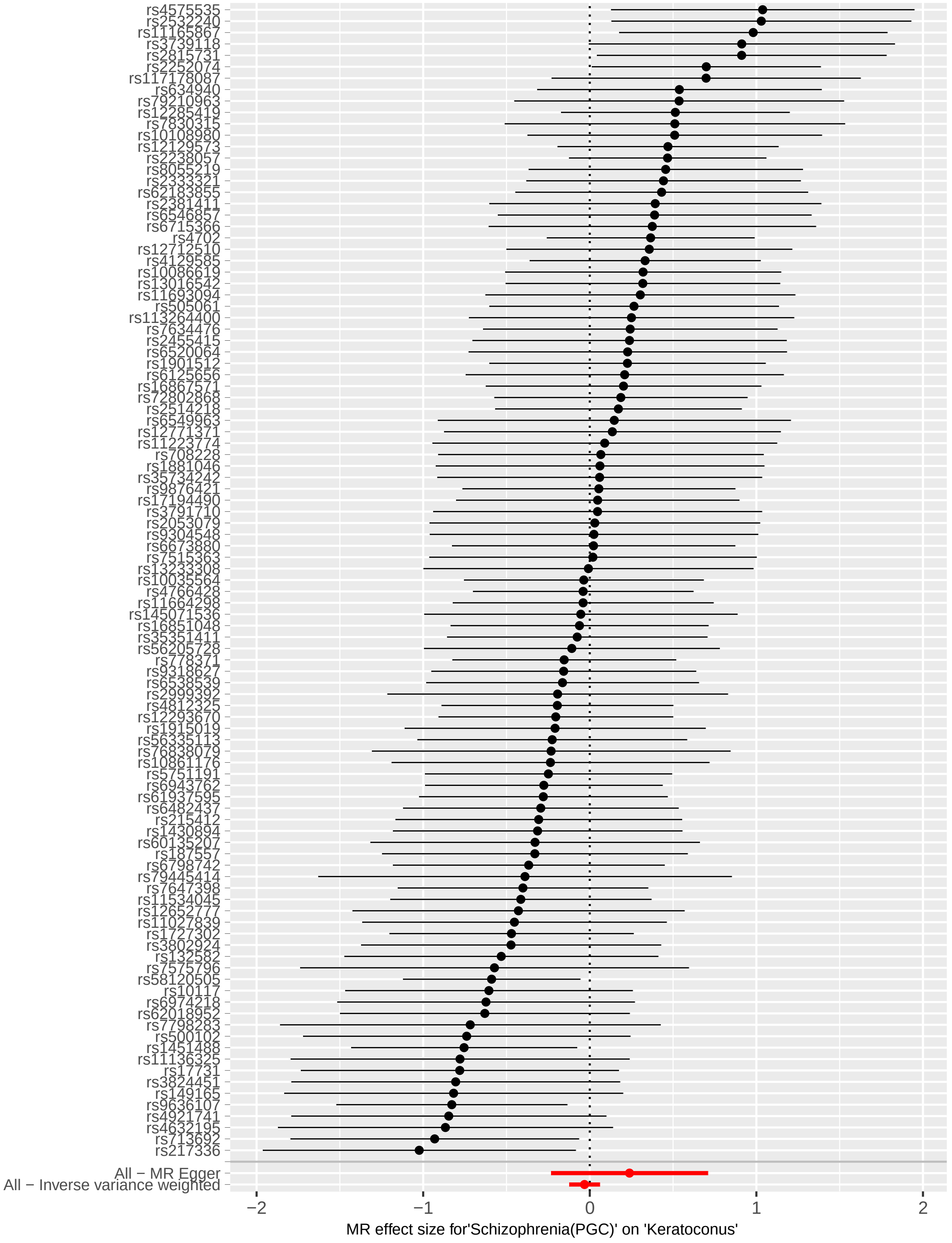

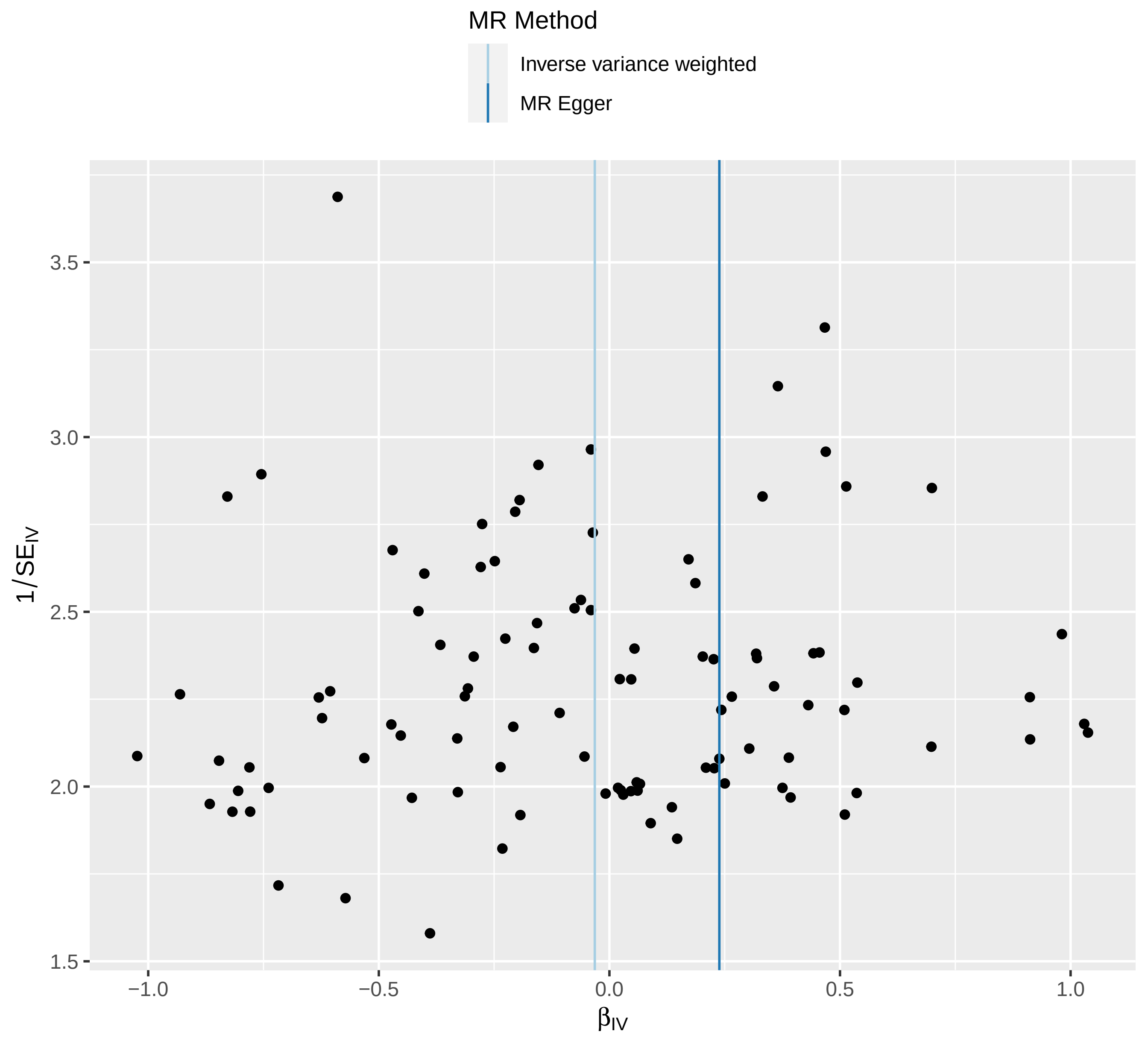
MR Method
Inverse variance weighted
MR Egger
3.5
3.0
2.5
2.0
1.5
$1/SE_{IV}$
−1.0
−0.5
0.0
0.5
1.0
β_{IV}

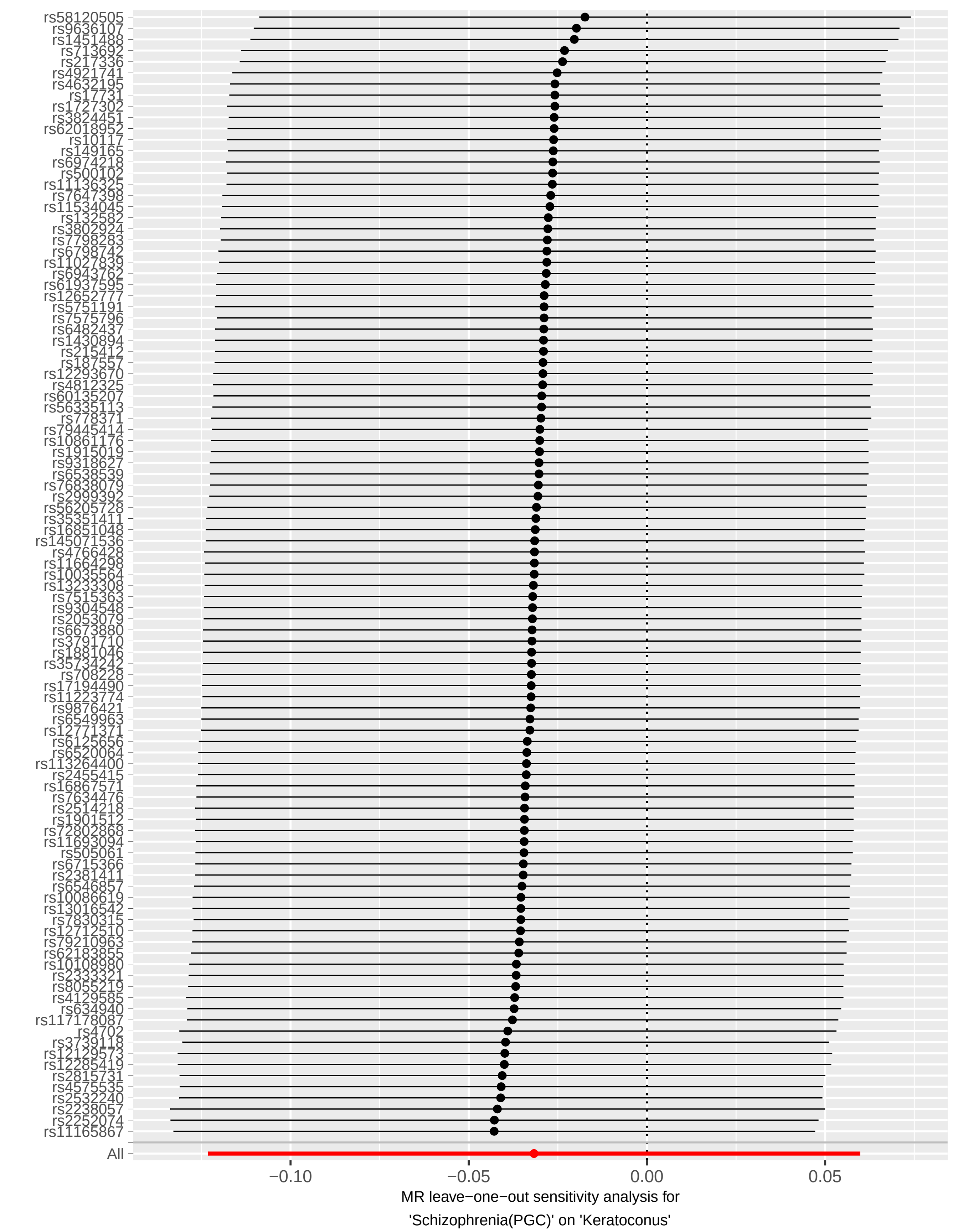
rs58120505
rs9636107
rs1451488
rs713692
rs217336
rs4921741
rs4632195
rs17731
rs1727302
rs3824451
rs62018952
rs10117
rs149165
rs6974218
rs500102
rs11136325
rs7647398
rs11534045
rs132582
rs3802924
rs7798283
rs6798742
rs11027839
rs6943762
rs61937595
rs12652777
rs5751191
rs7575796
rs6482437
rs1430894
rs215412
rs187557
rs12293670
rs4812325
rs60135207
rs56335113
rs778371
rs79445414
rs10861176
rs1915019
rs9318627
rs6538539
rs76838079
rs2999392
rs56205728
rs35351411
rs16851048
rs145071536
rs4766428
rs11664298
rs10035564
rs13233308
rs7515363
rs9304548
rs2053079
rs6673880
rs3791710
rs1881046
rs35734242
rs708228
rs17194490
rs11223774
rs9876421
rs6549963
rs12771371
rs6125656
rs6520064
rs113264400
rs2455415
rs16867571
rs7634476
rs2514218
rs1901512
rs72802868
rs11693094
rs505061
rs6715366
rs2381411
rs6546857
rs10086619
rs13016542
rs7830315
rs12712510
rs79210963
rs62183855
rs10108980
rs2333321
rs8055219
rs4129585
rs634940
rs117178087
rs4702
rs3739118
rs12129573
rs12285419
rs2815731
rs4575535
rs2532240
rs2238057
rs2252074
rs11165867
All
−0.10
−0.05
0.00
0.05
MR leave−one−out sensitivity analysis for
'Schizophrenia(PGC)' on 'Keratoconus'

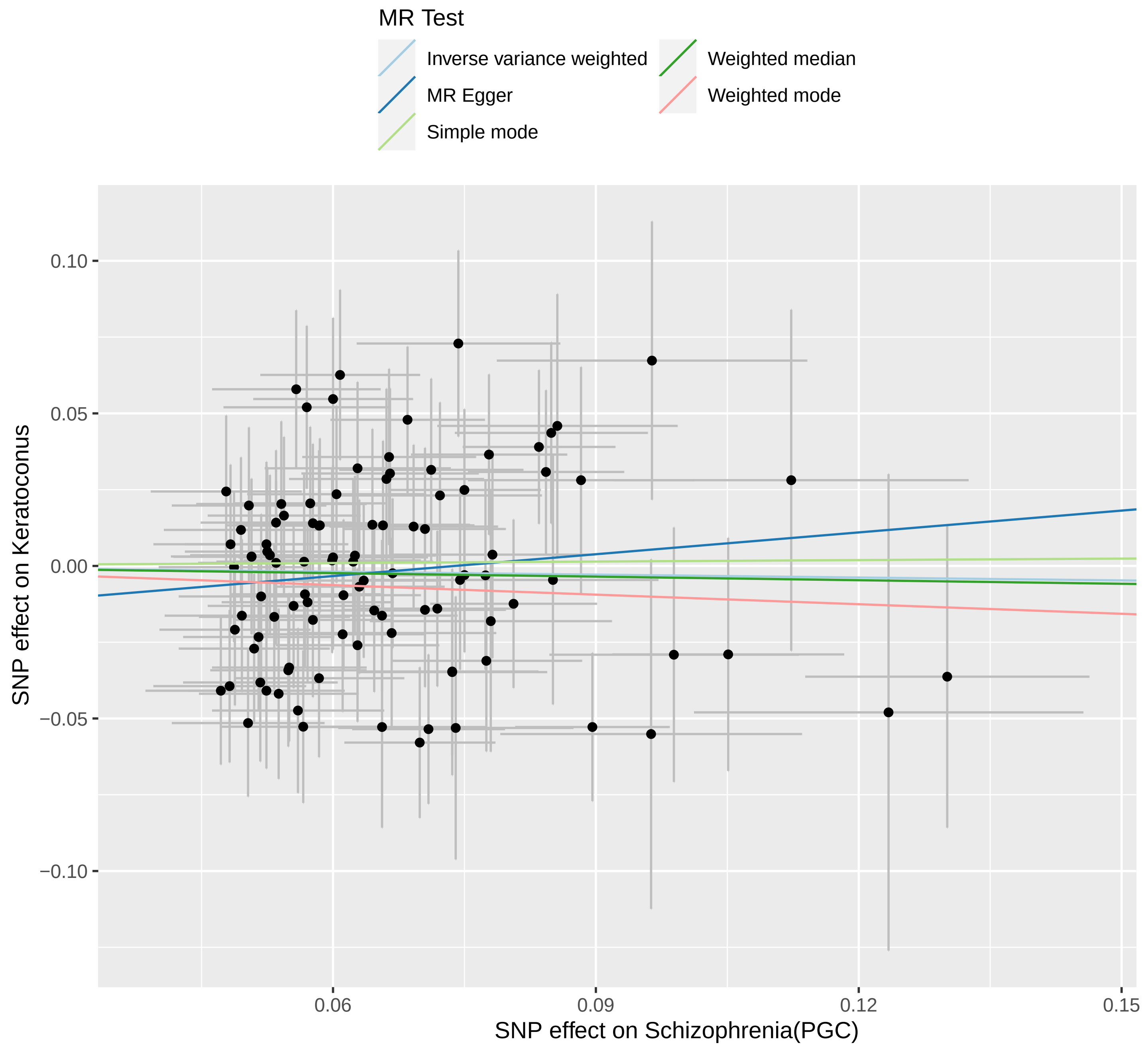
MR Test
Inverse variance weighted
MR Egger
Simple mode
Weighted median
Weighted mode
0.10
0.05
0.00
−0.05
−0.10
0.06
0.09
0.12
0.15
SNP effect on Keratoconus
SNP effect on Schizophrenia(PGC)

Figure S6. MR effect size, funnel plot, Leave-one-out analysis, and scatter plot for Schizophrenia (FinnGen) on Keratoconus.

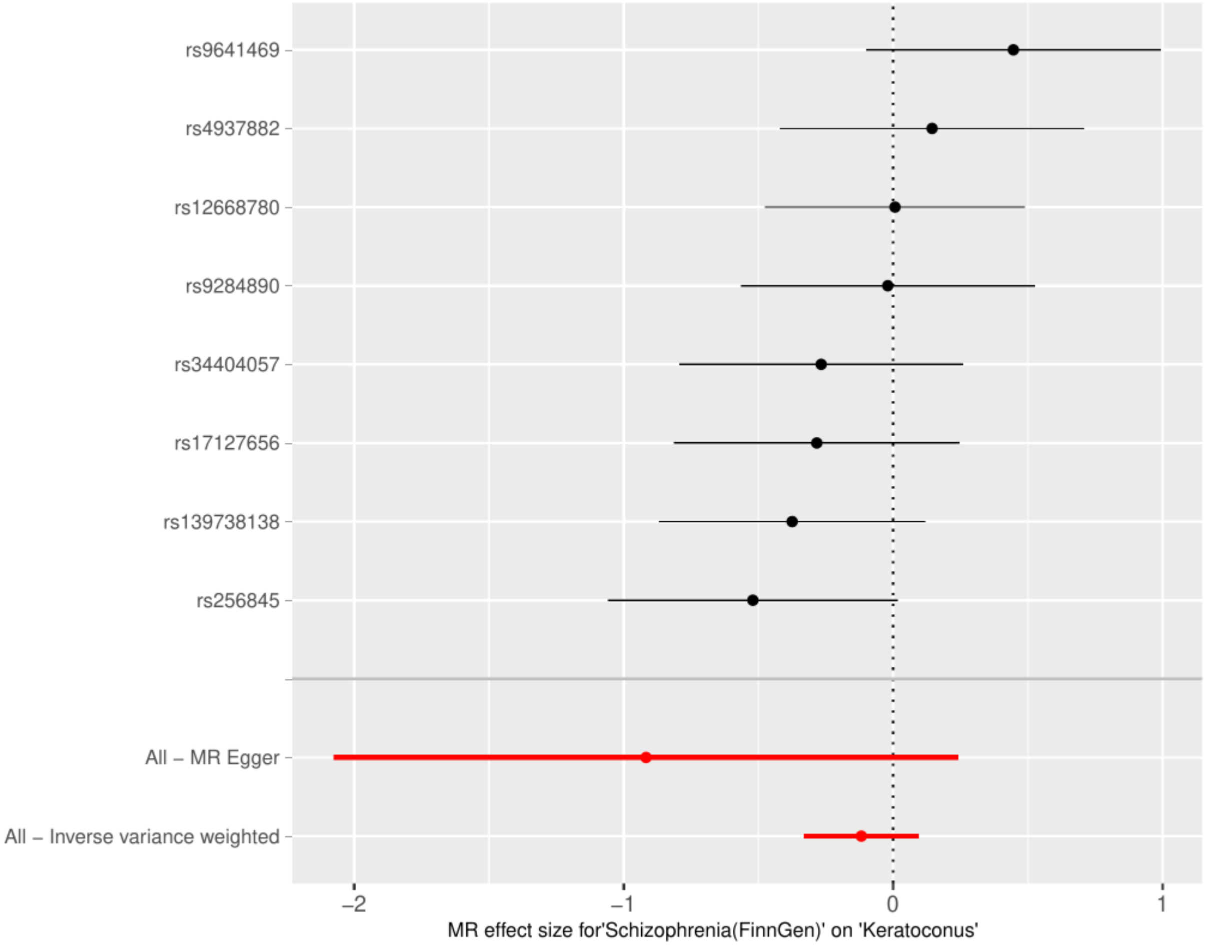

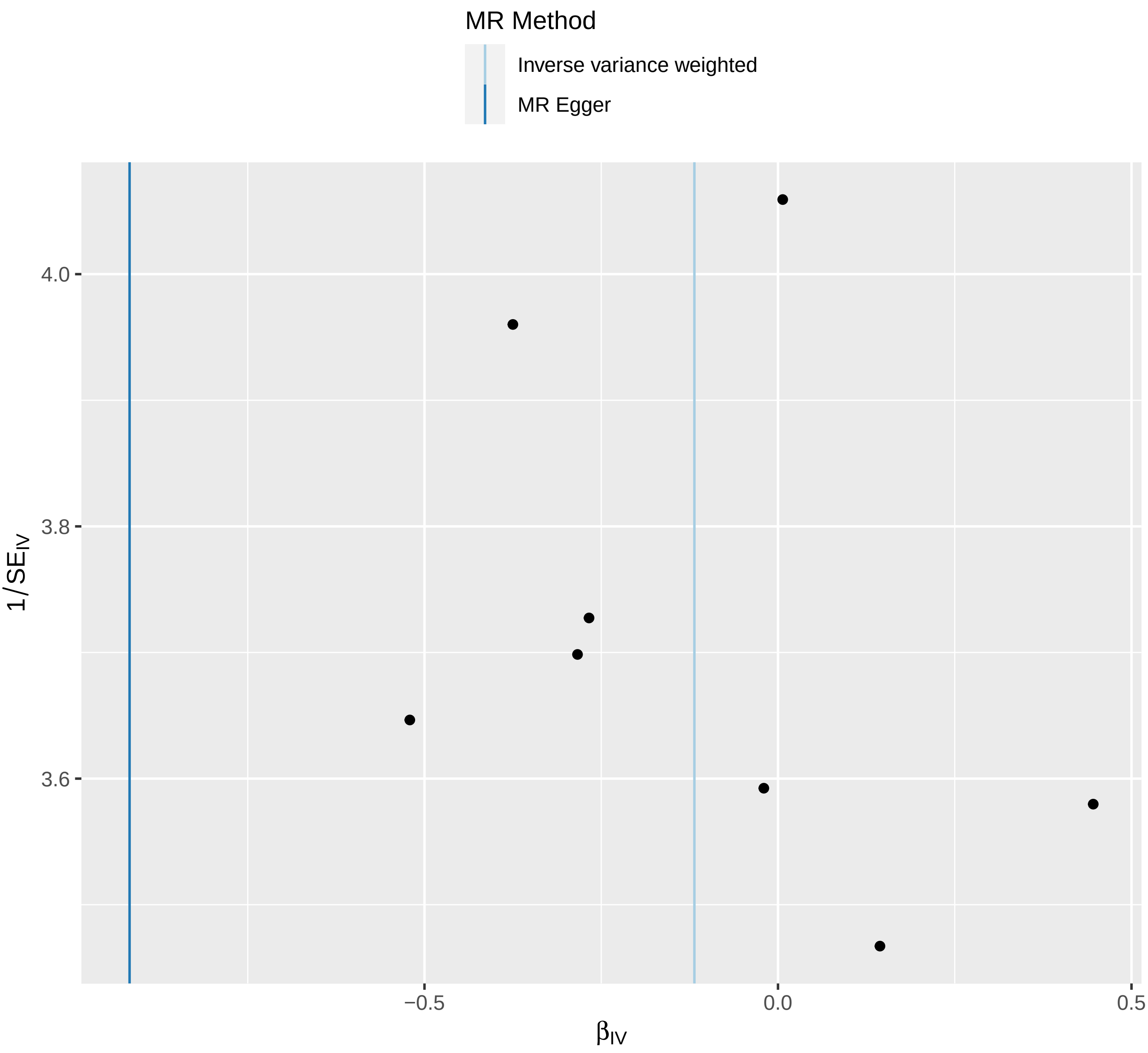
MR Method
Inverse variance weighted
MR Egger
4.0
3.8
3.6
1/SE$_{IV}$
−0.5
0.0
0.5
β$_{IV}$

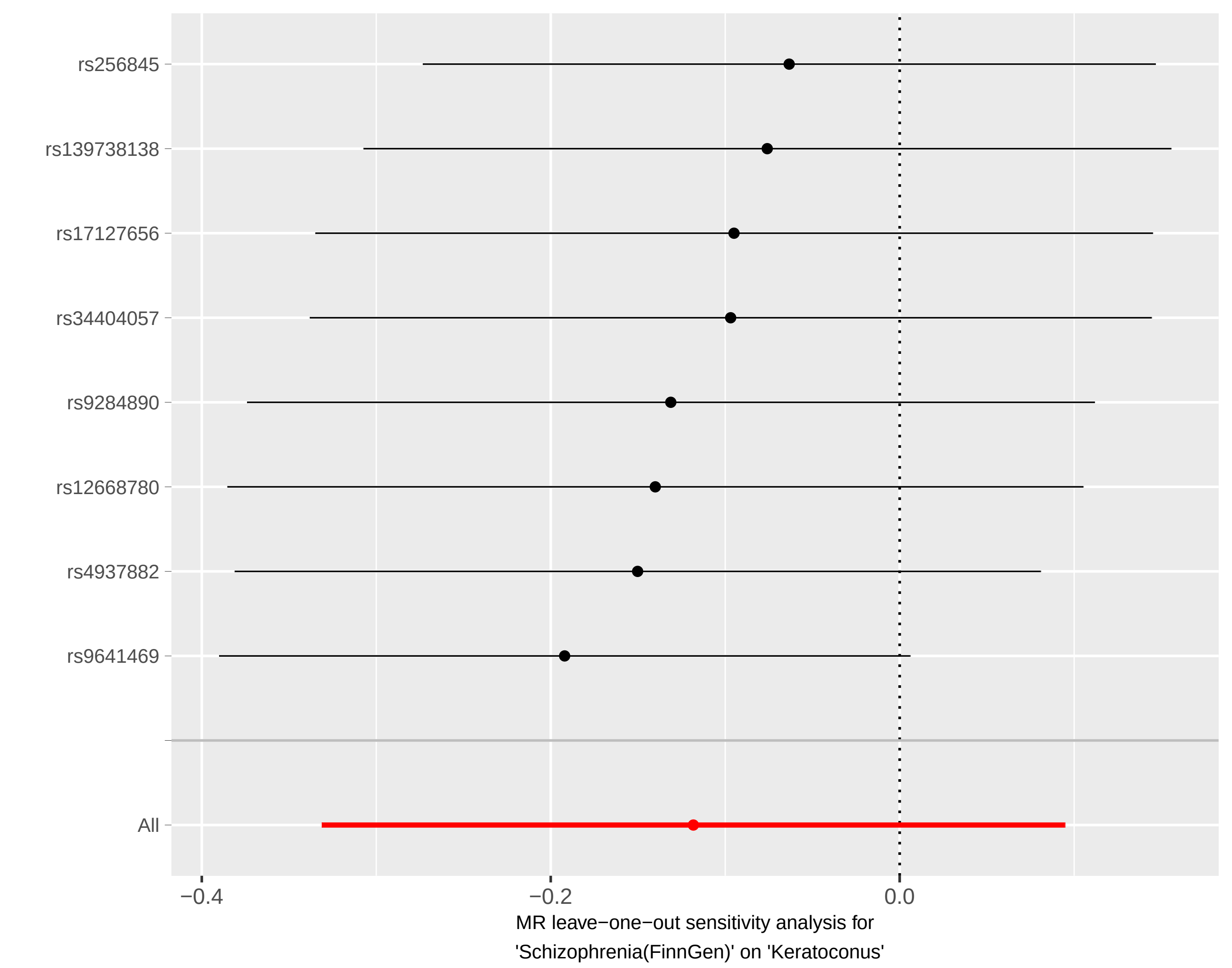

rs256845
rs139738138
rs17127656
rs34404057
rs9284890
rs12668780
rs4937882
rs9641469
All
−0.4
−0.2
0.0
MR leave−one−out sensitivity analysis for
'Schizophrenia(FinnGen)' on 'Keratoconus'

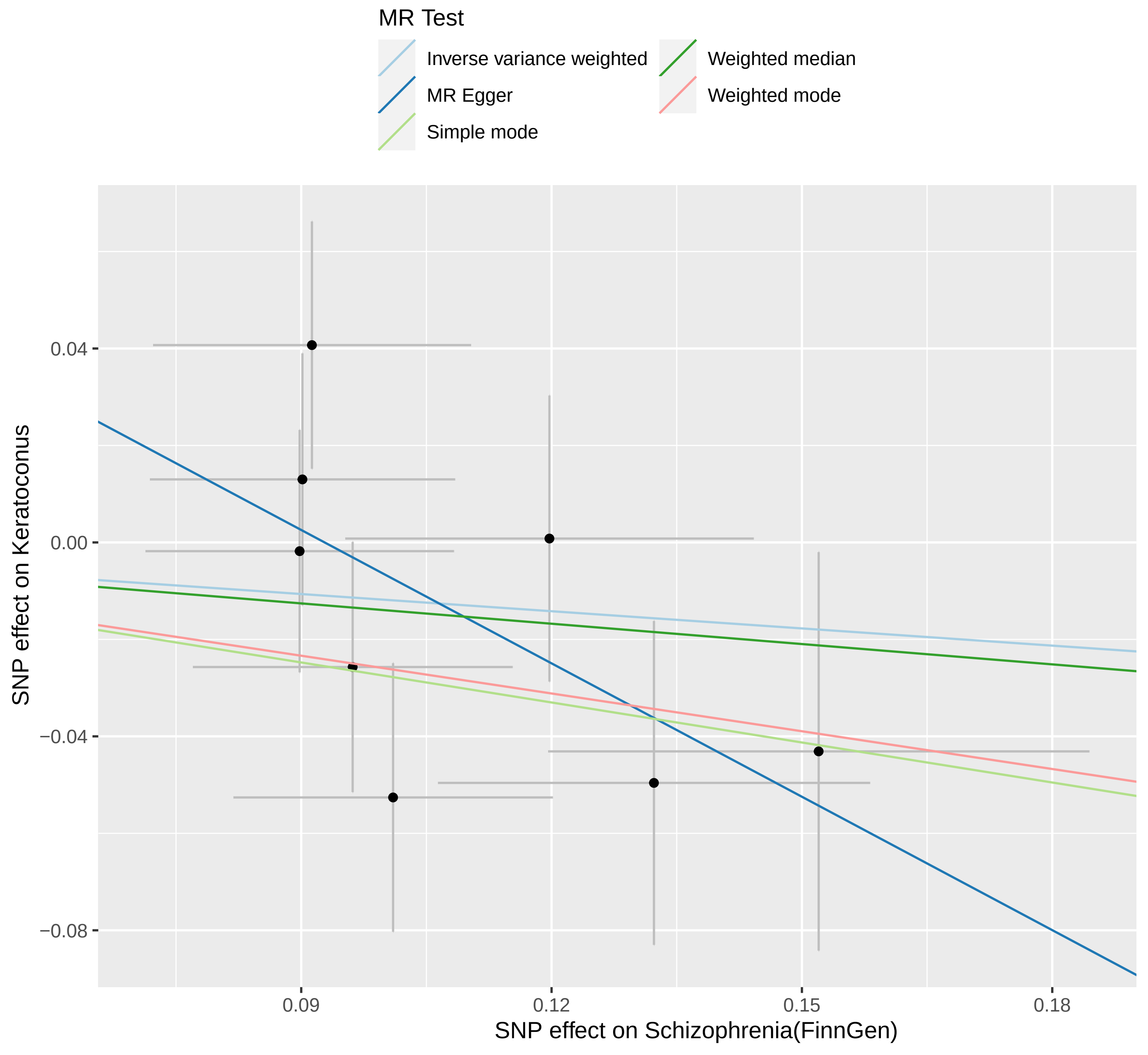
MR Test
Inverse variance weighted
MR Egger
Simple mode
Weighted median
Weighted mode
0.04
0.00
−0.04
−0.08
0.09
0.12
0.15
0.18
SNP effect on Keratoconus
SNP effect on Schizophrenia(FinnGen)
